# DLAT inhibits ferroptosis to promote malignant progression of gastric cancer through Nrf2/HO-1/GPX4 signaling pathway

**DOI:** 10.1186/s13062-026-00767-7

**Published:** 2026-04-02

**Authors:** Lin Li, Hongjing Zhao, Xiaoping Niu, Pengwei Liu

**Affiliations:** 1 Department of Gastroenterology, The First Affiliated Hospital of Wannan Medical College, Wuhu, Anhui 241001 P.R. China; 2 Department of Nephrology, The First Affiliated Hospital of Wannan Medical College, Wuhu, Anhui 241001 P.R. China

**Keywords:** Gastric cancer, DLAT, Ferroptosis, Nrf2/HO-1/GPX4 signaling pathway

## Abstract

**Background:**

Ferroptosis is a novel regulatory mechanism of cell death caused by iron-dependent lipid peroxidation (lipid-ROS) accumulation. The aim of this study was to explore whether dihydrolipoic acid transacetylase (DLAT) influences ferroptosis to promote the progression of gastric cancer (GC).

**Methods:**

The expression of DLAT in GC tissues and cell lines was examined by qRT-PCR, western blotting, and immunohistochemistry. Kaplan–Meier survival curve analysis was used to estimate overall survival, and the log-rank test was used to estimate recurrence-free survival. The functional role of DLAT in GC was evaluated using in vitro and in vivo experiments including MTT, scratch healing tests, transwell assays and a xenograft tumor mouse model. Ferroptosis was evaluated by using malondialdehyde (MDA), reduced glutathione (GSH), lactate dehydrogenase (LDH), and superoxide dismutase (SOD) detection kits, iron accumulation assays, lipid ROS quantification, and mitochondrial deep red fluorescence staining kits. Western blotting was used to detect the biomarkers of ferroptosis and the interactions between DLAT and Nrf2, HO-1 and GPX4.

**Results:**

We found that DLAT was upregulated in GC cells and tissues, and high DLAT expression indicated poor prognosis. Knockdown of DLAT inhibited the proliferation, invasion and metastasis of GC cells by increasing the levels of MDA and LDH, promoting the accumulation of Fe^2+^ and lipid ROS, consuming GSH and SOD, reducing mitochondrial membrane potential, and suppressing the expression of GPX4. Overexpression of GPX4 can specifically salvage the growth inhibition of GC cells caused by DLAT knockdown, but did not affect the activity of the upstream DLAT, Nrf2 and HO-1. The inhibition of ferroptosis by DLAT was related to the regulation of the Nrf2/HO-1/GPX4 antioxidant axis. In *vivo*, DLAT inhibited the Nrf2/HO-1 axis, hindering the growth of transplanted tumors.

**Conclusion:**

These results indicated that DLAT deficiency increased accumulation of Fe^2+^ and lipid ROS, enhanced disruption of mitochondrial membrane potential, and induced ferroptosis through the Nrf2/HO-1/GPX4 axis, thereby inhibiting the malignant progression of GC cells. DLAT serves as a prognostic biomarker and potential therapeutic target for GC.

**Trial registration:**

Registry and the Registration of the study/trial: N/A.

**Supplementary Information:**

The online version contains supplementary material available at 10.1186/s13062-026-00767-7.

## Introduction

Gastric cancer (GC) is the fifth most common cancer in the world, with more than 1 million new cases every year [1]. Despite advances in surgical techniques, radiotherapy, chemotherapy, and neoadjuvant therapy, the median survival of patients with advanced GC is less than 1 year as a result of its highly metastatic and aggressive nature [2]. Better understanding of the mechanisms underlying GC may aid in the development of targeted drugs to improve patient survival.

Dihydrolipoic acid transacetylase (DLAT), a component of the pyruvate dehydrogenase complex E2, is one of the key molecules responsible for copper reduction [3]. Several studies have shown that DLAT is involved in the pathophysiological processes of prostate cancer, chronic lymphocytic leukemia, obesity and skin homeostasis. A recent study showed that DLAT expression was upregulated in seven types of cancer: cholangiocarcinoma, esophageal cancer, hepatocellular carcinoma, lung adenocarcinoma, lung squamous cell carcinoma, gastric adenocarcinoma, GC and esophageal cancer. DLAT expression was linked to the prognosis of patients with breast invasive carcinoma, glioma, hepatocellular carcinoma and pancreatic cancer [4]. A previous report showed that the reduction of DLAT significantly reduced the proliferation ability of GC cells, suggesting the potential of targeting DLAT in the treatment of GC [5]. However, the molecular mechanisms by which DLAT exerts its effects in GC remain largely unexplored.

Ferroptosis is regulated by the nuclear factor-erythroid 2-related factor 2 (Nrf2)/hemeoxygenase-1 (HO-1) pathway [6–9]. Ferroptosis (iron-dependent cell death) is a type of cell death caused by the accumulation of iron-dependent lipid peroxidation (lipid ROS). During ferroptosis, the production of ROS increases, mitochondrial volume decreases, and membrane density increases [10–13]. Increasing studies have demonstrated the important role of ferroptosis in tumor metastasis. One study showed that lymph protects tumor cells from ferroptosis, promoting metastasis of melanoma [14]. In a mouse model of Her2-positive breast cancer, inducing ferroptosis inhibited brain metastasis [15]. GPX4, a phospholipid hydroperoxide glutathione peroxidase, is a key negative regulator of ferroptosis. GPX4 functions to maintain intracellular redox homeostasis by inhibiting lipid ROS and protecting against cell death caused by lipid ROS in cell membranes. Previous studies showed that GPX4 was highly expressed in metastatic cancer and its high expression was closely related to tumor progression [16, 17]. Lu et al. found that KLF2 regulated ferroptosis through GPX4 and inhibited the migration and invasion of cellular renal cell carcinoma [18].

In this study, we aimed to explore the role of DLAT in influencing ferroptosis in GC and the underlying mechanisms. These results might provide a new therapeutic target for GC.

## Methods

### Patients and tissue samples

This study included patients with GC at the First Affiliated Hospital of Wannan Medical College who underwent radical gastrectomy from January 2016 to June 2020. We collected eight pairs samples of fresh GC tissues and adjacent normal gastric mucosa tissues. Written informed consent was obtained from all participants. This study was in accordance with the Declaration of Helsinki Ethical Guidelines and was approved by the Institutional Ethical Review Committee of the First Affiliated Hospital of Wannan Medical College (2024-SR-75).

### Cell culture

Four GC cell lines (AGS, BGC-823, MGC-803, SGC-791) and one human gastric mucosal epithelial cell line (GES-1) were provided by the Central Laboratory of the First Affiliated Hospital of Wannan Medical College. One human embryonic kidney cell line (HEK-293T) was obtained from the Cell Bank of the Chinese Academy of Sciences in Shanghai. Cells were cultured with 1640 (Hyclone, Cytiva, USA) containing 10% FBS (Gibico, USA) + 1% antibody (Hyclone, Cytiva, USA) at 37 °C, 5% CO_2_. Cells were used in experiments when the cell confluence was 90%.

### Quantitative reverse-transcription PCR (qRT-PCR)

Total RNA was extracted from tissues and cells using TRIzol (Thermo Fisher Scientific, Shanghai, China) and analyzed for concentration and purity. Reverse transcription was performed using a reverse transcription kit (TransGen Biotech, Beijing, China). Primers were synthesized by Sangon Biotech (Shanghai, China). GAPDH mRNA was used as an endogenous control. The primer sequences were shown in Table 1. GAPDH was used as an endogenous control. The 2^−ΔΔCT^ method was used to calculate the relative expression of target genes. The reactions were carried out in triplicate.

**Table 1:** Primer sequences for qRT‒PCR

Primer sequences for qRT‒PCR	
DLAT	Forward: 5ʹ-CCGCCGCTATTACAGTCTTCC-3ʹ
	Reverse: 5ʹ-CTCTGCAATTAGGTCACCTTCAT-3ʹ
GPX4	Forward: 5ʹ-TTCACCAAGTTCCTCATCG-3ʹ
	Reverse: 5ʹ-GGCAGGTCCTTCTCTATCA-3ʹ
FTH1	Forward: 5ʹ-CCAGAACTACCACCAGGACTC-3ʹ
	Reverse: 5ʹ-GAAGATTCGGCCACCTCGTT-3ʹ
SLC3A2	Forward: 5ʹ-GGGCGTCTCGATTACCTGAG-3ʹ
	Reverse: 5ʹ-GTTCTCACCCCGGTAGTTGG-3ʹ
SLC7A11	Forward: 5ʹ-CAAGCACACTCCTCTACCA-3ʹ
	Reverse: 5ʹ-ATAAATCAGCCCAGCAACT-3ʹ
TFR-1	Forward: 5ʹ-TCGGAGAAACTGGACAGCAC-3ʹ
	Reverse: 5ʹ-ATCACGCCAGACTTTGCTGA-3ʹ
GAPDH	Forward: 5ʹ-AATCCCATCACCATCTTCCA-3ʹ
	Reverse: 5ʹ-AAATGAGCCCCAGCCTTCT-3ʹ
**The sequence for siRNA-DLAT**	
si-DLAT-1	5’‒CCCACUCUGUAUCAUUGUATT‒3’
si-DLAT-2	5’‒GCCUGGAGGAGUGUUAUAUTT‒3’
**The sequence for oe-GPX4**	
GCTAGCGCCACCATGAGCCTCGGCCGCCTTTGCCGCCTACTGAAGCCGGCGCTGCTCTGTGGGGCTCTGGCCGCGCCTGGCCTGGCCGGGACCATGTGCGCGTCCCGGGACGACTGGCGCTGTGCGCGCTCCATGCACGAGTTTTCCGCCAAGGACATCGACGGGCACATGGTTAACCTGGACAAGTA	
**The sequence for shDLAT**	
pCDH-shDLAT-1	GGATCCGCAGAGGTTGAAACTGATAAATTCAAGAGATTTATCAGTTTCAACCTCTGCTTTTTGAATTC
pCDH-shDLAT-2	GGATCCCCATACCTCATTATTACCTTTTTCAAGAGAAAAGGTAATAATGAGGTATGGTTTTTGAATTC
pCDH-shDLAT-3	GGATCCGGTTATTGCACAGCGATTAATTTCAAGAGAATTAATCGCTGTGCAATAACCTTTTTGAATTC
pCDH-NC	GGATCCTTCTCCGAACGTGTCACGTTTCAAGAGAACGTGACACGTTCGGAGAATTTTTGAATTC

### Western blotting (WB)

Tissues and cells were lysed with RIPA lysis buffer (Beyotime Institute of Biotechnology, Shanghai, China) and protein was quantified with a BCA protein quantification kit (Solarbio, Beijing, China). The protein samples were separated by sodium dodecyl sulfate-polyacrylamide gel electrophoresis and transferred to a polyvinylidene difluoride membrane. Then the membrane was incubated with primary antibody at 4 °C overnight. Membranes were then incubated with horseradish peroxidase enzyme-labeled secondary antibody. Membranes were washed in PBS for 3–5 times for 5 min between each step. Bands were visualized using ECL luminescent solution and images were obtained using an automatic chemiluminescence analyzer. The primary and secondary antibodies used were as follows: anti-DLAT antibody (1:1000, 13426-1-AP, AB_2091774, Proteintech, America), anti-FTH1 antibody (1:1000, YT1692, AB_3719823, Immunoway, America), anti-GPX4 antibody (1:1000, YN3047, AB_3719820, Immunoway, America), anti-SLC3A2 antibody (1:1000, YT5599, AB_3719822, Immunoway, America), anti-SLC7A11 antibody (1:1000, YT8130, AB_3719821, Immunoway, America), anti-TFR1 antibody (1:1000, ab214039, AB_3719819, Abcam, UK), anti-Nrf2 antibody (1:1000, YM4294, AB_3719824, Immunoway, America), anti-HO-1 antibody (1:1000, YM33079, AB_3719825, Immunoway, America), and anti-tubulin antibody (1:5000, 10094-1-AP, AB_2210695, Proteintech, America).

### Immunohistochemistry (IHC)

Tissue sections were dehydrocarbonated in xylene and rehydrated in a series of ethanol solutions. Antigen was retrieved by microwave heat induction and sections were incubated with 3% H_2_O_2_ to quench endogenous peroxidase. The sections were incubated overnight with the primary antibody DLAT (1:1000, 13426-1-AP, AB_2091774, Proteintech, America) at 4 °C. After washing in PBS, the sections were then incubated with HRP-conjugated secondary antibody. After another wash, the sections were incubated with DAB chromogenic reagent and nuclei were counterstained with hematoxylin. Sections were examined under a upright bright-field microscope (Nikon Ci-L).

Staining was evaluated by two independent pathologists at or above the associate senior level, and the pathologists were blinded to the samples. The staining intensity was scored as follows: 0, negative; 1, weak; 2, moderate; and 3, strong. The percentage of positively stained cells was scored as follows: 0, 0%; 1, 1%–10%; 2, 11%–50%; and 3, > 50%. The final score was obtained by multiplying both scores and ranged from 0 to 9. Sections with scores > 3 and ≤ 3 were defined as having high and low DLAT expression, respectively.

### siRNAs, plasmids, shRNAs, transfection and stable cell lines

DLAT-specific (siRNA-DLAT) and negative control (siRNA-NC) siRNA duplexes were purchased from Jima Biological Co., Ltd (Shanghai, China). The human GPX4 gene sequence (NM_001039847.3) was synthesized (General Biol) and subcloned into the pcDNA3.1(+) plasmid to generate pcDNA-GPX4 (designated as oe-GPX4). Lipofectamine 2000 was used for cell transfection following specific protocols. The above sequences are shown in Table 1.

For stable cell lines, three shRNA sequences targeting the DLAT gene (NCBI human DLAT gene sequence, NM_001931) and a paired negative control (NC) were designed and synthesized by Anhui General Biology Company. The sequences are shown in Table 1. The shRNA sequences were inserted into the BamHI and EcoRI sites of the pCDH-U6-shRNA-EF1-Puro vector, which harbors a puromycin resistance gene, to generate the shRNA-DLAT and sh-vector. The Endotoxin-free Plasmid Bulk Extraction Kit (Beijing, China) was used for plasmid preparation. Recombinant lentiviruses encoding shRNA-DLAT or the sh-vector control were produced by transfecting plasmids into 293T cells using Lipofectamine 2000 (Invitrogen, 11668-019) following the manufacturer’s instructions. MGC-803 cell lines were transfected with lentiviruses using polybrene (8 µg/mL) for 48–72 h. Stably transduced cells were selected by growth in medium containing 5 µg/mL puromycin for 2 weeks. Cells were harvested for subsequent analysis.

### Methyl thiazolyl tetrazolium (MTT) assay

GC cells in 96-well plates in logarithmic growth were transfected with siRNAs. After 48 h, 10 µl MTT solution (20 mg/ml) was added to each well. After 4 h, 100 µl dimethyl sulfoxide was added to each well. The absorbance at 490 nm was then measured, with six replicates per group.

### Wound-healing assays

Transfected GCs were inoculated into 12-well plates. When monolayer cells covered the bottom of the plate, a toothpick was used to generate a scratch along the bottom. Measurements of the wound width were taken before (0 h) and after 24 h of incubation at 37 °C using ImageJ software.

### Transwell migration and invasion assays

For migration assays, a Transwell chamber was placed in a 24-well plate; the prepared transfection reagent and cell mixture were added to the top chamber. After 48 h, the medium was removed and cells were fixed with 4% paraformaldehyde for 20 min. After staining with 0.1% crystal violet solution for 15 min and washing with PBS, cell imaging and counting were performed under a microscope.

For invasion assays, the Transwell chamber was first coated with Matrigel matrix gel. The subsequent steps were the same as those for the migration assay.

### Detection of oxidative stress

Reagents for the assessment of malondialdehyde (MDA, BC0025), lactate dehydrogenase (LDH, BC0685), superoxide dismutase (SOD, BC5165), and glutathione (GSH, BC1175) levels were obtained from Solarbio Technology Co., Ltd., Beijing, China. All experiments were performed following the manufacturer’s instructions.

### Lipid ROS quantification

The lipid ROS detection kit (Beyotime, BDPY 581/591 C11, S0043S, China) was used to detect the changes in the degree of lipid ROS within the cells with BDPY 581/591 C11 as the fluorescent probe. When lipid ROS occurs, the intensity of red fluorescence in living cells decreases while the intensity of green fluorescence increases. The fluorescence intensity of lipid ROS was quantitatively determined by ImageJ software. The degree of lipid ROS was evaluated using the oxidation rate (corrected green fluorescence intensity / (corrected green fluorescence intensity + corrected red fluorescence intensity)).

### Iron accumulation assays

The Fe^2+^ level was detected by the iron ion detection kit (Solarbio, BC5410, China) and the cell iron ion red fluorescence detection kit (Beyotime, RhoNox-6, S1070S, China). The operation steps were followed according to the instructions. The determination of Fe^2+^ content is carried out by measuring the absorbance value with an enzyme reader and then calculating the Fe^2+^ content in the sample. For fluorescence stain, if the intracellular content of Fe^2+^ increases, the red fluorescence intensity after RhoNox-6 staining will significantly enhance; conversely, the red fluorescence intensity will weaken. The fluorescence intensity of the red fluorescent signal of Fe^2+^ in cells was quantitatively analyzed using the ImageJ software.

### Mitochondrial fluorescence staining

The mitochondrial fluorescence staining was performed according to the operation steps of the mitochondrial deep red fluorescence staining kit (Beyotime, Mito-Tracker Deep Red FM, C1998S, China). The mitochondria show red fluorescence, while the cell nucleus shows blue fluorescence. When ferroptosis occurs, the mitochondrial membrane potential decreases, and the red fluorescence of mitochondria significantly reduces. The fluorescence intensity was quantitatively determined by ImageJ software.

### Mouse xenograft model

Nude mice were purchased from Hangzhou Ziyuan Experimental Animal Technology Co., Ltd., China. Six-week-old male mice were randomly divided into two groups: the shRNA-DLAT group and the sh-vector group (*n* = 6/group). MGC-803 cells transfected with shRNA-DLAT or sh-vector (1 × 10^7^ cells) were subcutaneously injected into the right axilla of mice. Tumor growth was monitored, and the length and width of the tumor were measured with a vernier caliper every three days. The mice were sacrificed 16 days after cell injection. The tumors were removed, and the weight and volume were recorded. Tumors were then subjected to subsequent analyses. The protein expression levels of DLAT, Nrf2 and HO-1 in xenografts were then detected by WB. The animal studies were performed in accordance with the animal experiment guidelines, and approved by the Animal Protection Committee of Wannan Medical College (WNMC-AWE-2024159).

### Statistical analysis

GraphPad Prism 8 (GraphPad Software, La Jolla, CA, USA) and SPSS 26.0 (SPSS Inc., Chicago, IL, USA) were used for data processing and statistical analysis. Measurement data are shown as mean ± standard deviation. The T-test was performed for comparisons between two groups, and one-way analysis of variance was used for comparisons among multiple groups (LSD-t test was performed for pairwise comparisons within each group). Univariate and multivariate Cox regression analyses were conducted to explore the independent risk factors for prognosis. Kaplan–Meier survival curves were used to estimate the overall survival (OS) and recurrence-free survival using the log-rank test. In vitro experiments were repeated at least three times. *P* < 0.05 indicated statistical significance.

## Results

### The expression level of DLAT is increased in GC cell lines and tissues

**Fig. 1:**
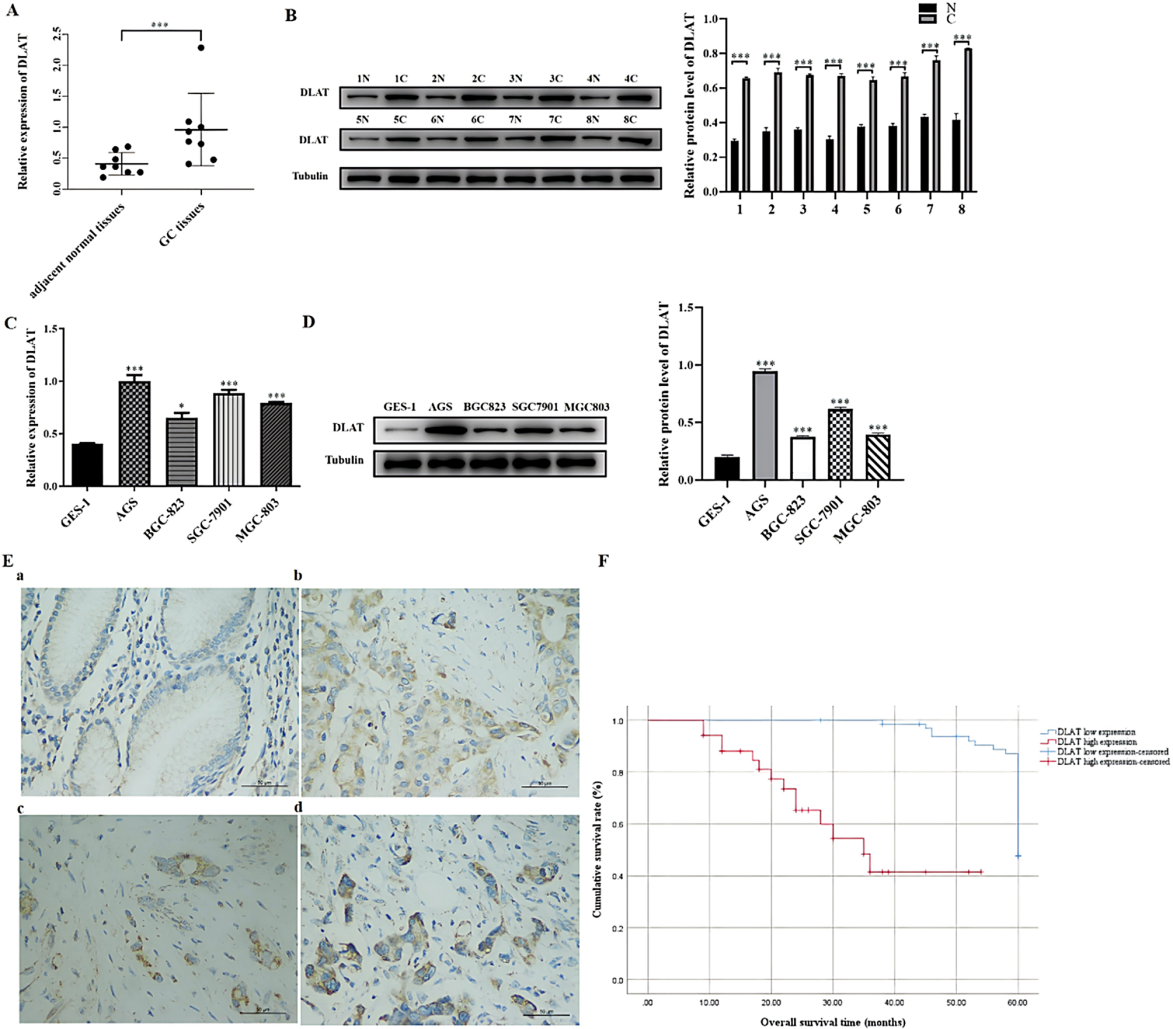
DLAT upregulation in GC cell lines and primary gastric tumors. **(A)** mRNA expression of DLAT in paired GC and adjacent non-tumorous mucosa. **(B)** Protein expression of DLAT in paired GC (C) and adjacent non-tumorous mucosa (N). **(C)** mRNA expression of DLAT in each cell line. **(D)** Protein expression of DLAT in each cell line. **(E)** Representative images of DLAT immunohistochemistry in GC (×400), (a) negative, (b) low positive, (c) mild positive, (d) strong positive. **(F)** Kaplan-Meier survival analysis of overall survial in GC patients based on DLAT expression. *: *P* < 0.05, ***:*P* < 0.001

qRT-PCR and WB results showed that the levels of DLAT mRNA (Fig. 1A, C) and protein (Fig. 1B, D) were significantly higher in GC tissues and GC cell lines (AGS, BGC-823, SGC-7901, and MGC-803) than in adjacent normal tissues and a human normal gastric mucosal cell line (GES-1) (*P* < 0.05).

We next performed IHC of DLAT in tumor specimens from patients with GC; representative images are shown in Fig. 1E. Patients were categorized into the DLAT high-expression group (*n* = 34) and low-expression group (*n* = 66). Univariate analysis showed that high expression of DLAT was closely related to tumor location, depth of invasion, presence of ulcers, and nerve and vascular invasion (*P* < 0.01), but not related to age, gender, tumor size, degree of differentiation, or lymph node metastasis (*P* > 0.05) (Table 2). Multivariate analysis results showed that high expression of DLAT was an independent risk factor for tumor location and vascular invasion (Table 3). For patients with high DLAT expression, the risks of GC in the proximal and middle stomach were 0.192 times [*P* = 0.007, OR = 0.192 (95% confidence interval (CI): 0.057–0.640)] and 0.207 times [*P* = 0.042, OR = 0.207 (95%CI: 0.045–0.945)] that risk of GC in the distal stomach. The risk of vascular infiltration in the DLAT high-expression group was 4.88 times that of the DLAT low-expression group [*P* = 0.003, OR = 4.880 (95%CI: 1.723–13.819)].

**Table 2:** Univariate analysis of clinicopathological features and DLAT expression levels in GC patients

Characteristic	DLAT low expression (*n* = 66)	DLAT high expression (*n* = 34)	*P* value
**Age**,** years**			0.263
≦60	31	20	
>60	35	14	
**Sex**			0.928
Male	51	26	
Female	15	8	
**Tumor location**			0.003*
Proximal stomach	5	9	
Middle stomach	3	5	
Distal stomach	58	20	
**Tumor size (cm)**			0.148
< 2	16	4	
≧ 2	50	30	
**Depth of invasion**			0.002*
T1	29	3	
T2	12	14	
T3	14	14	
T4	11	3	
**Differentiation**			0.305
Poorly differentiated	18	10	
Moderately differentiated	27	6	
Medium - low differentiation	21	18	
**Ulcer finding**			0.003*
Absence	27	3	
Presence	39	31	
**Vascular tumor thrombus**			< 0.001*
Absence	51	11	
Presence	15	23	
**Neural invasion**			< 0.001*
Absence	43	8	
Presence	23	26	
**Lymph node metastasis**			0.263
Absence	45	7	
Presence	21	27	

**Table 3:** Multivariate analysis of clinicopathological features and DLAT expression levels in GC patients

Characteristic	B	S.E.	Wald	*P*	OR	95%CI
Tumor location	-0.745	0.355	4.397	0.036*	0.475	0.237–0.953
Depth of invasion	-0.081	0.328	0.061	0.805	0.922	0.485–1.754
Ulcer finding	0.789	0.862	0.838	0.008	2.201	0.407–11.910
Vascular tumor thrombus	1.585	0.531	8.910	0.003*	4.880	1.723–13.819
Neural invasion	1.027	0.643	2.554	0.110	2.892	0.792–9.838
Constant	-3.829	1.842	4.320	0.038		

Kaplan–Meier survival analysis revealed that the median OS of the DLAT high- and low-expression groups was 36.2 months and 58.6 months, respectively, revealing that high DLAT expression was associated with poor prognosis (χ2 = 41.629, *P <* 0.001, Fig. 1F). Univariate Cox regression analyses showed that depth of invasion, degree of differentiation, presence of ulcers, nerve and vascular invasion, and high DLAT expression were risk factor for OS in patients with GC (Table 4). Furthermore, multivariate Cox regression analyses showed that vascular invasion, and high DLAT expression were independent prognostic factors for OS in patients with GC in our study (Table 5).

**Table 4:** Univariate and multivariate cox regression analyses for overall survival in patients with GC

Variable	Overall survival	
	*n*	*P* value
**Age**,** years**		0.146
≦ 60	51	
> 60	49	
**Sex**		0.799
Male	77	
Female	23	
**Tumor location**		0.093
Proximal stomach	14	
Middle stomach	8	
Distal stomach	78	
**Tumor size (cm)**		0.215
< 2	20	
≧ 2	80	
**Depth of invasion**		0.018*
T1	32	
T2	26	
T3	28	
T4	14	
**Differentiation**		0.034*
Poorly differentiated	28	
Moderately differentiated	33	
Medium - low differentiation	39	
**Ulcer finding**		0.020*
Absence	30	
Presence	70	
**Vascular tumor thrombus**		< 0.001*
Absence	62	
Presence	38	
**Neural invasion**		0.002*
Absence	51	
Presence	49	
**Lymph node metastasis**		0.303
N0	52	
N1	10	
N2	17	
N3	21	
**DLAT expression**		< 0.001*
Low expression	66	
High expression	34	

**Table 5:** Multivariate cox regression analyses for overall survival in patients with GC

Variable	B	S.E.	Wald	*P*	OR	95%CI
Depth of invasion	0.555	0.406	4.796	0.187	1.743	0.786–3.862
Differentiation	0.508	0.369	5.122	0.077	1.662	0.806–3.428
Ulcer finding	-0.570	0.534	1.140	0.286	0.566	0.199–1.610
Vascular tumor thrombus	0.798	0.328	5.920	< 0.001*	3.173	1.774–5.672
Neural invasion	-0.705	0.512	1.896	0.169	0.494	0.181–1.348
DLAT expression	1.443	0.341	17.957	< 0.001*	4.234	2.172–8.252

Together, these results indicated that DLAT expression was increased in GC cell lines and tissues and increased DLAT expression was associated with poor prognosis in patients with GC.

### Downregulation of DLAT reduces proliferation, invasion, and metastasis of GC in vitro

**Fig. 2:**
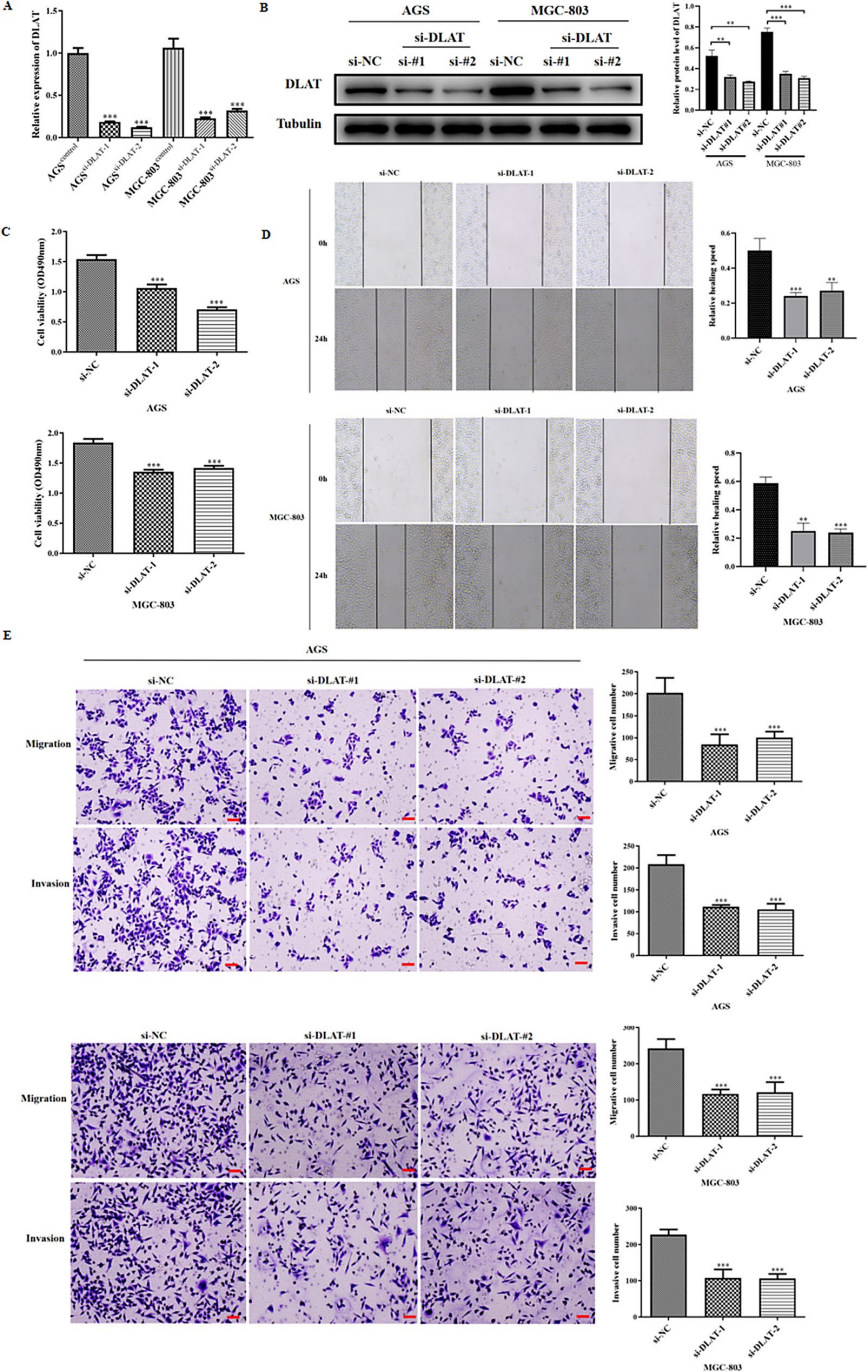
Knockdown of DLAT inhibited cell proliferation, invasion and metastasis in GC cell lines AGS and MGC803. **(A)** Transfection of si-DLAT-#1 and si-DLAT-#2 successfully reduced DLAT mRNA expression in AGS and MGC803 cells. **(B)** Transfection of si-DLAT-#1 and si-DLAT-#2 successfully reduced DLAT protein expression in AGS and MGC803 cells. **(C)** Proliferation ability was significantly decreased after knockdown of DLAT. **(D)** Healing rate was significantly decreased after knockdown of DLAT. **(E)** Invasive and migratory ability was significantly decreased after knockdown of DLAT. **:*P* < 0.01, ***: *P* < 0.001

We selected AGS and MGC-803 cells for functional experiments because of the relatively higher DLAT expression levels in these cell lines. We used siRNA to downregulate DLAT, and qRT-PCR and WB confirmed that si-DLAT significantly reduced the expression level of DLAT mRNA and protein in both cell lines compared with si-NC (Fig. 2A–B).

We next explored the influence of DLAT on the malignant behavior of GC cells. MTT assays showed that si-DLAT significantly inhibited the viability of AGS and MGC-803 cells (*P* < 0.001, Fig. 2C). Wound-healing assays and Transwell assays showed that the numbers of migrated and invading cells in the si-DLAT group were significantly reduced compared with the control group (*P* < 0.01, Fig. 2D–E).

### DLAT inhibites ferroptosis in GC cells

In this study, AGS GC cells were treated by knocking down DLAT, adding ferrostatin-1 and erastin to evaluate whether DLAT affected ferroptosis in GC cells. Since the interference efficiency of si-DLAT-#2 in AGS GC cells was superior to that of si-DLAT-#1, we used si-DLAT-#2 for the subsequent experiments. The concentration of ferrostatin-1 was selected as 4.0 μm for subsequent experiments. Erastin was used as a ferroptosis inducer at a concentration of 10 μm for the subsequent experiments [19].

**Fig. 3:**
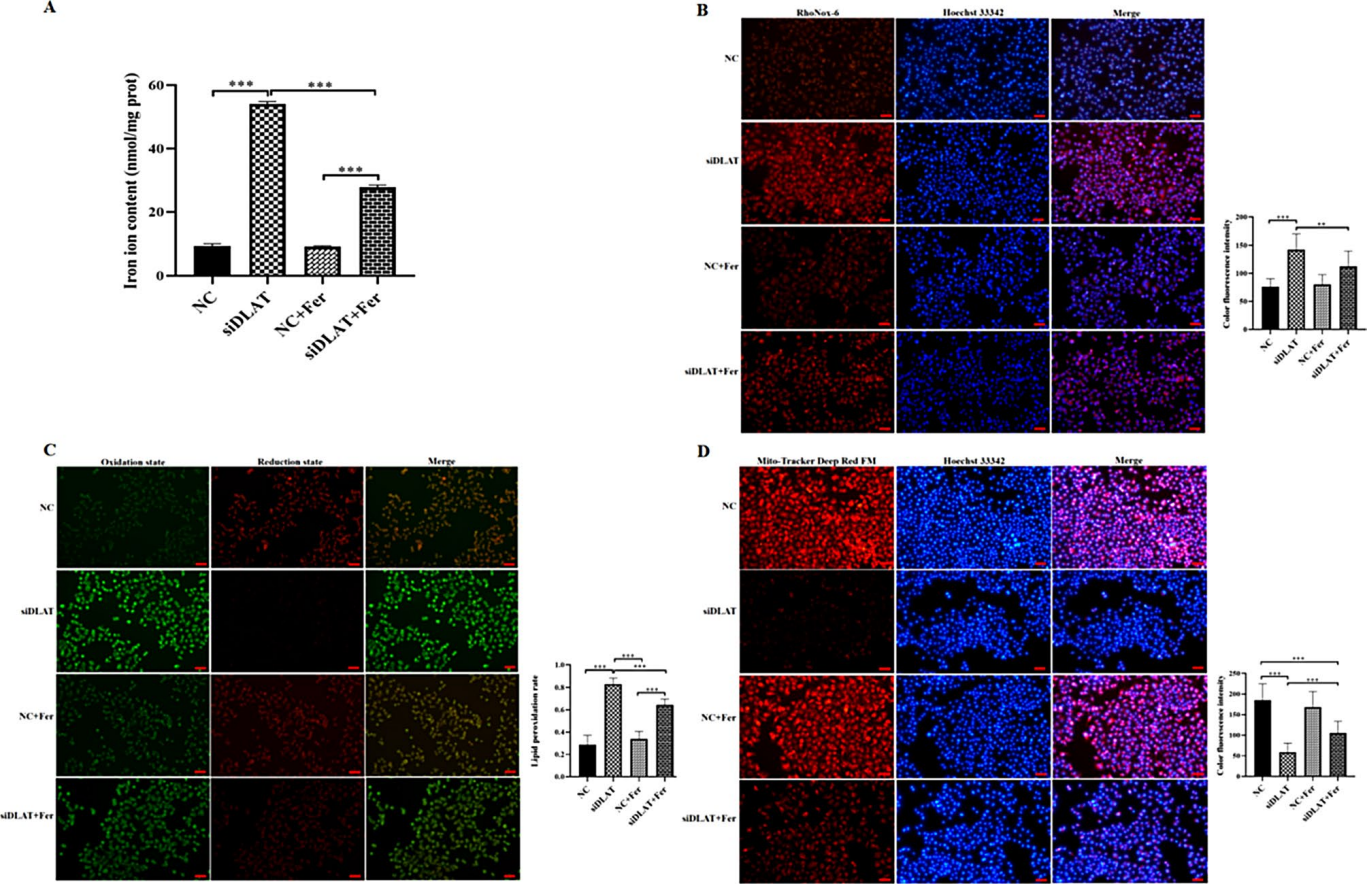
Ferrostatin-1 restored ferroptosis of GC cells caused by knockdown of DLAT. **(A)** The levels of Fe^2+^ in different groups. **(B)** Detection of Fe^2+^ in cells using red fluorescence in different groups. **(C)** Detection of lipid ROS fluorescence in different groups; **(D)** Detection of mitochondrial deep red fluorescence staining in different groups. *: *P* < 0.05, ***: *P* < 0.001

**Fig. 4:**
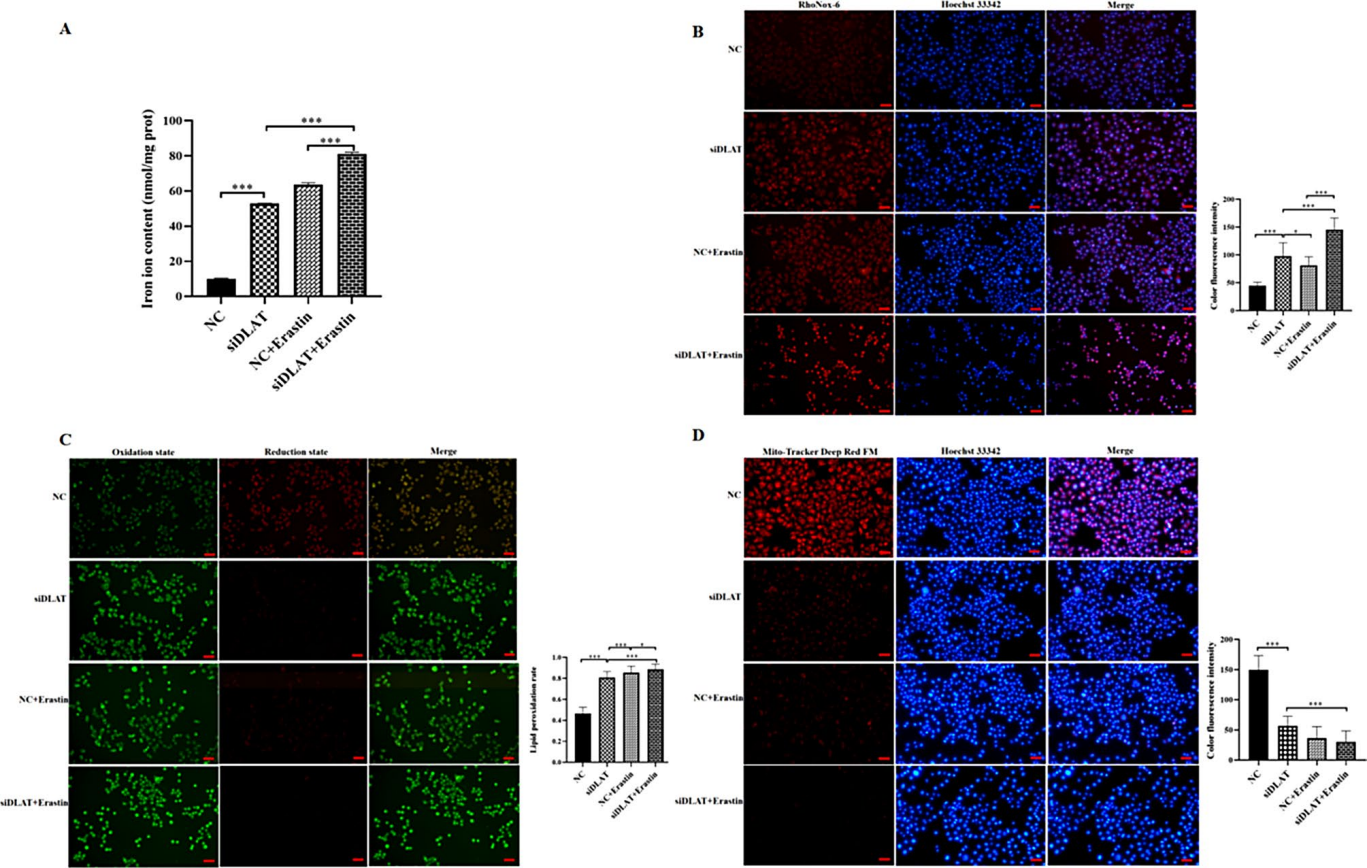
Erastin aggravates ferroptosis in GC cells caused by knockdown of DLAT. **(A)** The levels of Fe^2+^ in different groups. **(B)** Detection of Fe^2+^ in cells using red fluorescence in different groups; **(C)** Detection of lipid ROS fluorescence in different groups; **(D)** Detection of mitochondrial deep red fluorescence staining in different groups. *: *P* < 0.05, ***: *P* < 0.001

Knockdown of DLAT can significantly increase the levels of Fe^2+^ (Fig. 3A–B, *P* < 0.001) and lipid ROS (Fig. 3C, *P* < 0.001) in AGS GC cells, as well as mitochondrial damage (Fig. 3D, *P* < 0.001). After adding ferrostatin-1 to GC cells with si-DLAT, the intracellular Fe^2+^ (*P* < 0.01) and lipid ROS levels (*P* < 0.001), as well as mitochondrial membrane potential (*P* < 0.001), could be restored. However, erastin treatment aggravated ferroptosis in GC cells induced by si-DLAT. The levels of Fe^2+^ (Fig. 4A–B, *P* < 0.001) and lipid ROS (Fig. 4C, *P* < 0.001) in AGS GC cells, as well as mitochondrial membrane potential damage degree (Fig. 4D, *P* < 0.001) all increased significantly compared with the si-DLAT group.

**Fig. 5:**
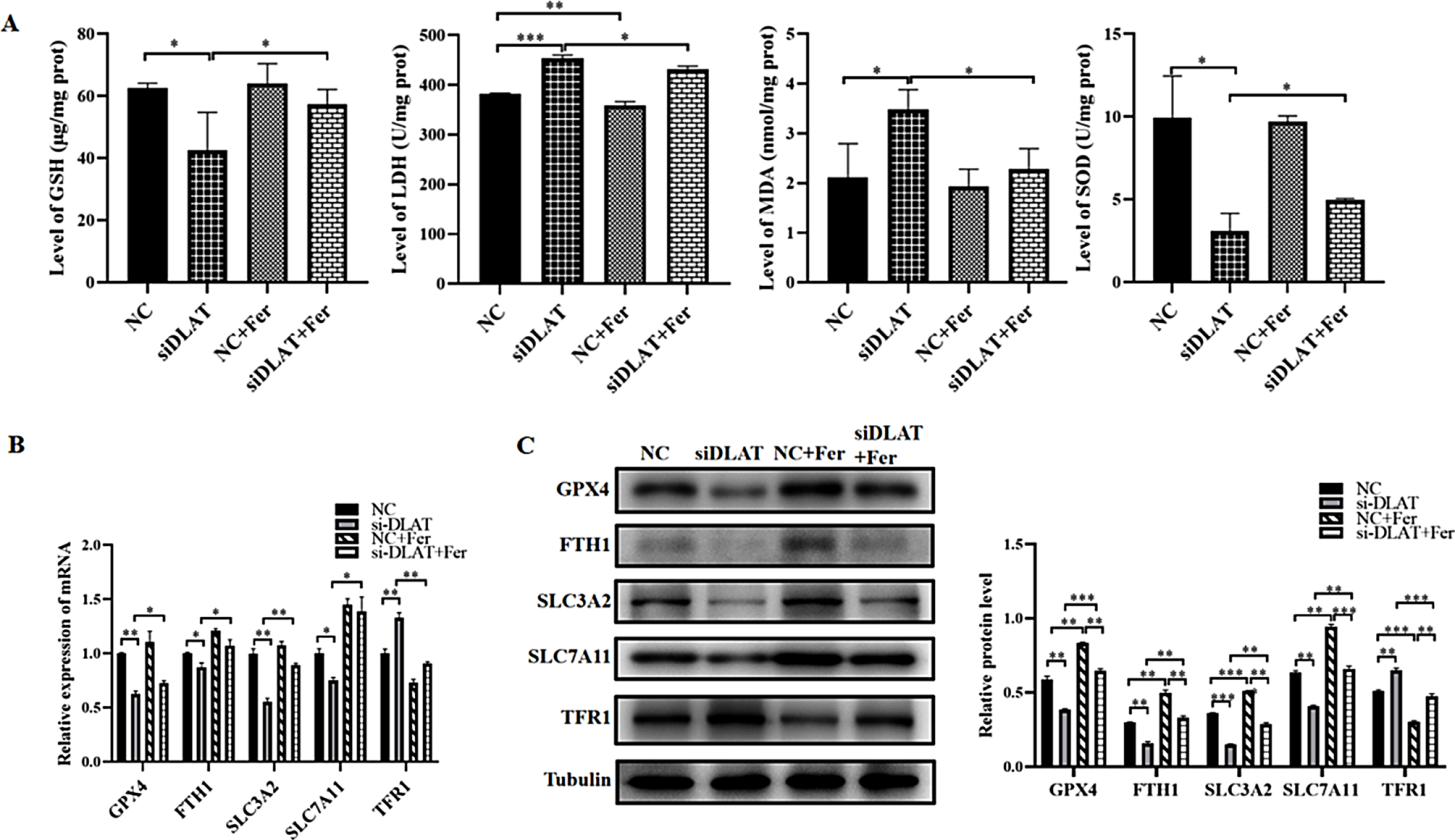
Ferrostatin-1 restores the oxidative stress levels and ferroptosis biomarkers caused by knockdown of DLAT. **(A)** The activity levels of GSH, MDA, SOD and LDH in different groups. **(B)** The mRNA expression levels of GPX4, FTH-1, SLC3A2, SLC7A11 and TFR-1 in different groups. **(C)** The protein expression levels of GPX4, FTH-1, SLC3A2, SLC7A11 and TFR-1 in different groups. *: *P* < 0.05, **:*P* < 0.01, ***: *P* < 0.001

In addition, we found that the levels of GSH (*P* < 0.05) and SOD (*P* < 0.05) were decreased in GC cells after si-DLAT transfection compared with controls, while the expression levels of LDH (*P* < 0.001) and MDA (*P* < 0.05) were increased. After adding ferrostatin-1 to GC cells with si-DLAT, the expression levels of the above indicators could be partially restored (*P* < 0.05, Fig. 5A).

The qRT-PCR and WB experiments demonstrated that DLAT knockdown could significantly reduce the expression of GPX4, FTH1, SLC3A2, and SLC7A11 in AGS GC cells, while increasing the expression level of TFR1. After treatment with ferrostatin-1 in GC cells with si-DLAT, the protein expression levels of the above genes could be partially restored (Fig. 5B–C).

These above results suggested that DLAT inhibited ferroptosis in GC cells.

### DLAT regulates the Nrf2/HO-1/GPX4 pathway to inhibit ferroptosis in GC cells

**Fig. 6:**
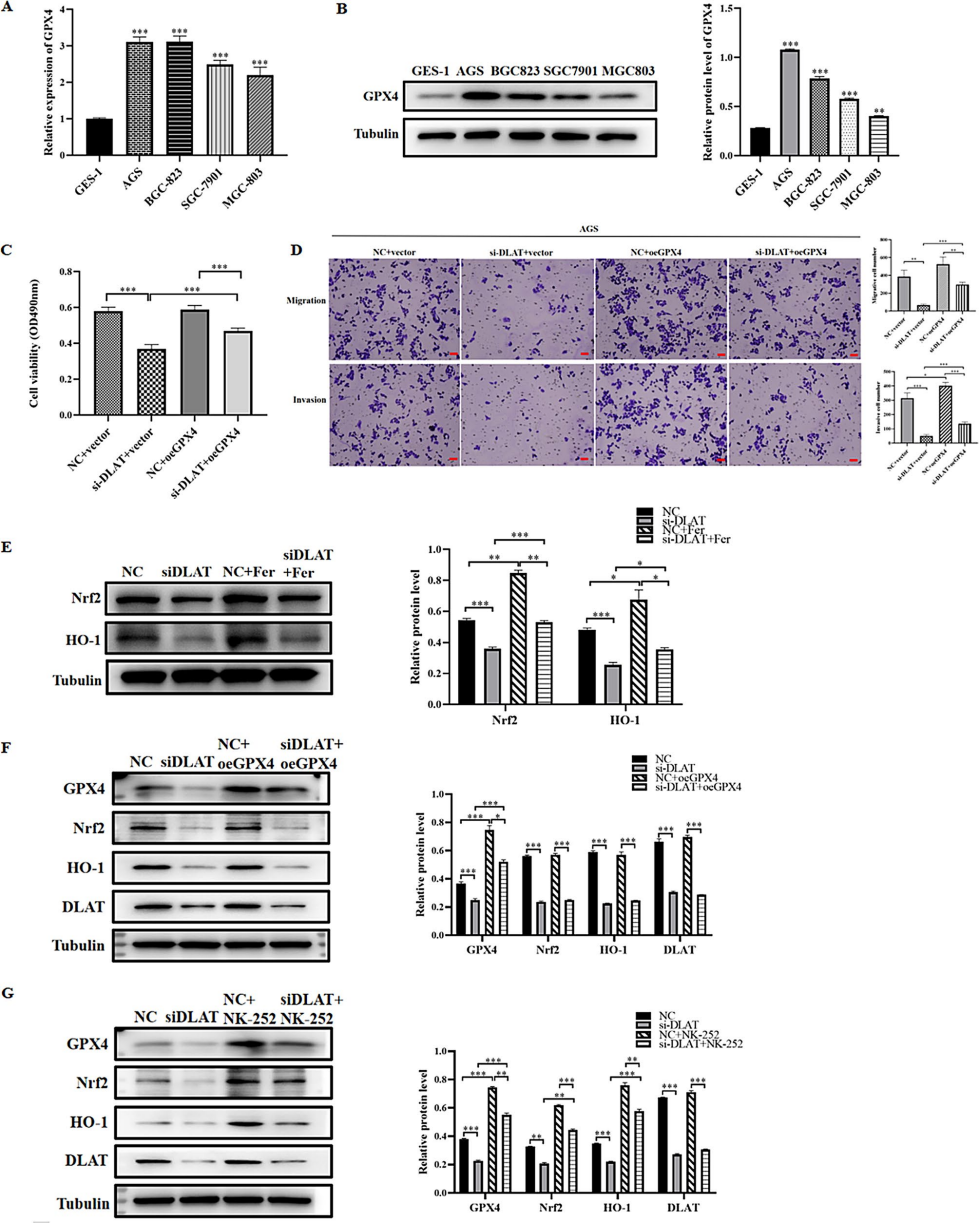
DLAT regulates the Nrf2/HO-1/GPX4 pathway to inhibit ferroptosis in GC cells. **(A)** mRNA expression of GPX4 in each GC cell line. **(B)** Protein expression of GPX4 in each GC cell line. **(C)** MTT assay of cell proliferation in AGS GC cells. **(D)** Transwell invasion and migration assays in AGS GC cells. **(E)** Ferrostatin-1 restored protein expression levels of Nrf2 and HO-1 caused by knockdown of DLAT. **(F)** Protein expression levels of GPX4, Nrf2, HO-1 and DLAT in different groups. **(G)** Protein expression levels of GPX4, Nrf2, HO-1 and DLAT in different groups. *: *P* < 0.05, **:*P* < 0.01, ***: *P* < 0.001

GPX4 mRNA and protein levels were significantly higher in GC cells compared with normal gastric mucosal epithelial cells (Fig. 6A–B, *P* < 0.001). We next explored the regulatory relationship of GPX4 and DLAT using AGS GC cells transfected with siRNAs and a vector overexpressing GPX4 (oe-GPX4). Compared with the control group, si-DLAT significantly reduced the proliferation, migration and invasion abilities of AGS GC cells, while overexpression of GPX4 could partially restore the growth inhibition of GC cells caused by DLAT knockdown (Fig. 6C–D). These results suggested that GPX4 might be involved in the effects of DLAT on the proliferation, migration and invasion of GC cells.

We next carried out remedial experiments to examine whether DLAT-mediated ferroptosis inhibition involves the Nrf2/HO-1/GPX4 pathway. GPX4, Nrf2 and HO-1 were significantly decreased in the si-DLAT group compared with controls (Fig. 6E–F, *P* < 0.001). The expression level of GPX4 partially recovered in the si-DLAT + oe-GPX4 group, however, the expressions of DLAT, Nrf2 and HO-1 remained at a relatively low level, showing no statistically significant difference compared to the control group, indicating that GPX4 was located downstream of DLAT, Nrf2 and HO-1 (Fig. 6F).

Further to investigate whether DLAT promotes the malignant progression of GC by inhibiting ferroptosis through the Nrf2/HO-1/GPX4 axis, we used Nrf2 activators (NK-252, 10 μm) [20]. WB experiments demonstrated that si-DLAT significantly reduced the expressions of DLAT, GPX4, Nrf2 and HO-1, while Nrf2 activators significantly increased the expressions of GPX4, Nrf2 and HO-1, but does not affect the expression of DLAT (Fig. 6G). Nrf2 is an important oxidative stress transcription factor. Compared with the si-DLAT group, adding Nrf2 activators to GC cells with DLAT knockdown can restore the expressions of GPX4, Nrf2, and HO-1, while there was no statistically significant difference in the expression of DLAT (Fig. 6G). Overexpression of the GPX4 gene can significantly reverse the cell growth inhibition phenomenon caused by the knockdown of DLAT. These findings solidified the mechanistic link within this pathway and supported our conclusion that Nrf2 acts upstream of HO-1 and GPX4. Based on these findings, we proposed that DLAT induced ferroptosis in GC cells by regulating the antioxidant mechanism of Nrf2/HO-1/GPX4.

### DLAT influences the proliferation of GC cells through the Nrf2/HO-1 signaling pathway in vivo

**Fig. 7:**
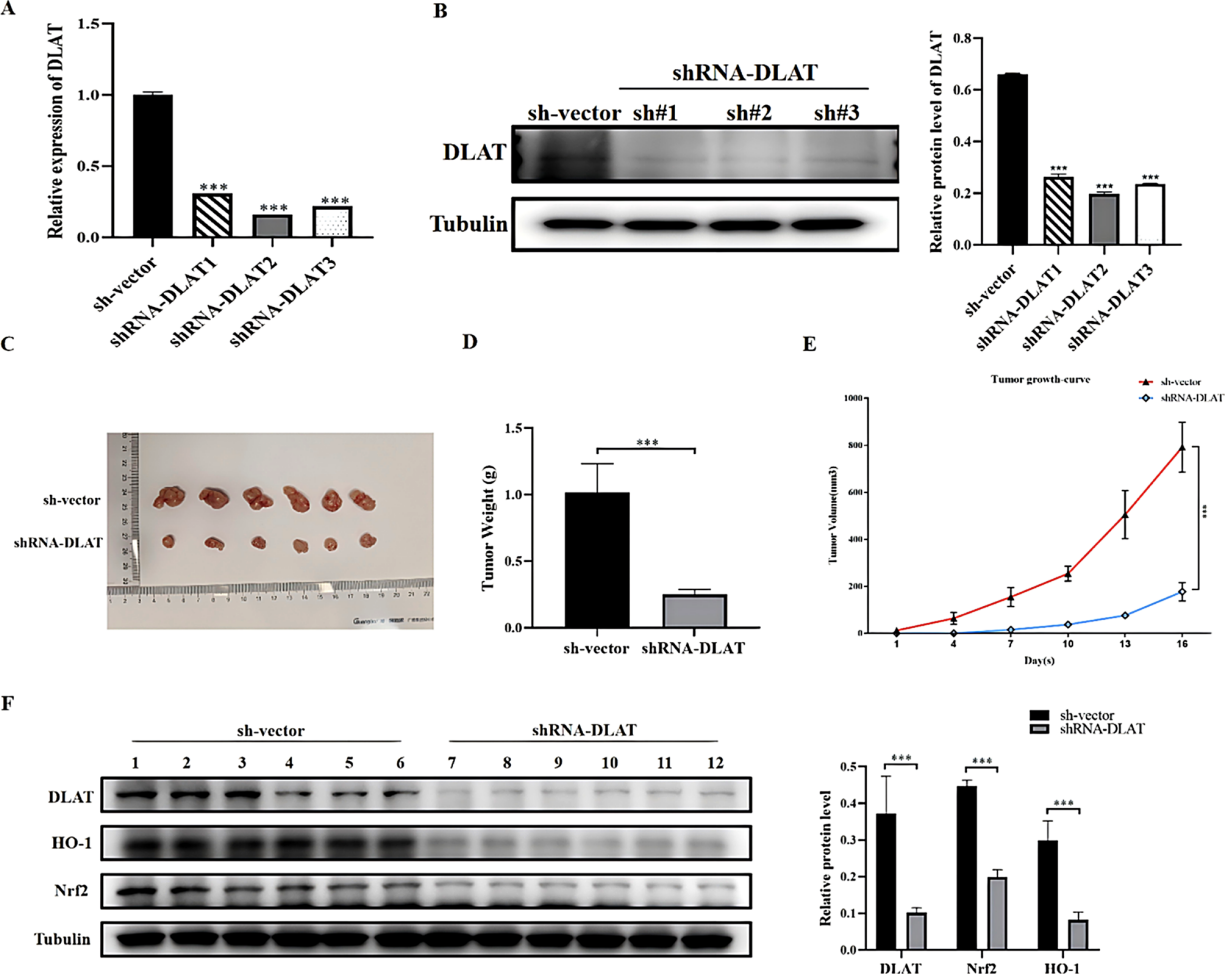
DLAT promotes tumorigenesis of GC *in vivo.***(A)** mRNA expression of DLAT in the stable transfer strain MGC-803 GC cells. **(B)** Protein expression of DLAT in the stable transfer strain MGC-803 GC cells. **(C)** shRNA-DLAT in MGC803 cells formed smaller xenograft tumors than control group. **(D)** Tumor weight. **(E)** Tumor growth curves. **(F)** Protein expression of DLAT, Nrf2 and HO-1 of tumors formed in nude mice injected subcutaneously with shRNA-DLAT or sh-vector in MGC803 cells. ***: *P* < 0.001

We next evaluated whether the results obtained in vitro were also observed *in vivo.* We first established MGC-803 cell lines that stably expressed shRNAs targeting DLAT. As shown in Fig. 7A–B, DLAT mRNA and protein levels were significantly lower in the shRNA-DLAT groups than the control group (*P* < 0.001). The cells expressing shRNA-DLAT-2 showed the most potent DLAT knockdown and were used for subsequent experiments. MGC-803 cells stably expressing shRNA-DLAT or sh-vectors were transplanted into nude mice and tumor growth was monitored for 16 days. The tumor weight and volume of the shRNA-DLAT group were significantly lower than those of the control group (Fig. 7D–E). WB confirmed that DLAT expression in transplanted tumors was significantly reduced in the shRNA-DLAT group compared with the controls (*P* < 0.001, Fig. 7F), verifying the successful construction of the tumor model. The protein expression levels of Nrf2 and HO-1 were significantly decreased in the shRNA-DLAT group compared with the control group (*P* < 0.001, Fig. 7F). These results indicated that DLAT regulated GC tumor growth, and these effects might involve the Nrf2/HO-1 signaling pathway.

## Discussion

Despite the improvements in treatment strategies, the prognosis of GC is poor, in part because of the lack of effective therapeutic targets. Therefore, exploring the molecular mechanism of GC and identifying potential therapeutic targets are important research fields. Xu et al. found that DLAT was abnormally expressed in most malignant tumors [4]. DLAT expression was related to the tumor microenvironment and the infiltration of various immune cells, especially tumor-associated macrophages. Gene set enrichment analysis showed that DLAT was significantly associated with immune-related pathways. Another report showed that DLAT is upregulated in GC tissues and cells, and its high expression is associated with a shortened survival period of patients. Age, gender, histological type, lymph node metastasis and distant metastasis are independent prognostic factors affecting OS of GC. This indicates that DLAT may be a prognostic assessment factor for GC. Further sequencing revealed that the expression of DLAT was positively correlated with the infiltration of Th1 and Th2 immune cells, suggesting that DLAT enrichment is related to immune infiltration and tumor immune escape, providing a potential target for the immunotherapy of GC [21].

Consistent with previous studies [21], we found that the expression of DLAT was elevated in GC tissues and cell lines. Knockdown of DLAT expression inhibited the proliferation, migration, and invasion of GC cells. High DLAT expression was closely related to tumor location, depth of invasion, presence of ulcers, and nerve and vascular invasion, and high DLAT expression was associated with a poor prognosis. Downregulation of DLAT reduced GC cell proliferation, migration, and invasion in vitro and tumorigenicity in vivo.

Ferroptosis is distinct from other known mechanisms of cell death. It is a novel mechanism of cell death caused by the accumulation of iron-dependent lipid peroxidation [6]. During ferroptosis, ROS increases, the mitochondrial volume decreases, and the membrane density increases [22]. Iron metabolism and lipid peroxidation signals are key mediators of ferroptosis. GPX4 is the only GPX member capable of converting phospholipid hydroperoxides into phospholipid alcohols. Recently, researchers found that inhibiting GPX4 promoted ferroptosis [23]. Furthermore, the significant role of ferroptosis in tumor metastasis has been revealed [24, 25].

GPX4 and SLC7A11 are important biomarkers of ferroptosis. The lack of expression of GPX4 and SLC7A11 leads to the production of a large amount of ROS and GSH dysfunction [26]. TFR-1 and FTH-1 are key factors involved in iron metabolism and homeostasis and closely related to ferroptosis. We found that reducing DLAT in GC cells led to the downregulation of GPX4, FTH-1, SLC3A2, and SLC7A11 and increase in TFR-1. The addition of a ferroptosis inhibitor to GC cells transfected with si-DLAT reversed these changes, while adding a ferroptosis inducer aggravated above changes. Furthermore, after knockdown of DLAT expression, LDH activity and MDA content were significantly increased, while GSH and SOD levels significantly decreased. The treatment of GC cells transfected with si-DLAT with the ferroptosis inhibitor also reversed these changes. Compared with the control group, GC cells with DLAT knockdown exhibited significant Fe^2+^ accumulation, elevated lipid ROS levels, and typical ferroptosis morphological features such as the decrease in mitochondrial membrane potential and fluorescence intensity. The ferroptosis specific inhibitor ferrostatin-1 can almost completely reverse these phenotypes. In contrast, when combined with the a ferroptosis inducer erastin, GC cells with DLAT knockdown exhibited the most intense ferroptosis response. These evidences strongly demonstrated that DLAT exerted an inhibitory function on ferroptosis in GC cells, and its absence triggered the ferroptosis pathway, thereby affecting GC cells survival.

We also explored the expression level of GPX4 in GC cell lines, its influence on the biological behavior of GC cells, and its association with DLAT in GC. GPX4 mRNA and protein expression levels were higher in GC cells than in normal gastric mucosal epithelial cells, and overexpression of GPX4 promoted the proliferation, migration, and invasion of GC cells. The remedial experiments indicated that overexpression of GPX4 can specifically salvage the growth inhibition of GC cells caused by DLAT knockdown, but does not affect the expressions of DLAT, Nrf2 and HO-1. This indicated that GPX4 was located downstream of DLAT, Nrf2 and HO-1.

The Nrf2/HO-1 signaling axis was shown to negatively regulate ferroptosis [27]. Nrf2 is a key transcription factor that regulates cellular oxidative stress, reduces the damage of ROS to cells, and functions as an important regulatory factor for maintaining intracellular redox homeostasis [28, 29]. Nrf2 is localized in the cytoplasm under normal physiological conditions. In response to external stimuli, Nrf2 translocates to the nucleus and binds to promoter regions of target genes, activating the downstream molecule HO1 and exerting anti-inflammatory effects. Studies have shown that Nrf2 activation inhibits the production of ROS [30–32]. We further examined whether DLAT inhibits ferroptosis in GC by activating Nrf2/HO-1.

Our research results indicated that knockdown of DLAT induced ferroptosis in GC cells, and inhibited the expression levels of Nrf2 and HO-1 (in *vitro* experiments). In *vivo* experiments showed that the expression levels of Nrf2 and HO-1 were decreased in tumors derived from GC cells expressing si-DLAT. Therefore, we speculated that DLAT inhibited ferroptosis by activating Nrf2/HO-1, thereby reducing the generation of ROS.

Remedial experiments indicated that overexpression of GPX4 can reverse the cell growth inhibition caused by the knockdown of DLAT, but it did not affect the expression levels of DLAT, Nrf2, and HO-1. In order to further clarify the role of the Nrf2/HO-1/GPX4 axis in the regulation of ferroptosis by DLAT during the malignant progression of GC, we used Nrf2 activators. The results indicated that Nrf2 activators significantly increased the expressions of GPX4, Nrf2 and HO-1, but did not affect the expression of DLAT. When Nrf2 activators were added to GC cells that had already expressed low levels of DLAT, it could restore the expression levels of GPX4, Nrf2 and HO-1, but did not affect the expression of DLAT. These evidences all suggested that DLAT induced ferroptosis in GC cells by regulating the antioxidant mechanism of Nrf2/HO-1/GPX4.

This study has several limitations. First, our study was a single-center study, and the clinical sample size was relatively small. A larger sample multi-center studies and independent datasets from public databases are needed to further explore the correlation between DLAT and the clinicopathological characteristics of GC. Second, we only evaluated tumor growth and the protein expression of DLAT and related signaling pathways in tumors. We did not investigate the impact of DLAT on ferroptosis and the invasion and metastasis of GC in vivo. We plan to investigate these topics in a future study.

In summary, our results show that DLAT expression is associated with tumor location, depth of invasion, presence of ulcers, and nerve and vascular invasion in GC. High expression of DLAT indicates a poor prognosis for patients with GC. DLAT promotes malignant progression of GC by regulating ferroptosis and activating the Nrf2/HO-1/GPX4 signaling pathway. Thus, DLAT may be a potential therapeutic target for GC.

## Supplementary Information

Below is the link to the electronic supplementary material.

Supplementary Material 1

Supplementary Material 2

## Data Availability

No datasets were generated or analysed during the current study.
