# Supplementary material for: DLAT inhibits ferroptosis to promote malignant progression of gastric cancer through Nrf2/HO-1/GPX4 signaling pathway
Source: Biol Direct. 2026 Apr 2;21:64. doi: 10.1186/s13062-026-00767-7 (PMC13162497; doi:10.1186/s13062-026-00767-7)
Supplement: Supplementary file 1 — Supplementary Material 1 [file 13062_2026_767_MOESM1_ESM.zip › Cell line authentication/AGS Cell line authentication/AGS database comparison.docx]

| **EV** | **Cell No.** | **Cell name** | **Locus names** | | | | | | | | |
| --- | --- | --- | --- | --- | --- | --- | --- | --- | --- | --- | --- |
|  |  |  | **D5S818** | **D13S317** | **D7S820** | **D16S539** | **VWA** | **TH01** | **AM** | **TPOX** | **CSF1PO** |
|  | ***Query (Your Cell)*** | | **9,12** | **12,12** | **10,11** | **11,13** | **16,17** | **6,7** | **X,X** | **11,12** | **11,12** |

| 1.0(36/36) | **CRL-1739** | **AGS** | **['9', '12']** | **['12', '12']** | **['10', '11']** | **['11', '13']** | **['16', '17']** | **['6', '7']** | **['X', 'X']** | **['11', '12']** | **['11', '12']** |
| --- | --- | --- | --- | --- | --- | --- | --- | --- | --- | --- | --- |

| 1.0(36/36) | **CVCL_0139Worst** | **AGS** | **['9', '12']** | **['12', '12']** | **['10', '11']** | **['11', '13']** | **['16', '17']** | **['6', '7']** | **['X', 'X']** | **['11', '12']** | **['11', '12']** |
| --- | --- | --- | --- | --- | --- | --- | --- | --- | --- | --- | --- |

| 1.0(36/36) | **CVCL_0139Best** | **AGS** | **['9', '12']** | **['12', '12']** | **['10', '11']** | **['11', '13']** | **['16', '17']** | **['6', '7']** | **['X', 'X']** | **['11', '12']** | **['11', '12']** |
| --- | --- | --- | --- | --- | --- | --- | --- | --- | --- | --- | --- |

| 0.91(33/36) | **CVCL_0139 Best** | **AGS** | **['9', '12']** | **['12', '12']** | **['10', '11']** | **['11', '13']** | **['16', '17']** | **['6', '7']** | **['']** | **['11', '12']** | **['11', '12']** |
| --- | --- | --- | --- | --- | --- | --- | --- | --- | --- | --- | --- |

| 0.91(33/36) | **CVCL_A4BR** | **AGS-Luc2** | **['9', '12']** | **['12', '12']** | **['10', '11']** | **['11', '13']** | **['16', '17']** | **['6', '7']** | **['']** | **['11', '12']** | **['11', '12']** |
| --- | --- | --- | --- | --- | --- | --- | --- | --- | --- | --- | --- |

| 0.91(33/36) | **CVCL_0139 Worst** | **AGS** | **['9', '12']** | **['12', '12']** | **['10', '11']** | **['11', '13']** | **['16', '17']** | **['6', '7']** | **['']** | **['11', '12']** | **['11', '12']** |
| --- | --- | --- | --- | --- | --- | --- | --- | --- | --- | --- | --- |

| 0.72(26/36) | **JCRB1093** | **PSVK1** | **['12', '13']** | **['12', '12']** | **['10', '11']** | **['9', '13']** | **['16', '16']** | **['6', '7']** | **['X', 'Y']** | **['8', '11']** | **['11', '12']** |
| --- | --- | --- | --- | --- | --- | --- | --- | --- | --- | --- | --- |

| 0.72(26/36) | **CRL-2466** | **CCD-1118SK** | **['12', '13']** | **['11', '12']** | **['9', '10']** | **['11', '13']** | **['16', '17']** | **['6', '9']** | **['X', 'X']** | **['8', '11']** | **['11', '12']** |
| --- | --- | --- | --- | --- | --- | --- | --- | --- | --- | --- | --- |

| 0.72(26/36) | **CRL-2614** | **Ect1/E6E7** | **['11', '12']** | **['12', '12']** | **['10', '10']** | **['10', '13']** | **['16', '17']** | **['6', '10']** | **['X', 'X']** | **['8', '11']** | **['11', '12']** |
| --- | --- | --- | --- | --- | --- | --- | --- | --- | --- | --- | --- |

| 0.72(26/36) | **RCB1724** | **JHSK-rec** | **['11', '12']** | **['9', '9']** | **['10', '11']** | **['11', '13']** | **['14', '16']** | **['6', '7']** | **['X', 'X']** | **['8', '12']** | **['11', '12']** |
| --- | --- | --- | --- | --- | --- | --- | --- | --- | --- | --- | --- |

| 0.72(26/36) | **RCB1549** | **YSCCC** | **['10', '12']** | **['12', '12']** | **['10', '11']** | **['10', '11']** | **['16', '17']** | **['7', '9']** | **['X', 'X']** | **['11', '11']** | **['10', '11']** |
| --- | --- | --- | --- | --- | --- | --- | --- | --- | --- | --- | --- |

| 0.72(26/36) | **CVCL_A475** | **PER-377** | **['12', '12']** | **['12', '12']** | **['11', '12']** | **['11', '11']** | **['14', '17']** | **['6', '9.3']** | **['X', 'X']** | **['11', '12']** | **['11', '12']** |
| --- | --- | --- | --- | --- | --- | --- | --- | --- | --- | --- | --- |

| 0.72(26/36) | **CVCL_A975** | **chHES-60** | **['9', '10']** | **['8', '12']** | **['11', '12']** | **['11', '13']** | **['17', '18']** | **['6', '7']** | **['X', 'X']** | **['8', '11']** | **['11', '12']** |
| --- | --- | --- | --- | --- | --- | --- | --- | --- | --- | --- | --- |

| 0.72(26/36) | **CVCL_6786** | **WM1232** | **['10', '12']** | **['12', '12']** | **['11', '12']** | **['11', '12']** | **['16', '16']** | **['7', '9.3']** | **['X', 'X']** | **['11', '12']** | **['11', '12']** |
| --- | --- | --- | --- | --- | --- | --- | --- | --- | --- | --- | --- |

| 0.72(26/36) | **CVCL_1232Worst** | **GI-ME-N** | **['12', '12']** | **['12', '12']** | **['10', '11']** | **['9', '12']** | **['16', '19']** | **['6', '7']** | **['X', 'X']** | **['11', '11']** | **['11', '12']** |
| --- | --- | --- | --- | --- | --- | --- | --- | --- | --- | --- | --- |

| 0.72(26/36) | **CVCL_8795** | **OCI-Ly10** | **['10', '12']** | **['11', '12']** | **['10', '11']** | **['13', '13']** | **['16', '17']** | **['6', '7']** | **['X', 'X']** | **['8', '8']** | **['11', '12']** |
| --- | --- | --- | --- | --- | --- | --- | --- | --- | --- | --- | --- |

| 0.7(25/36) | **426** | **SW-1710** | **['12', '12']** | **['12', '12']** | **['8', '11']** | **['8', '11']** | **['16', '17']** | **['7', '9.3', '8']** | **['X', 'X']** | **['9', '11']** | **['11', '12']** |
| --- | --- | --- | --- | --- | --- | --- | --- | --- | --- | --- | --- |

| 0.7(25/36) | **CVCL_1721Worst** | **SW1710** | **['12', '12']** | **['12', '12']** | **['8', '11']** | **['8', '11']** | **['16', '17']** | **['7', '8', '9.3']** | **['X', 'X']** | **['9', '11']** | **['11', '12']** |
| --- | --- | --- | --- | --- | --- | --- | --- | --- | --- | --- | --- |

| 0.7(25/36) | **CVCL_1721Best** | **SW1710** | **['12', '12']** | **['12', '12']** | **['8', '11']** | **['8', '11']** | **['16', '17']** | **['7', '8', '9.3']** | **['X', 'X']** | **['9', '11']** | **['11', '12']** |
| --- | --- | --- | --- | --- | --- | --- | --- | --- | --- | --- | --- |

| 0.69(25/36) | **CVCL_8403** | **PSVK1** | **['12', '13']** | **['12', '12']** | **['10', '11']** | **['9', '13']** | **['16', '16']** | **['6', '7']** | **['']** | **['8', '11']** | **['11', '12']** |
| --- | --- | --- | --- | --- | --- | --- | --- | --- | --- | --- | --- |

| 0.67(24/36) | **JCRB0435** | **JHH-4** | **['9', '13']** | **['12', '12']** | **['10', '12']** | **['12', '13']** | **['14', '17']** | **['6', '9']** | **['X', 'X']** | **['8', '11']** | **['11', '12']** |
| --- | --- | --- | --- | --- | --- | --- | --- | --- | --- | --- | --- |

| 0.67(24/36) | **654** | **GI-ME-N** | **['12', '12']** | **['8', '12']** | **['10', '11']** | **['9', '12']** | **['16', '19']** | **['6', '7']** | **['X', 'X']** | **['11', '11']** | **['11', '12']** |
| --- | --- | --- | --- | --- | --- | --- | --- | --- | --- | --- | --- |

| 0.67(24/36) | **CRL-1622** | **KLE** | **['9', '12']** | **['12', '12']** | **['11', '12']** | **['11', '12']** | **['16', '16']** | **['6', '7']** | **['X', 'X']** | **['8', '11']** | **['13', '14']** |
| --- | --- | --- | --- | --- | --- | --- | --- | --- | --- | --- | --- |

| 0.67(24/36) | **CRL-7671** | **Hs 919.Sk** | **['12', '12']** | **['11', '12']** | **['10', '10']** | **['11', '13']** | **['16', '17']** | **['6', '9.3']** | **['X', 'X']** | **['8', '11']** | **['10', '12']** |
| --- | --- | --- | --- | --- | --- | --- | --- | --- | --- | --- | --- |

| 0.67(24/36) | **CRL-7672** | **Hs 919.T** | **['12', '12']** | **['11', '12']** | **['10', '10']** | **['11', '13']** | **['16', '17']** | **['6', '9.3']** | **['X', 'X']** | **['8', '11']** | **['10', '12']** |
| --- | --- | --- | --- | --- | --- | --- | --- | --- | --- | --- | --- |

| 0.67(24/36) | **JCRB0106** | **SCCH-26** | **['11', '12']** | **['11', '12']** | **['10', '10']** | **['11', '12']** | **['16', '16']** | **['6', '7']** | **['X', 'X']** | **['8', '11']** | **['11', '12']** |
| --- | --- | --- | --- | --- | --- | --- | --- | --- | --- | --- | --- |

| 0.67(24/36) | **RCB1952** | **SCCH-26** | **['11', '12']** | **['11', '12']** | **['10', '10']** | **['11', '12']** | **['16', '16']** | **['6', '7']** | **['X', 'X']** | **['8', '11']** | **['11', '12']** |
| --- | --- | --- | --- | --- | --- | --- | --- | --- | --- | --- | --- |

| 0.67(24/36) | **CRL-2615** | **End1/E6E7** | **['11', '12']** | **['9', '12']** | **['10', '10']** | **['10', '13']** | **['16', '17']** | **['6', '6']** | **['X', 'X']** | **['8', '11']** | **['11', '12']** |
| --- | --- | --- | --- | --- | --- | --- | --- | --- | --- | --- | --- |

| 0.67(24/36) | **CRL-5946** | **NCI-H2452 [H2452]** | **['11', '12']** | **['12', '12']** | **['9', '11']** | **['11', '13']** | **['17', '18']** | **['6', '9.3']** | **['X', 'Y']** | **['8', '11']** | **['11', '12']** |
| --- | --- | --- | --- | --- | --- | --- | --- | --- | --- | --- | --- |

| 0.67(24/36) | **CRL-7828** | **Hs 67.Th** | **['12', '12']** | **['11', '12']** | **['8', '12']** | **['11', '13']** | **['16', '17']** | **['6', '9.3']** | **['X', 'X']** | **['8', '12']** | **['11', '12']** |
| --- | --- | --- | --- | --- | --- | --- | --- | --- | --- | --- | --- |

| 0.67(24/36) | **HTB-163** | **Hs 67** | **['12', '12']** | **['11', '12']** | **['8', '12']** | **['11', '13']** | **['16', '17']** | **['6', '9.3']** | **['X', 'X']** | **['8', '12']** | **['11', '12']** |
| --- | --- | --- | --- | --- | --- | --- | --- | --- | --- | --- | --- |

| 0.67(24/36) | **JCRB1194** | **PL508** | **['12', '13']** | **['9', '12']** | **['10', '11']** | **['11', '13']** | **['14', '17']** | **['7', '9']** | **['X', 'X']** | **['11', '11']** | **['12', '13']** |
| --- | --- | --- | --- | --- | --- | --- | --- | --- | --- | --- | --- |

| 0.67(24/36) | **CCL-76** | **citrullinemia** | **['9', '12']** | **['10', '12']** | **['8', '11']** | **['11', '12']** | **['17', '20']** | **['7', '9.3']** | **['X', 'X']** | **['8', '11']** | **['11', '12']** |
| --- | --- | --- | --- | --- | --- | --- | --- | --- | --- | --- | --- |

| 0.67(24/36) | **CRL-2846** | **CHON-001** | **['11', '12']** | **['12', '14']** | **['12', '12']** | **['11', '13']** | **['16', '17']** | **['6', '7']** | **['X', 'X']** | **['8', '11']** | **['10', '11']** |
| --- | --- | --- | --- | --- | --- | --- | --- | --- | --- | --- | --- |

| 0.67(24/36) | **CRL-2847** | **CHON-002** | **['11', '12']** | **['12', '14']** | **['12', '12']** | **['11', '13']** | **['16', '17']** | **['6', '7']** | **['X', 'X']** | **['8', '11']** | **['10', '11']** |
| --- | --- | --- | --- | --- | --- | --- | --- | --- | --- | --- | --- |

| 0.67(24/36) | **CRL-2848** | **CHON-003** | **['11', '12']** | **['12', '14']** | **['12', '12']** | **['11', '13']** | **['16', '17']** | **['6', '7']** | **['X', 'X']** | **['8', '11']** | **['10', '11']** |
| --- | --- | --- | --- | --- | --- | --- | --- | --- | --- | --- | --- |

| 0.67(24/36) | **CRL-2856** | **CHON-004** | **['11', '12']** | **['12', '14']** | **['12', '12']** | **['11', '13']** | **['16', '17']** | **['6', '7']** | **['X', 'X']** | **['8', '11']** | **['10', '11']** |
| --- | --- | --- | --- | --- | --- | --- | --- | --- | --- | --- | --- |

| 0.67(24/36) | **CRL-2857** | **CHON-005** | **['11', '12']** | **['12', '14']** | **['12', '12']** | **['11', '13']** | **['16', '17']** | **['6', '7']** | **['X', 'X']** | **['8', '11']** | **['10', '11']** |
| --- | --- | --- | --- | --- | --- | --- | --- | --- | --- | --- | --- |

| 0.67(24/36) | **CRL-2914** | **M4A4** | **['11', '12']** | **['12', '12']** | **['8', '10']** | **['13', '13']** | **['16', '18']** | **['6', '7']** | **['X', 'X']** | **['8', '11']** | **['11', '11']** |
| --- | --- | --- | --- | --- | --- | --- | --- | --- | --- | --- | --- |

| 0.67(24/36) | **CRL-2915** | **M4A4 GFP** | **['11', '12']** | **['12', '12']** | **['8', '10']** | **['13', '13']** | **['16', '18']** | **['6', '7']** | **['X', 'X']** | **['8', '11']** | **['11', '11']** |
| --- | --- | --- | --- | --- | --- | --- | --- | --- | --- | --- | --- |
