# Supplementary material for: DLAT inhibits ferroptosis to promote malignant progression of gastric cancer through Nrf2/HO-1/GPX4 signaling pathway
Source: Biol Direct. 2026 Apr 2;21:64. doi: 10.1186/s13062-026-00767-7 (PMC13162497; doi:10.1186/s13062-026-00767-7)
Supplement: Supplementary file 1 — Supplementary Material 1 [file 13062_2026_767_MOESM1_ESM.zip › Cell line authentication/MGC-803 Cell line authentication/MGC-803.html]

cgi proc


  

|  |
| --- |
| **Result of STR matching analysis by your data.  **- DSMZ Profile Database -**** |

  

**A graphical presentation is shown at the bottom of this page.**

| EV | Cell No. | Cell name | Locus names | | | | | | | | | Figures |
| --- | --- | --- | --- | --- | --- | --- | --- | --- | --- | --- | --- | --- |
|  |  |  | D5S818 | D13S317 | D7S820 | D16S539 | VWA | TH01 | AM | TPOX | CSF1PO |  |
|  | *Query (Your Cell)* | | *10,11,12* | *7,13.3* | *11,12* | *9,11* | *16,17,18* | *7,9* | *X,X* | *12,12* | *9,10,12* |  |
| 0.72(28/39) | JCRB0649.1 | HeLa.P3 | **11**,**12** | 12,**13.3** | 8,**12** | **9**,10 | **16**,**18** | **7**,7 | **X**,**X** | **12**,**12** | **9**,**10** | - |
| 0.70(28/40) | JCRB9066 | Chang Liver | **11**,**12** | 12,**13.3** | 8,**12** | **9**,10 | **16**,**17**,**18** | **7**,7 | **X**,**X** | 8,**12** | **9**,**10** | - |
| 0.67(26/39) | 57 | HELA | **11**,**12** | 12,**13.3** | 8,**12** | **9**,10 | **16**,**18** | **7**,7 | **X**,**X** | 8,**12** | **9**,**10** | - |
| 0.67(26/39) | 57 | HELA | **11**,**12** | 12,**13.3** | 8,**12** | **9**,10 | **16**,**18** | **7**,7 | **X**,**X** | 8,**12** | **9**,**10** | - |
| 0.67(26/39) | 116 | GIRARDI HEART C2 | **11**,**12** | 12,**13.3** | 8,**12** | **9**,10 | **16**,**18** | **7**,7 | **X**,**X** | 8,**12** | **9**,**10** | - |
| 0.67(26/39) | 121 | GIRARDI HEART C7 | **11**,**12** | 12,**13.3** | 8,**12** | **9**,10 | **16**,**18** | **7**,7 | **X**,**X** | 8,**12** | **9**,**10** | - |
| 0.67(26/39) | 136 | KB | **11**,**12** | 12,**13.3** | 8,**12** | **9**,10 | **16**,**18** | **7**,7 | **X**,**X** | 8,**12** | **9**,**10** | - |
| 0.67(26/39) | 149 | KB-V1 | **11**,**12** | 12,**13.3** | 8,**12** | **9**,10 | **16**,**18** | **7**,7 | **X**,**X** | 8,**12** | **9**,**10** | - |
| 0.67(26/39) | 158 | KB-3-1 | **11**,**12** | 12,**13.3** | 8,**12** | **9**,10 | **16**,**18** | **7**,7 | **X**,**X** | 8,**12** | **9**,**10** | - |
| 0.67(26/39) | 161 | HELA-S3 | **11**,**12** | 12,**13.3** | 8,**12** | **9**,10 | **16**,**18** | **7**,7 | **X**,**X** | 8,**12** | **9**,**10** | - |
| 0.67(26/39) | 227 | BT-B | **11**,**12** | **13.3**,13.3 | 8,**12** | **9**,10 | **16**,**18** | **7**,7 | **X**,**X** | 8,**12** | **9**,**10** | - |
| 0.67(26/39) | 228 | SBC-2 | **11**,**12** | 12,**13.3** | **12**,12 | **9**,10 | **16**,**18** | **7**,7 | **X**,**X** | 8,**12** | **9**,**10** | - |
| 0.67(26/39) | 229 | SBC-7 | **11**,**12** | 12,**13.3** | **12**,12 | **9**,10 | **16**,**18** | **7**,7 | **X**,**X** | 8,**12** | **9**,**10** | - |
| 0.67(26/39) | CCL-17 | KB | **11**,**12** | 12,**13.3** | 8,**12** | **9**,10 | **16**,**18** | **7**,7 | **X**,**X** | 8,**12** | **9**,**10** | - |
| 0.67(26/39) | CCL-2 | HeLa | **11**,**12** | 12,**13.3** | 8,**12** | **9**,10 | **16**,**18** | **7**,7 | **X**,**X** | 8,**12** | **9**,**10** | - |
| 0.67(26/39) | CCL-2.1 | HeLa 229 | **11**,**12** | 12,**13.3** | 8,**12** | **9**,10 | **16**,**18** | **7**,7 | **X**,**X** | 8,**12** | **9**,**10** | - |
| 0.67(26/39) | CCL-2.2 | HeLa S3 | **11**,**12** | **13.3**,13.3 | 8,**12** | **9**,10 | **16**,**18** | **7**,7 | **X**,**X** | 8,**12** | **9**,**10** | - |
| 0.67(26/39) | CCL-20.2 | clone 1-5c-4 [Wong-Kilbourne derivative (D) of Chang conjunctiva] | **11**,**12** | 12,**13.3** | 8,**12** | **9**,10 | **16**,**18** | **7**,7 | **X**,**X** | 8,**12** | **9**,**10** | - |
| 0.67(26/39) | CCL-21 | AV3 | **11**,**12** | **13.3**,13.3 | **12**,12 | **9**,10 | **16**,**18** | **7**,7 | **X**,**X** | 8,**12** | **9**,**10** | - |
| 0.67(26/39) | CCL-23 | HEp-2 | **11**,**12** | 12,**13.3** | 8,**12** | **9**,10 | **16**,**18** | **7**,7 | **X**,**X** | 8,**12** | **9**,**10** | - |
| 0.67(26/39) | CCL-25 | WISH | **11**,**12** | **13.3**,13.3 | 8,**12** | **9**,10 | **16**,**18** | **7**,7 | **X**,**X** | 8,**12** | **9**,**10** | - |
| 0.67(26/39) | CCL-5 | L-132 | **11**,**12** | 12,**13.3** | 8,**12** | **9**,10 | **16**,**18** | **7**,7 | **X**,**X** | 8,**12** | **9**,**10** | - |
| 0.67(26/39) | CCL-6 | intestine 407 | **11**,**12** | 12,**13.3** | 8,**12** | **9**,10 | **16**,**18** | **7**,7 | **X**,**X** | 8,**12** | **9**,**10** | - |
| 0.67(26/39) | CCL-62 | FL | **11**,**12** | 12,**13.3** | 8,**12** | **9**,10 | **16**,**18** | **7**,7 | **X**,**X** | 8,**12** | **9**,**10** | - |
| 0.67(26/39) | CL-48 | WRL 68 | **11**,**12** | 12,**13.3** | 8,**12** | **9**,10 | **16**,**18** | **7**,7 | **X**,**X** | 8,**12** | **9**,**10** | - |
| 0.67(26/39) | CRL-12510 | HeLa NRI | **11**,**12** | 12,**13.3** | 8,**12** | **9**,10 | **16**,**18** | **7**,7 | **X**,**X** | 8,**12** | **9**,**10** | - |
| 0.67(26/39) | CRL-13002 | GH329 | **11**,**12** | 12,**13.3** | 8,**12** | **9**,10 | **16**,**18** | **7**,7 | **X**,**X** | 8,**12** | **9**,**10** | - |
| 0.67(26/39) | CRL-13003 | GH354 | **11**,**12** | **13.3**,13.3 | 8,**12** | **9**,10 | **16**,**18** | **7**,7 | **X**,**X** | 8,**12** | **9**,**10** | - |
| 0.67(26/39) | CRL-13011 | HeLaNR1 | **11**,**12** | 12,**13.3** | 8,**12** | **9**,10 | **16**,**18** | **7**,7 | **X**,**X** | 8,**12** | **9**,**10** | - |
| 0.67(26/39) | CRL-1958 | H1HeLa | **11**,**12** | 12,**13.3** | 8,**12** | **9**,10 | **16**,**18** | **7**,7 | **X**,**X** | 8,**12** | **9**,**10** | - |
| 0.67(26/39) | CRL-2972 | HeLaRC32 [HeRC32] | **11**,**12** | 12,**13.3** | 8,**12** | **9**,10 | **16**,**18** | **7**,7 | **X**,**X** | 8,**12** | **9**,**10** | - |
| 0.67(26/39) | CRM-CCL-2 | HeLa | **11**,**12** | 12,**13.3** | 8,**12** | **9**,10 | **16**,**18** | **7**,7 | **X**,**X** | 8,**12** | **9**,**10** | - |
| 0.67(26/39) | IFO50004 | WISH | **11**,**12** | **13.3**,13.3 | 8,**12** | **9**,10 | **16**,**18** | **7**,7 | **X**,**X** | 8,**12** | **9**,**10** | - |
| 0.67(26/39) | IFO50005 | J-111 | **11**,**12** | 12,**13.3** | 8,**12** | **9**,10 | **16**,**18** | **7**,7 | **X**,**X** | 8,**12** | **9**,**10** | - |
| 0.67(26/39) | IFO50016 | Chang Liver | **11**,**12** | 12,**13.3** | 8,**12** | **9**,10 | **16**,**18** | **7**,7 | **X**,**X** | 8,**12** | **9**,**10** | - |
| 0.67(26/39) | JCRB0073 | J-111 | **11**,**12** | 12,**13.3** | 8,**12** | **9**,10 | **16**,**18** | **7**,7 | **X**,**X** | 8,**12** | **9**,**10** | - |
| 0.67(26/39) | JCRB0213 | HeLa AG | **11**,**12** | **13.3**,13.3 | 8,8 | **9**,10 | **16**,**18** | **7**,7 | **X**,**X** | **12**,**12** | **9**,**10** | - |
| 0.67(26/39) | JCRB0214 | HeLa TG | **11**,**12** | **13.3**,13.3 | 8,8 | **9**,10 | **16**,**18** | **7**,7 | **X**,**X** | **12**,**12** | **9**,**10** | - |
| 0.67(26/39) | JCRB0215 | HeLa TG CAP | **11**,**12** | **13.3**,13.3 | 8,8 | **9**,10 | **16**,**18** | **7**,7 | **X**,**X** | **12**,**12** | **9**,**10** | - |
| 0.67(26/39) | JCRB0649 | HeLa.P3 | **11**,**12** | 12,**13.3** | 8,**12** | **9**,10 | **16**,**18** | **7**,7 | **X**,**X** | 8,**12** | **9**,**10** | - |
| 0.67(26/39) | JCRB1318 | HeLa9903 | **11**,**12** | 12,**13.3** | 8,**12** | **9**,10 | **16**,**18** | **7**,7 | **X**,**X** | 8,**12** | **9**,**10** | - |
| 0.67(26/39) | JCRB9004 | HeLa | **11**,**12** | 12,**13.3** | 8,**12** | **9**,10 | **16**,**18** | **7**,7 | **X**,**X** | 8,**12** | **9**,**10** | - |
| 0.67(26/39) | JCRB9027 | KB | **11**,**12** | 12,**13.3** | 8,**12** | **9**,10 | **16**,**18** | **7**,7 | **X**,**X** | 8,**12** | **9**,**10** | - |
| 0.67(26/39) | JCRB9086 | HeLa229 | **11**,**12** | 12,**13.3** | 8,**12** | **9**,10 | **16**,**18** | **7**,7 | **X**,**X** | 8,**12** | **9**,**10** | - |
| 0.65(26/40) | CRL-2182 | EPLC-32M1 | **11**,**12** | **13.3**,14.3 | 8,**12** | **9**,10 | **17**,**18** | **7**,7 | **X**,**X** | 8,**12**,13 | **9**,**10** | - |
| 0.65(26/40) | JCRB1082 | KYSE170 | **11**,**12** | 9,9 | **11**,**12** | **9**,10 | **16**,**17** | **7**,**9** | **X**,**X** | 11,11 | **10**,**12**,13 | - |
| 0.65(26/40) | JCRB9010 | HeLa S3 | **11**,**12** | **13.3**,13.3 | 8,8 | **9**,10 | **16**,**17**,**18** | **7**,7 | **X**,**X** | 8,**12** | **9**,**10** | - |
| 0.65(24/37) | RCB0191 | HeLa.S3 | **11**,**12** |  | 8,**12** | **9**,10 | **16**,**18** | **7**,7 | **X**,**X** | 8,**12** | **9**,**10** | - |
| 0.65(24/37) | RCB0205 | BU25 TK- | **11**,**12** |  | 8,**12** | **9**,10 | **16**,**18** | **7**,7 | **X**,**X** | 8,**12** | **9**,**10** | - |
| 0.65(24/37) | RCB1525 | HeLa.S3 | **11**,**12** |  | 8,**12** | **9**,10 | **16**,**18** | **7**,7 | **X**,**X** | 8,**12** | **9**,**10** | - |
| 0.65(24/37) | RCB1891 | HeLa TG | **11**,**12** |  | 8,8 | **9**,10 | **16**,**18** | **7**,7 | **X**,**X** | **12**,**12** | **9**,**10** | - |
| 0.63(26/41) | RCB0608 | SF8543 | **10**,**11**,**12** | 8,11 | **11**,**12** | **9**,**11** | **16**,**17**,19 | **7**,**9** | **X**,**X** | 8,8 | 11,13 | - |
| 0.63(24/38) | RCB2105 | D98-AH2 | **11**,**12** |  | **12**,12 | **9**,10 | **16**,**18** | **7**,7 | **X**,**X** | 8,**12** | **9**,**10**,11 | - |
| 0.62(24/39) | CRL-1647 | ST486 | **11**,**12** | 8,12 | 7,**12** | **9**,12 | **17**,**18** | **7**,**9** | **X**,**X** | 8,11 | **10**,**12** | - |
| 0.62(24/39) | CRL-5972 | NCI-SNU-C1 | **10**,10 | 9,11 | 10,**11** | **9**,**11** | **16**,**18** | **7**,**9** | **X**,**X** | 11,**12** | **12**,12 | - |
| 0.62(24/39) | CRL-7440 | Hs 701.T | **10**,**12** | 11,12 | **11**,**12** | **11**,11 | **17**,**18** | 6,**9** | **X**,**X** | 8,11 | **10**,**12** | - |
| 0.62(24/39) | IFO50011 | HeLa S3 | **11**,11 | 12,**13.3** | 8,**12** | **9**,10 | **17**,**18** | **7**,7 | **X**,**X** | 8,**12** | **9**,**10** | - |
| 0.62(24/39) | JCRB0118 | SKM-1 | **10**,**12** | 11,13 | 10,**11** | **9**,**11** | **18**,18 | **7**,**9** | **X**,**X** | 8,11 | **10**,**12** | - |
| 0.62(24/39) | JCRB0713 | HeLa S3(sc) | **11**,11 | 12,**13.3** | 8,**12** | **9**,10 | **17**,**18** | **7**,7 | **X**,**X** | 8,**12** | **9**,**10** | - |
| 0.62(24/39) | JCRB1074 | FU97 | **10**,**12** | 10,12 | **11**,11 | **9**,**11** | **17**,17 | **9**,9 | **X**,**X** | **12**,**12** | **12**,12 | - |
| 0.62(24/39) | JCRB1179 | KMS-11 | **10**,**12** | 12,12 | **11**,**12** | **9**,13 | **17**,**18** | 6,**9** | **X**,**X** | **12**,**12** | 13,13 | - |
| 0.62(24/39) | JCRB1309 | RPMI4788 | **11**,**12** | **13.3**,13.3 | 8,**12** | 10,10 | **16**,**18** | **7**,7 | **X**,**X** | 8,**12** | **9**,**10** | - |
| 0.62(24/39) | RCB0007 | HeLa | **11**,**12** | 12,12 | 8,**12** | **9**,10 | **16**,**18** | **7**,7 | **X**,**X** | 8,**12** | **9**,**10** | - |
| 0.62(24/39) | RCB0083 | HLC-1 | 9,**10** | 8,8 | **11**,13 | **9**,12 | **16**,**17** | **7**,**9** | **X**,**X** | **12**,**12** | 7,**12** | - |
| 0.62(24/39) | RCB0402 | "HeLa�P3" | **11**,**12** | 12,12 | 8,**12** | **9**,10 | **16**,**18** | **7**,7 | **X**,**X** | 8,**12** | **9**,**10** | - |
| 0.62(24/39) | RCB0443 | TC-YIK | **10**,**11** | 8,9 | 8,**11** | **9**,**11** | **17**,**18** | **7**,**9** | **X**,**X** | 11,11 | 11,**12** | - |
| 0.62(24/39) | RCB0785 | FCP-S3H | **10**,**11** | 8,12 | **11**,**12** | **9**,10 | **17**,**18** | **7**,7 | **X**,**X** | 8,9 | **9**,**10** | - |
| 0.62(24/39) | RCB0788 | FCP-S6H | **11**,**12** | 8,10 | **11**,**12** | 10,10 | **18**,19 | **7**,**9** | **X**,**X** | 8,**12** | **10**,**12** | - |
| 0.62(24/39) | RCB0967 | HTST | **10**,**12** | **7**,12 | **12**,12 | **9**,10 | **16**,**18** | **9**,9 | **X**,**X** | 11,11 | **10**,**12** | - |
| 0.62(24/39) | RCB1889 | HEp-2 | **11**,**12** | 12,12 | 8,**12** | **9**,10 | **16**,**18** | **7**,7 | **X**,**X** | 8,**12** | **9**,**10** | - |
| 0.62(24/39) | RCB2251 | HE34 | **11**,13 | 11,11 | **11**,11 | **9**,**11** | **17**,**18** | **7**,**9** | **X**,**X** | 8,8 | **10**,**12** | - |
| 0.62(24/39) | RCB2355 | HeLa CD4+Clone1022 | **11**,**12** | 12,12 | 8,**12** | **9**,9 | **16**,**18** | **7**,7 | **X**,**X** | 8,**12** | **9**,**10** | - |
| 0.62(24/39) | RCB2356 | HeLa-CD4-LTR-??-gal | **11**,**12** | 12,12 | 8,**12** | **9**,10 | **16**,**18** | **7**,7 | **X**,**X** | 8,**12** | **9**,**10** | - |
| 0.62(24/39) | RCB2358 | HLtat | **11**,**12** | 12,12 | 8,**12** | **9**,10 | **16**,**18** | **7**,7 | **X**,**X** | 8,**12** | **9**,**10** | - |
| 0.60(26/43) | JCRB0105.1 | KY821A3 | 9,**10**,**11** | 8,9,11 | **11**,**12** | 10,**11**,12 | 14,**17**,**18** | **7**,**9** | **X**,**X** | 8,11 | **10**,**12** | - |

  
  
  


---


 **after changing the EV
1.00
0.90
0.80
0.70
0.60
0.50
0.40
0.30
0.20
0.10
0.00
0.00


| EV | Cell No. Scored | Graph |
| --- | --- | --- |
| **0.95**~1.00 | 0 |  |
| **0.90**~0.95 | 0 |  |
| **0.85**~0.90 | 0 |  |
| **0.80**~0.85 | 0 |  |
| **0.75**~0.80 | 0 |  |
| **0.70**~0.75 | 2 | || |
| **0.65**~0.70 | 49 | ||||||||||||||||||||||||||||||||||||||||||||||||| |
| **0.60**~0.65 | 24 | |||||||||||||||||||||||| |
| 0.55~0.60 | 66 | |||||||||||||||||||||||||||||||||||||||||||||||||||||||||||||||||| |
| 0.50~0.55 | 166 | |||||||||||||||||||||||||||||||||||||||||||||||||||||||||||||||||||||||||||||||||||||||||||||||||||||||||||||||||||||||||||||||||||||||||||||||||||||||||||||||||||||| |
| 0.45~0.50 | 410 | |||||||||||||||||||||||||||||||||||||||||||||||||||||||||||||||||||||||||||||||||||||||||||||||||||||||||||||||||||||||||||||||||||||||||||||||||||||||||||||||||||||||||||||||||||||||||||||||||||||||||||||||||||||||||||||||||||||||||||||||||||||||||||||||||||||||||||||||||||||||||||||||||||||||||||||||||||||||||||||||||||||||||||||||||||||||||||||||||||||||||||||||||||||||||||||||||||||||||||||||||||||||||| |
| 0.40~0.45 | 680 | |||||||||||||||||||||||||||||||||||||||||||||||||||||||||||||||||||||||||||||||||||||||||||||||||||||||||||||||||||||||||||||||||||||||||||||||||||||||||||||||||||||||||||||||||||||||||||||||||||||||||||||||||||||||||||||||||||||||||||||||||||||||||||||||||||||||||||||||||||||||||||||||||||||||||||||||||||||||||||||||||||||||||||||||||||||||||||||||||||||||||||||||||||||||||||||||||||||||||||||||||||||||||||||||||||||||||||||||||||||||||||||||||||||||||||||||||||||||||||||||||||||||||||||||||||||||||||||||||||||||||||||||||||||||||||||||||||||||||||||||||||||||||||||||||||||||||||||||||||||||||||||||||||||||||||||||||||||||||||||||||||||||||||||||||||||||||||||||||||||||| |
| 0.35~0.40 | 798 | |||||||||||||||||||||||||||||||||||||||||||||||||||||||||||||||||||||||||||||||||||||||||||||||||||||||||||||||||||||||||||||||||||||||||||||||||||||||||||||||||||||||||||||||||||||||||||||||||||||||||||||||||||||||||||||||||||||||||||||||||||||||||||||||||||||||||||||||||||||||||||||||||||||||||||||||||||||||||||||||||||||||||||||||||||||||||||||||||||||||||||||||||||||||||||||||||||||||||||||||||||||||||||||||||||||||||||||||||||||||||||||||||||||||||||||||||||||||||||||||||||||||||||||||||||||||||||||||||||||||||||||||||||||||||||||||||||||||||||||||||||||||||||||||||||||||||||||||||||||||||||||||||||||||||||||||||||||||||||||||||||||||||||||||||||||||||||||||||||||||||||||||||||||||||||||||||||||||||||||||||||||||||||||||||||||||||||||||||||||||||||||||||||||||||||||||||||||||||||||| |
| 0.30~0.35 | 583 | ||||||||||||||||||||||||||||||||||||||||||||||||||||||||||||||||||||||||||||||||||||||||||||||||||||||||||||||||||||||||||||||||||||||||||||||||||||||||||||||||||||||||||||||||||||||||||||||||||||||||||||||||||||||||||||||||||||||||||||||||||||||||||||||||||||||||||||||||||||||||||||||||||||||||||||||||||||||||||||||||||||||||||||||||||||||||||||||||||||||||||||||||||||||||||||||||||||||||||||||||||||||||||||||||||||||||||||||||||||||||||||||||||||||||||||||||||||||||||||||||||||||||||||||||||||||||||||||||||||||||||||||||||||||||||||||||||||||||||||||||||||||||||||||||||||||| |
| 0.25~0.30 | 306 | |||||||||||||||||||||||||||||||||||||||||||||||||||||||||||||||||||||||||||||||||||||||||||||||||||||||||||||||||||||||||||||||||||||||||||||||||||||||||||||||||||||||||||||||||||||||||||||||||||||||||||||||||||||||||||||||||||||||||||||||||||||||||||||||||||||||||||||||||||||||||||||||||||||||||||||||||| |
| 0.20~0.25 | 142 | |||||||||||||||||||||||||||||||||||||||||||||||||||||||||||||||||||||||||||||||||||||||||||||||||||||||||||||||||||||||||||||||||||||||||||||| |
| 0.15~0.20 | 35 | ||||||||||||||||||||||||||||||||||| |
| 0.10~0.15 | 3 | ||| |
| 0.05~0.10 | 0 |  |
| 0.00~0.05 | 10 | |||||||||| |
| Total No. of cells = 3274 (DSMZ Cell Bank) | | |

  

---

  
If the results obtained by the search engine are used in any publication, please cite the respective paper: Dirks WG, MacLeod RA, Nakamura Y, Kohara A, Reid Y, Milch H, Drexler HG, Mizusawa H.: Cell line cross-contamination initiative: an interactive reference database of STR profiles covering common cancer cell lines. Int J Cancer. 2010 Jan 1;126(1):303-4. (link: https://onlinelibrary.wiley.com/doi/full/10.1002/ijc.24999)**
