# Supplementary figures and images for: DLAT inhibits ferroptosis to promote malignant progression of gastric cancer through Nrf2/HO-1/GPX4 signaling pathway
Source: Biol Direct. 2026 Apr 2;21:64. doi: 10.1186/s13062-026-00767-7 (PMC13162497; doi:10.1186/s13062-026-00767-7)

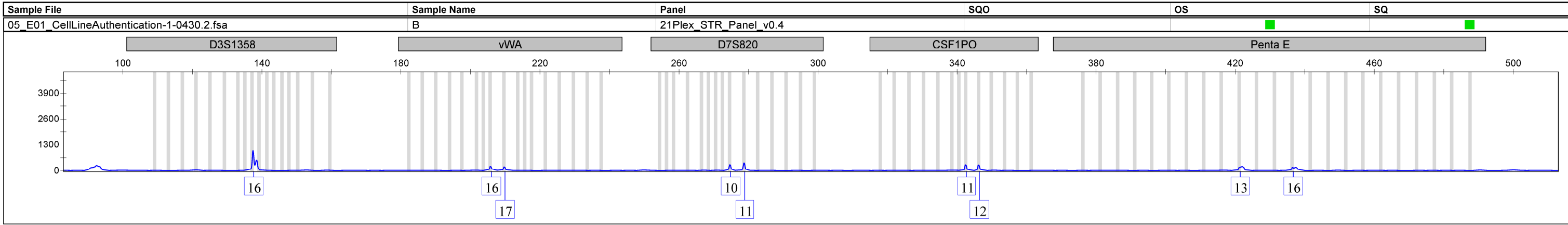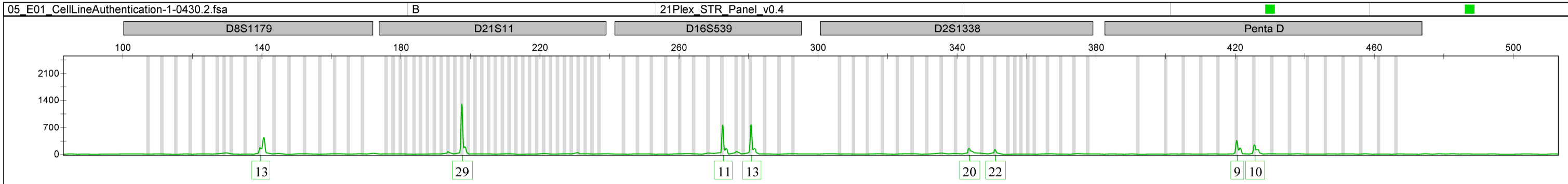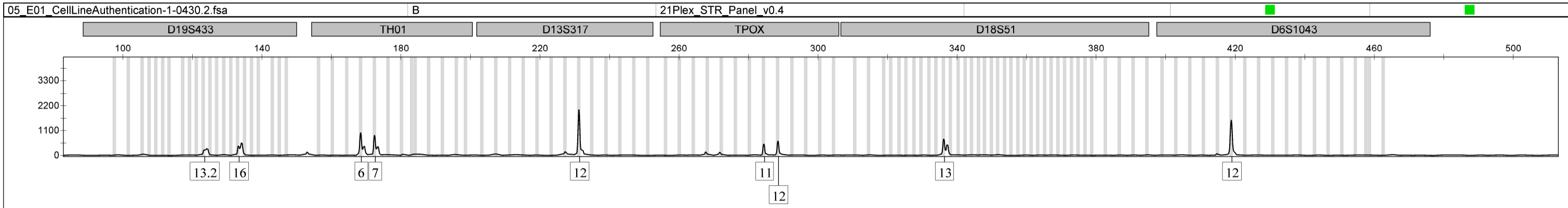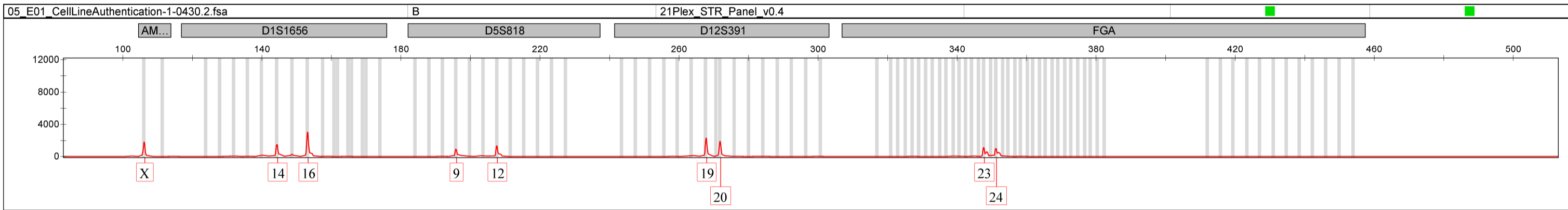

Supplement: Supplementary file 1 — Supplementary Material 1 [file 13062_2026_767_MOESM1_ESM.zip › Cell line authentication/AGS Cell line authentication/AGS.pdf]

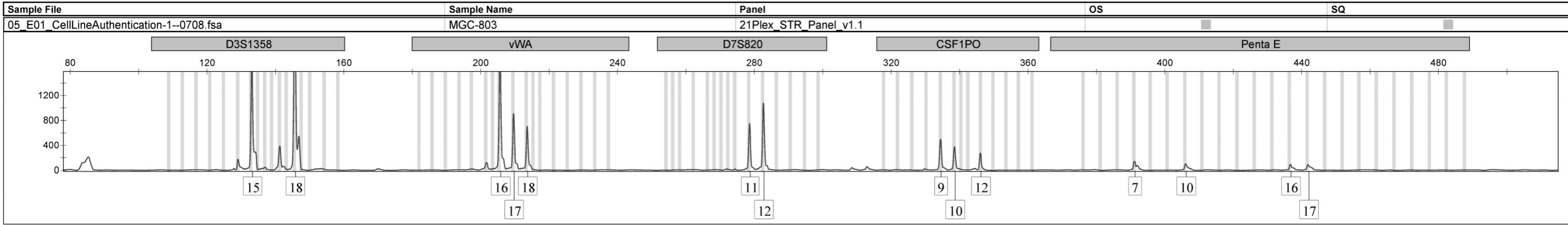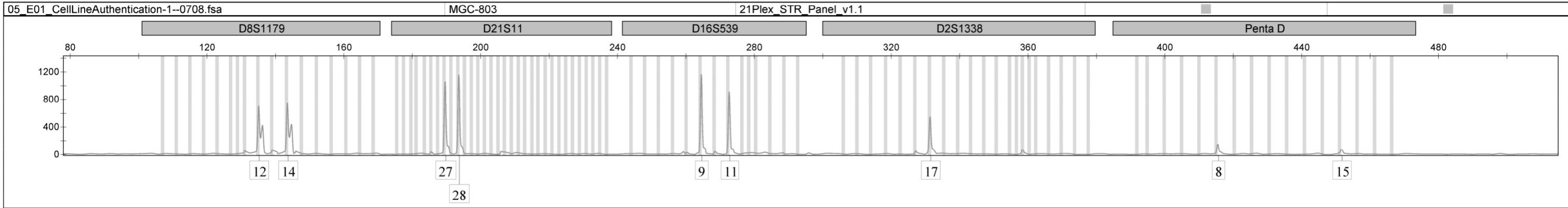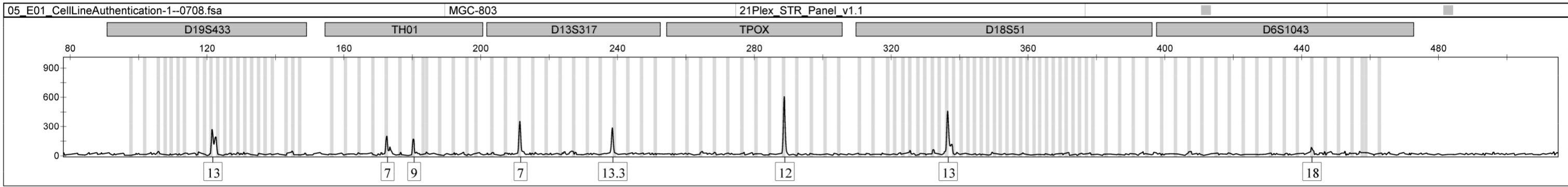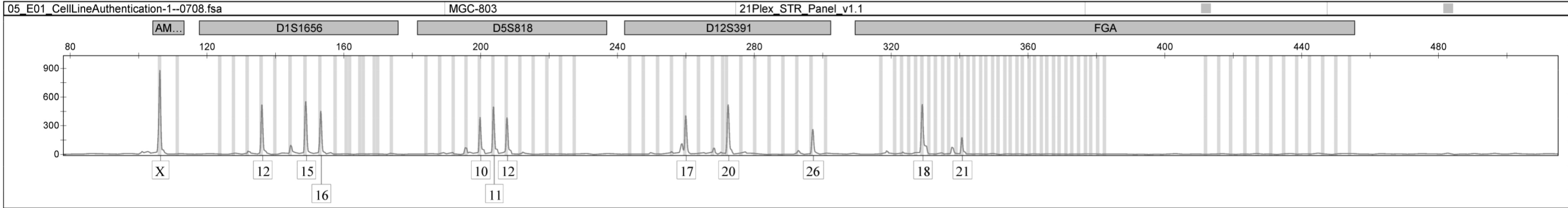

Supplement: Supplementary file 1 — Supplementary Material 1 [file 13062_2026_767_MOESM1_ESM.zip › Cell line authentication/MGC-803 Cell line authentication/MGC-803.pdf]

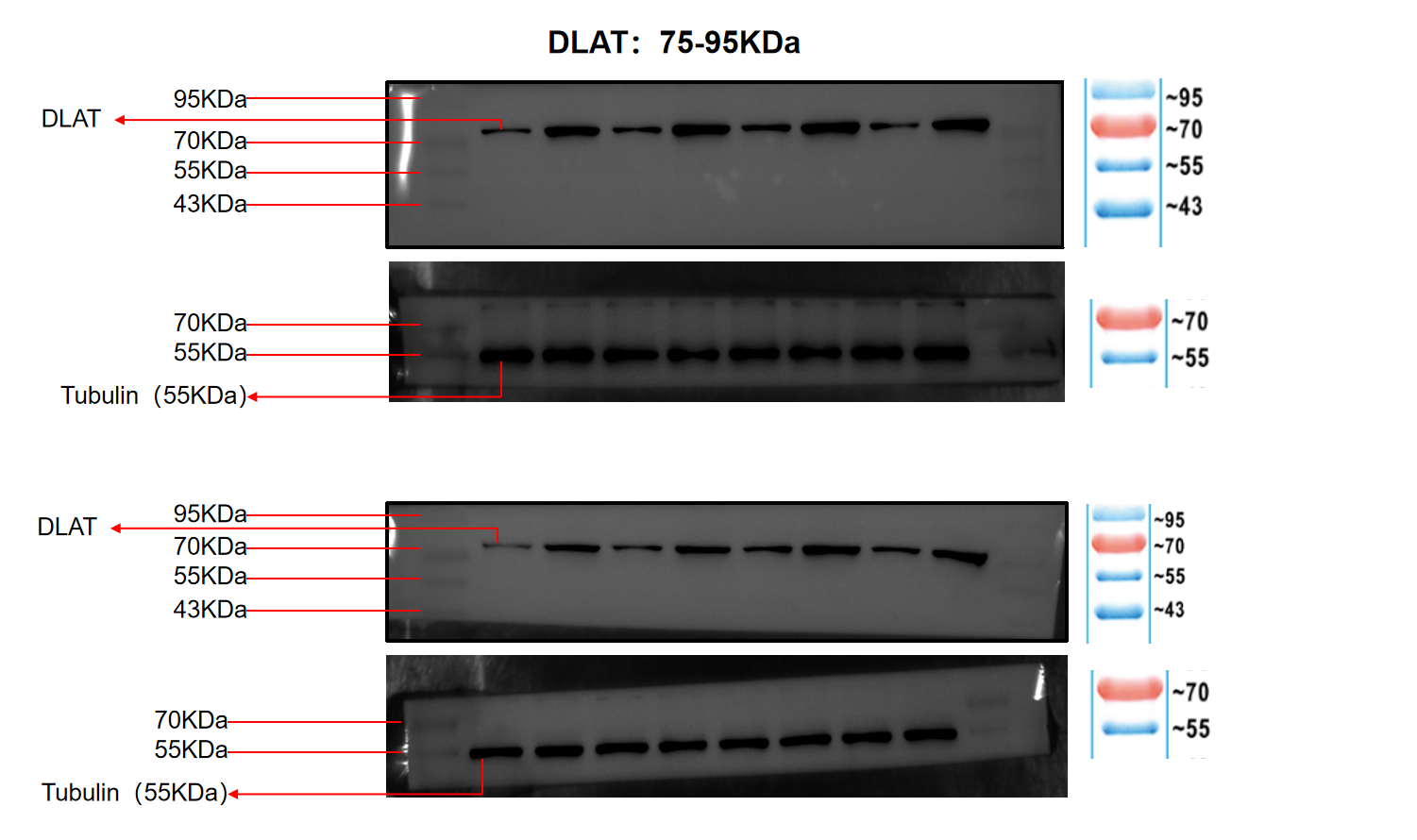

Supplement: Supplementary file 2 — Supplementary Material 2 [file 13062_2026_767_MOESM2_ESM.zip › supplementary file2/Figure 1B.tif]

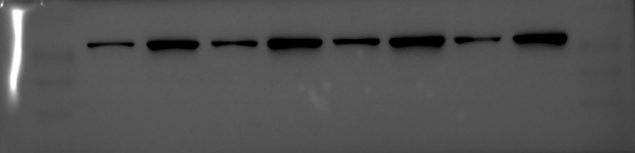

Supplement: Supplementary file 2 — Supplementary Material 2 [file 13062_2026_767_MOESM2_ESM.zip › supplementary file2/Figure 1B_1 (3).jpg]

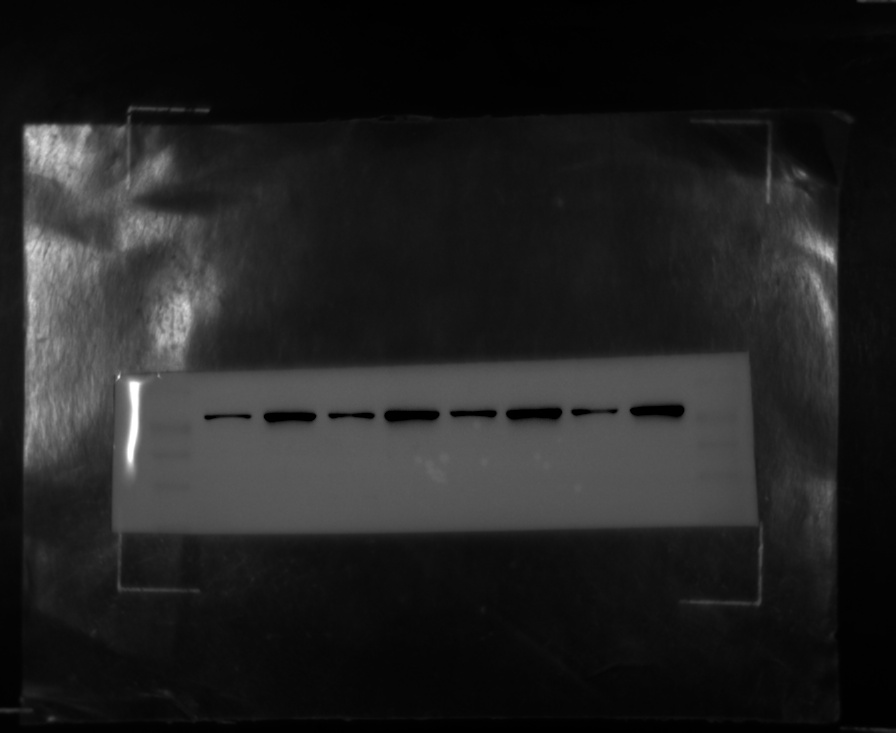

Supplement: Supplementary file 2 — Supplementary Material 2 [file 13062_2026_767_MOESM2_ESM.zip › supplementary file2/Figure 1B_1(2).jpg]

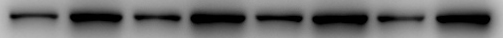

Supplement: Supplementary file 2 — Supplementary Material 2 [file 13062_2026_767_MOESM2_ESM.zip › supplementary file2/Figure 1B_1.jpg]

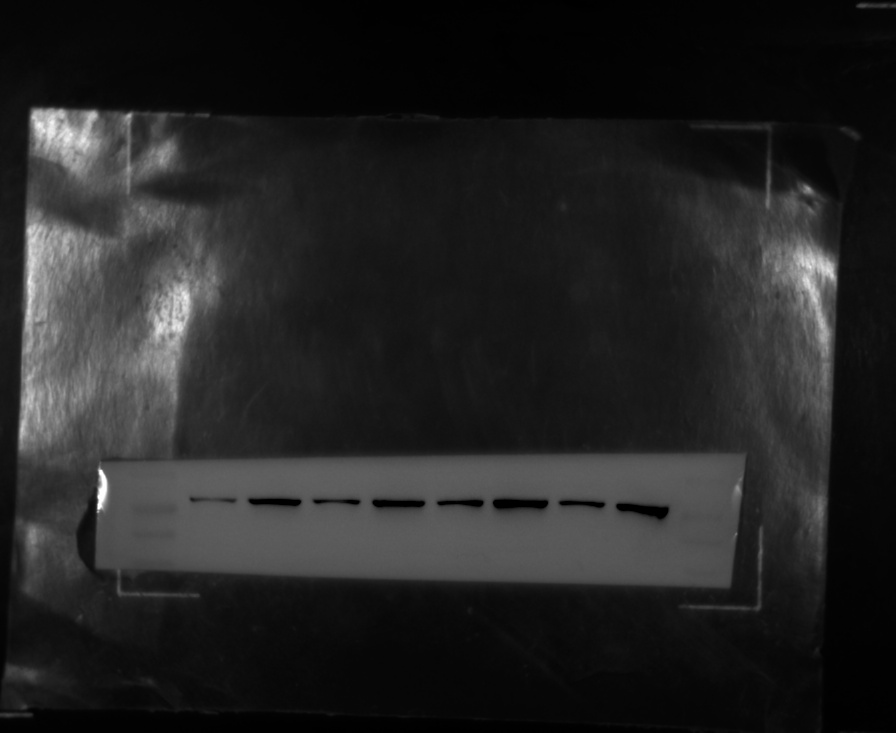

Supplement: Supplementary file 2 — Supplementary Material 2 [file 13062_2026_767_MOESM2_ESM.zip › supplementary file2/Figure 1B_2 (2).jpg]

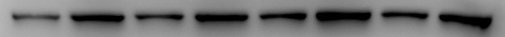

Supplement: Supplementary file 2 — Supplementary Material 2 [file 13062_2026_767_MOESM2_ESM.zip › supplementary file2/Figure 1B_2.0.jpg]

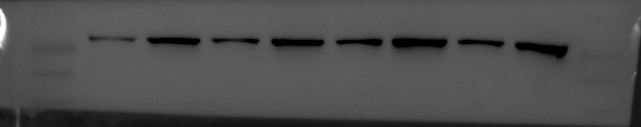

Supplement: Supplementary file 2 — Supplementary Material 2 [file 13062_2026_767_MOESM2_ESM.zip › supplementary file2/Figure 1B_2.jpg]

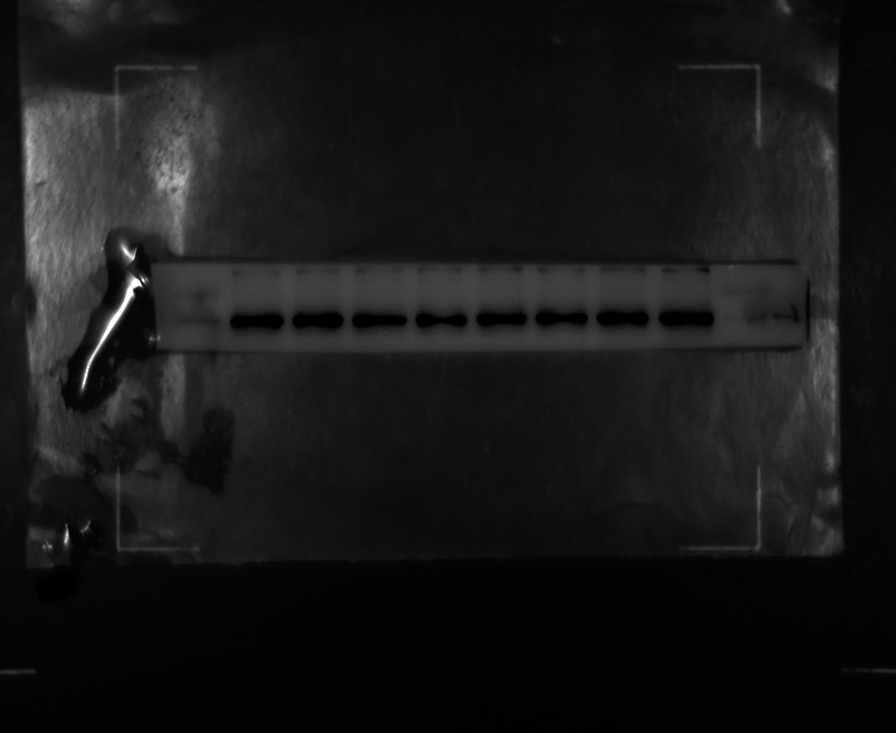

Supplement: Supplementary file 2 — Supplementary Material 2 [file 13062_2026_767_MOESM2_ESM.zip › supplementary file2/Figure 1B_tubulin(2).jpg]

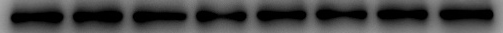

Supplement: Supplementary file 2 — Supplementary Material 2 [file 13062_2026_767_MOESM2_ESM.zip › supplementary file2/Figure 1B_tubulin.jpg]

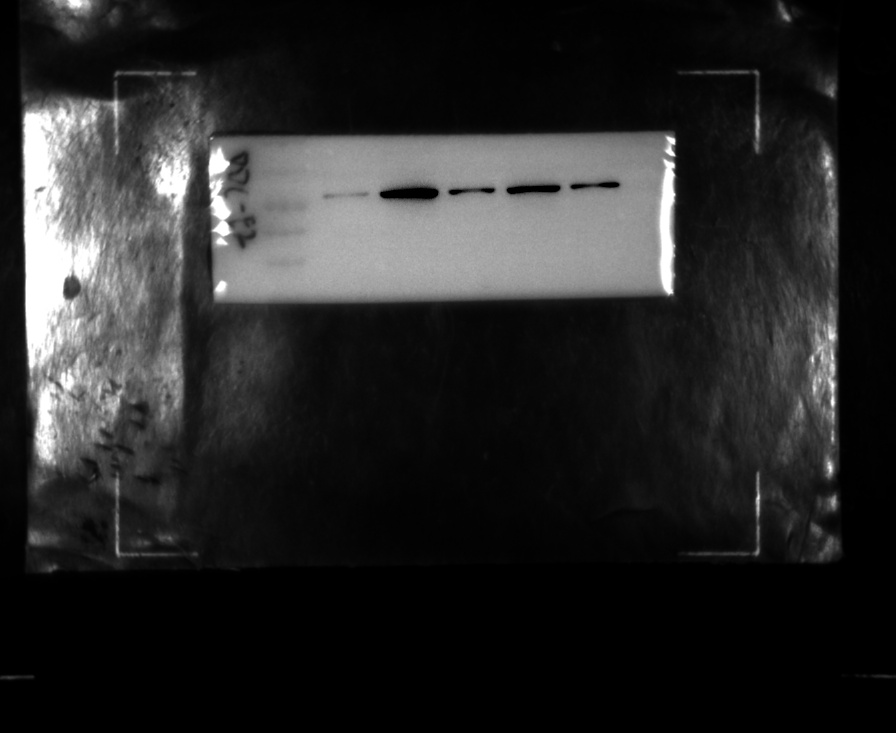

Supplement: Supplementary file 2 — Supplementary Material 2 [file 13062_2026_767_MOESM2_ESM.zip › supplementary file2/Figure 1D(2).jpg]

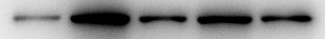

Supplement: Supplementary file 2 — Supplementary Material 2 [file 13062_2026_767_MOESM2_ESM.zip › supplementary file2/Figure 1D.jpg]

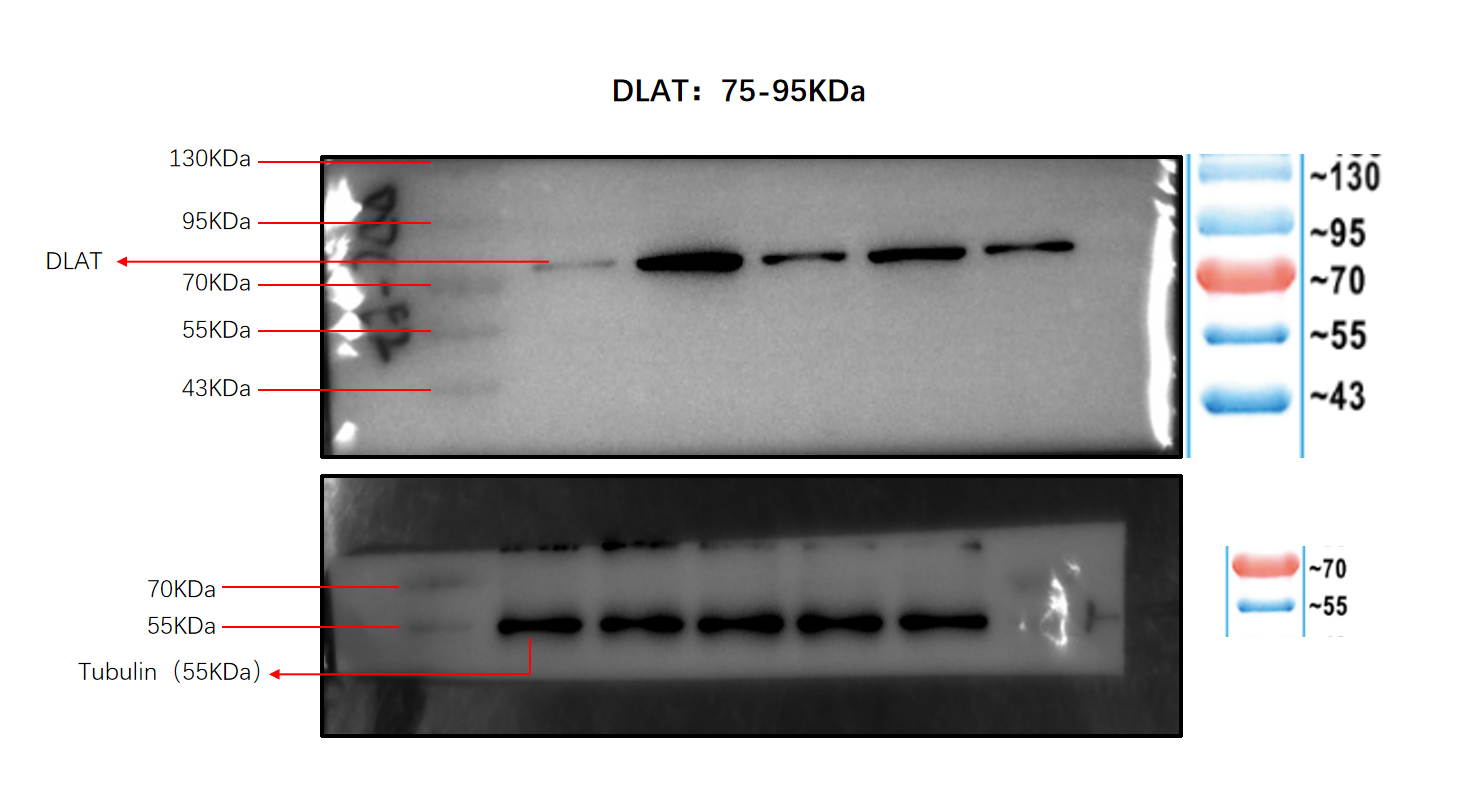

Supplement: Supplementary file 2 — Supplementary Material 2 [file 13062_2026_767_MOESM2_ESM.zip › supplementary file2/Figure 1D.tif]

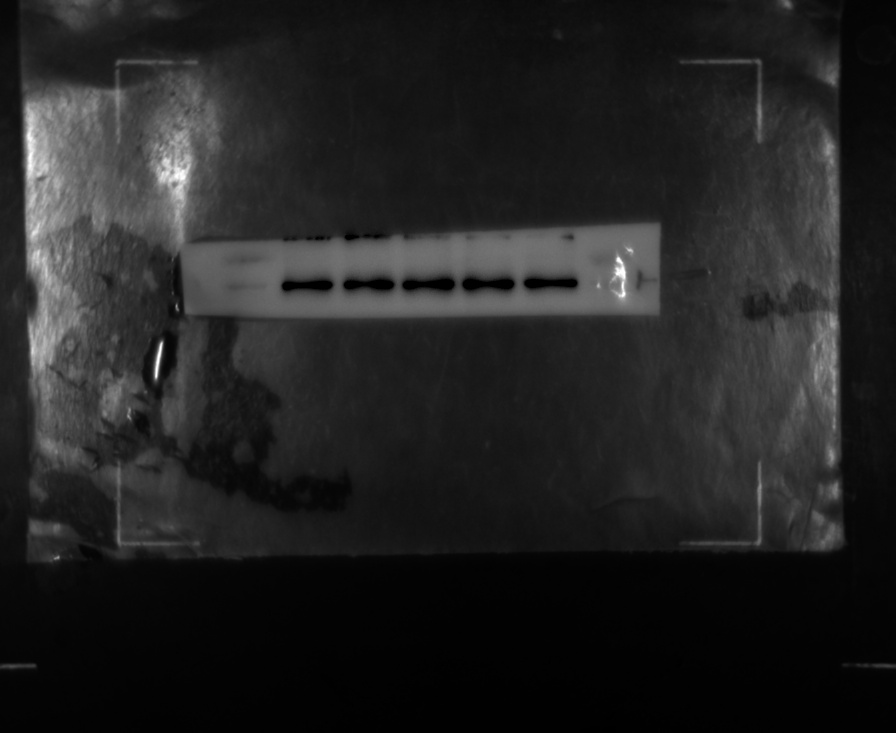

Supplement: Supplementary file 2 — Supplementary Material 2 [file 13062_2026_767_MOESM2_ESM.zip › supplementary file2/Figure 1D_tubulin(2).jpg]

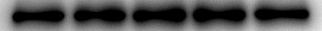

Supplement: Supplementary file 2 — Supplementary Material 2 [file 13062_2026_767_MOESM2_ESM.zip › supplementary file2/Figure 1D_tubulin.jpg]

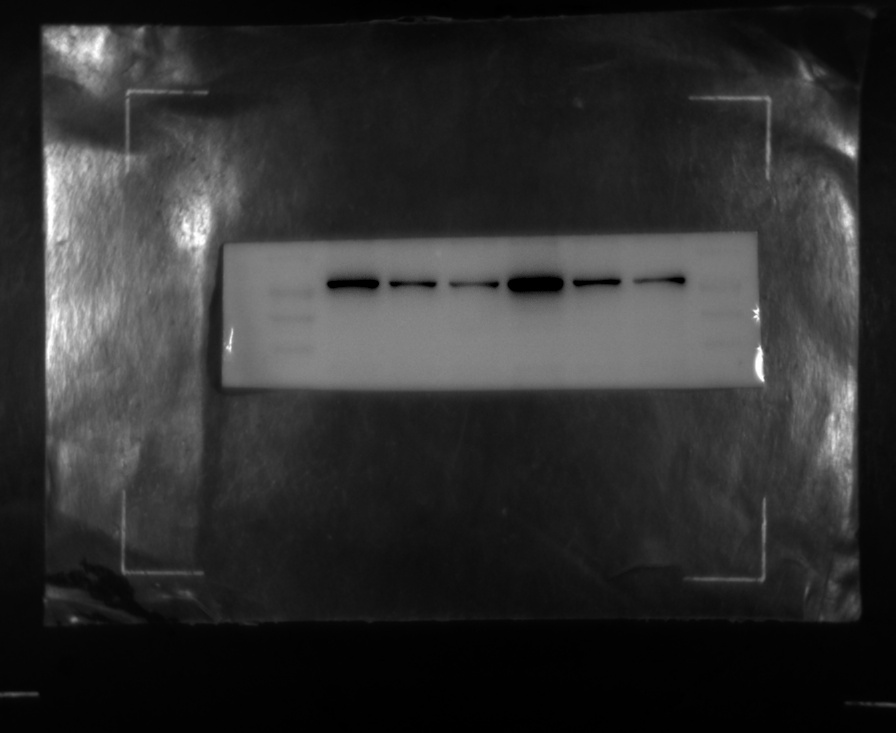

Supplement: Supplementary file 2 — Supplementary Material 2 [file 13062_2026_767_MOESM2_ESM.zip › supplementary file2/Figure 2B(2).jpg]

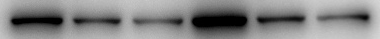

Supplement: Supplementary file 2 — Supplementary Material 2 [file 13062_2026_767_MOESM2_ESM.zip › supplementary file2/Figure 2B.jpg]

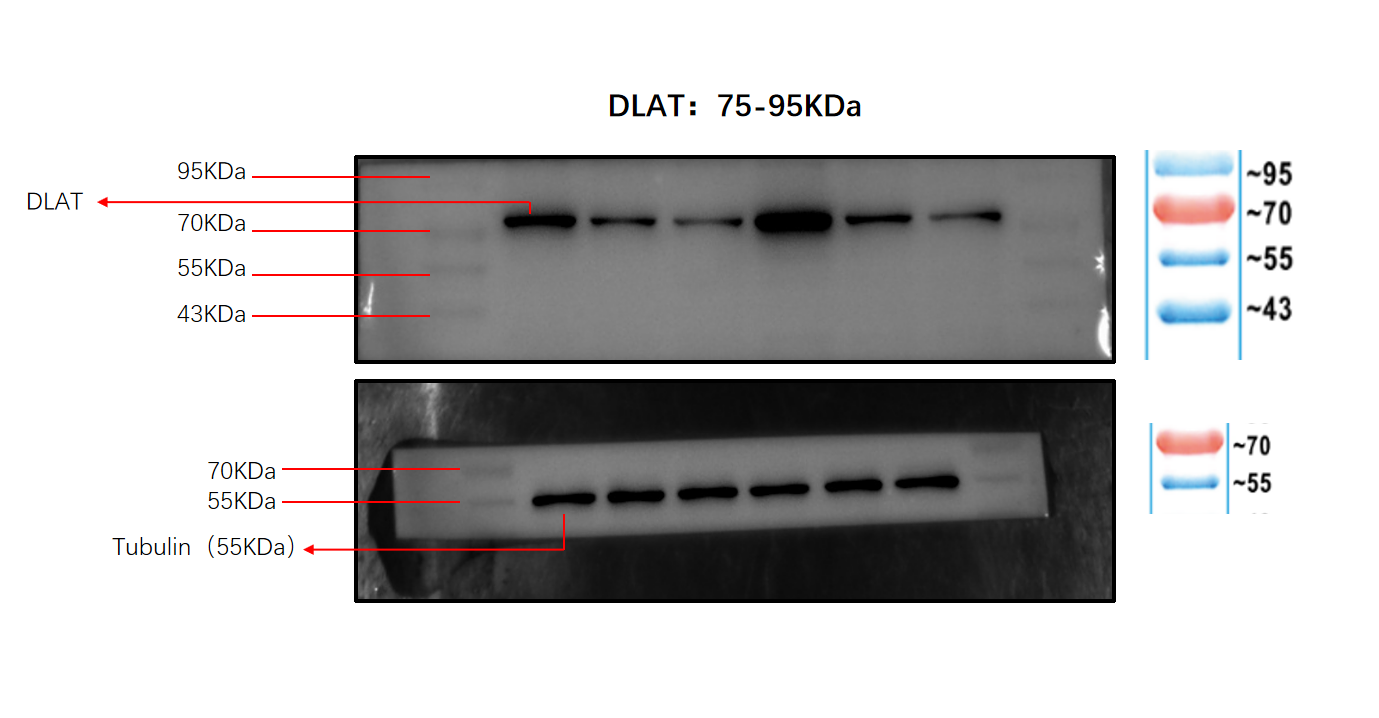

Supplement: Supplementary file 2 — Supplementary Material 2 [file 13062_2026_767_MOESM2_ESM.zip › supplementary file2/Figure 2B.tif]

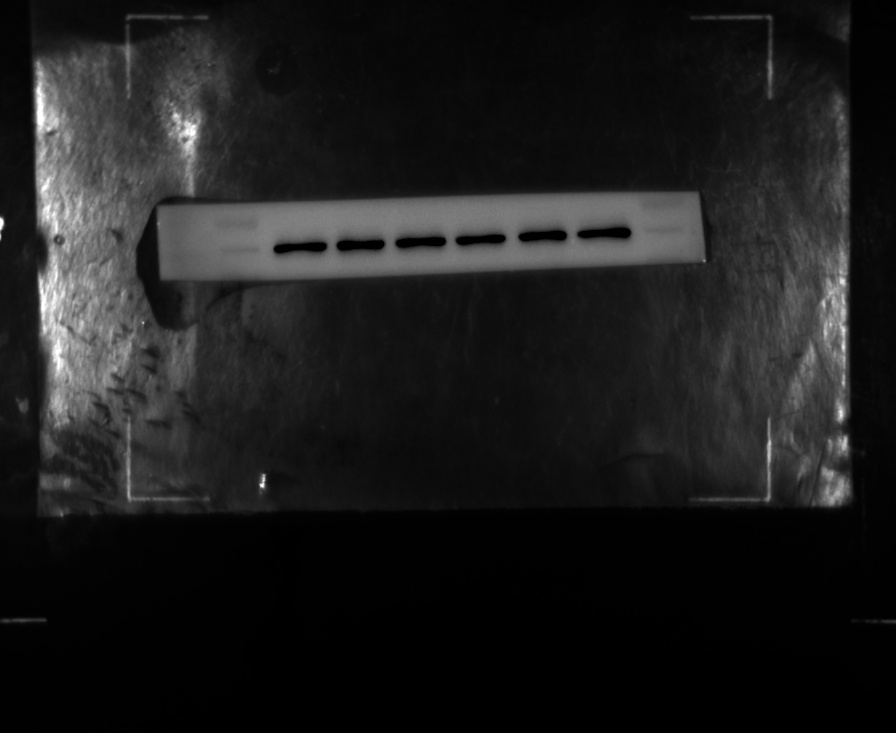

Supplement: Supplementary file 2 — Supplementary Material 2 [file 13062_2026_767_MOESM2_ESM.zip › supplementary file2/Figure 2B_tubulin(2).jpg]

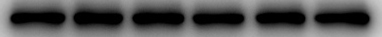

Supplement: Supplementary file 2 — Supplementary Material 2 [file 13062_2026_767_MOESM2_ESM.zip › supplementary file2/Figure 2B_tubulin.jpg]

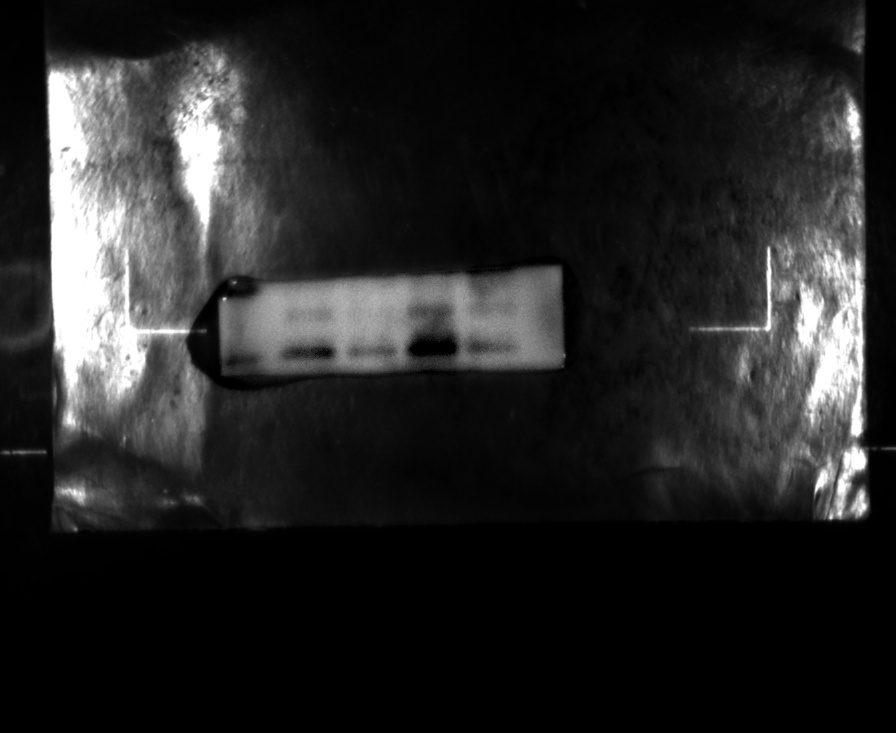

Supplement: Supplementary file 2 — Supplementary Material 2 [file 13062_2026_767_MOESM2_ESM.zip › supplementary file2/Figure 5C_FTH1(2).jpg]

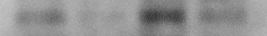

Supplement: Supplementary file 2 — Supplementary Material 2 [file 13062_2026_767_MOESM2_ESM.zip › supplementary file2/Figure 5C_FTH1.jpg]

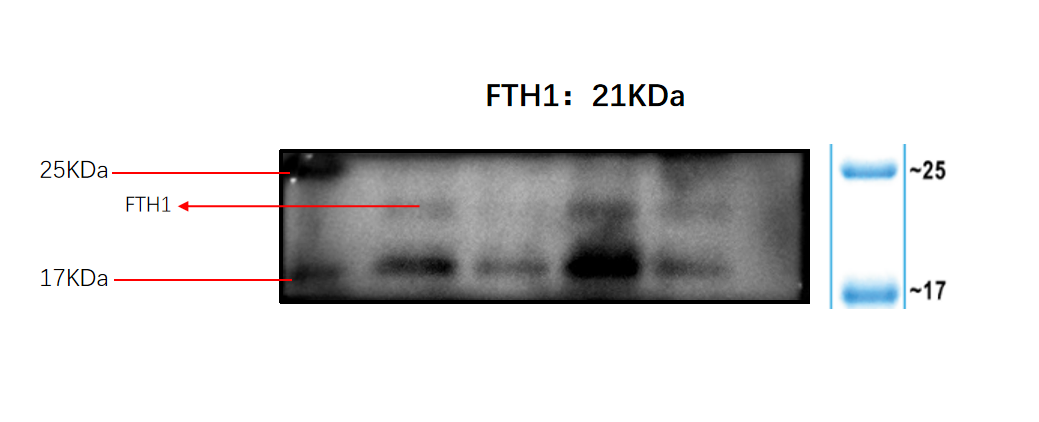

Supplement: Supplementary file 2 — Supplementary Material 2 [file 13062_2026_767_MOESM2_ESM.zip › supplementary file2/Figure 5C_FTH1.tif]

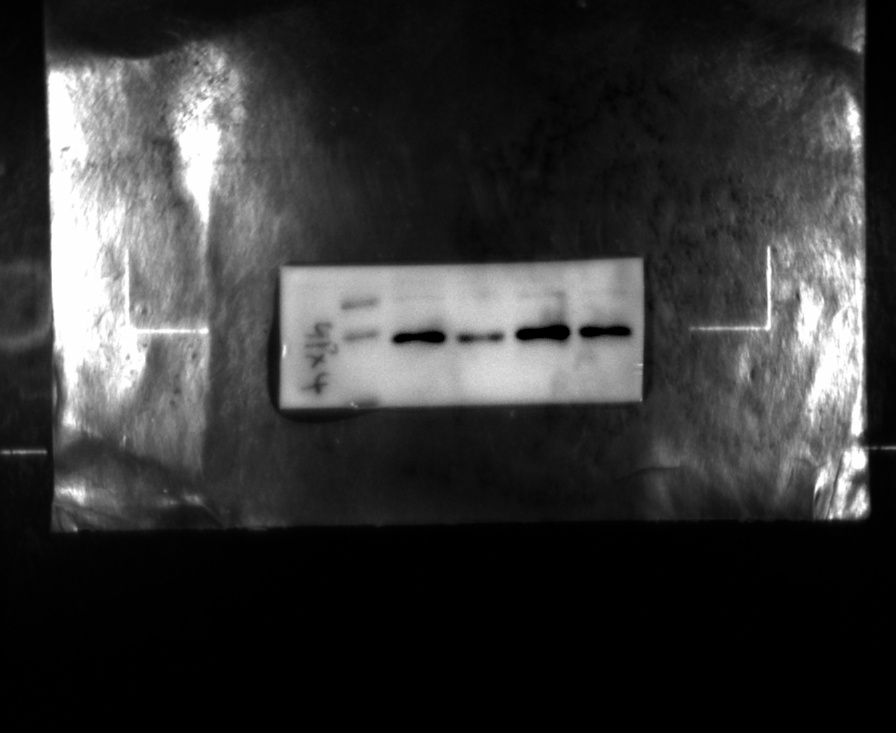

Supplement: Supplementary file 2 — Supplementary Material 2 [file 13062_2026_767_MOESM2_ESM.zip › supplementary file2/Figure 5C_GPX4(2).jpg]

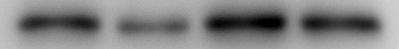

Supplement: Supplementary file 2 — Supplementary Material 2 [file 13062_2026_767_MOESM2_ESM.zip › supplementary file2/Figure 5C_GPX4.jpg]

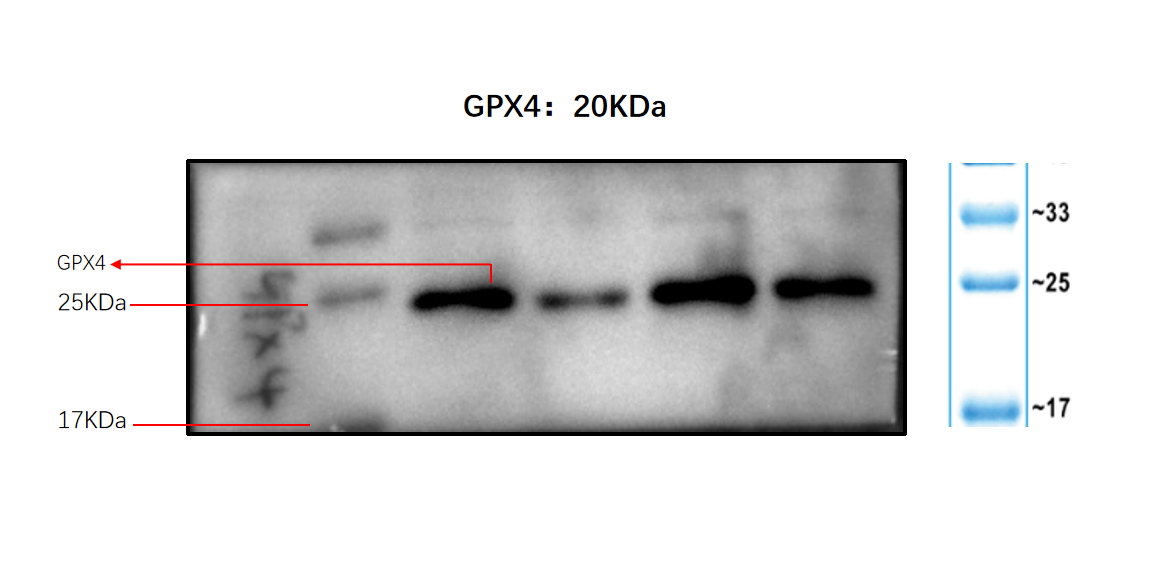

Supplement: Supplementary file 2 — Supplementary Material 2 [file 13062_2026_767_MOESM2_ESM.zip › supplementary file2/Figure 5C_GPX4.tif]

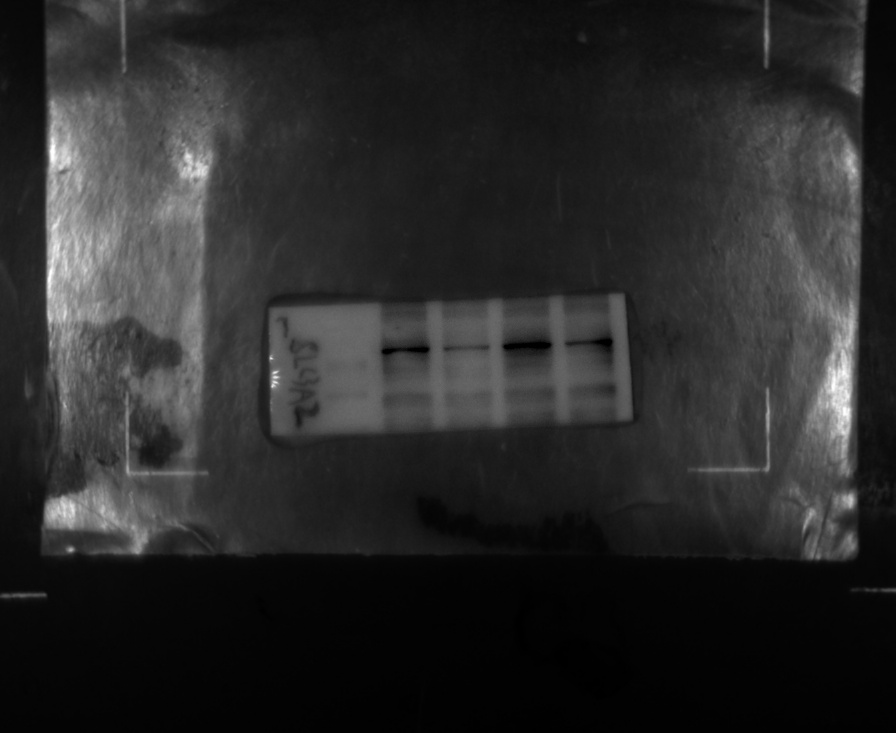

Supplement: Supplementary file 2 — Supplementary Material 2 [file 13062_2026_767_MOESM2_ESM.zip › supplementary file2/Figure 5C_SLC3A2(2).jpg]

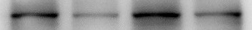

Supplement: Supplementary file 2 — Supplementary Material 2 [file 13062_2026_767_MOESM2_ESM.zip › supplementary file2/Figure 5C_SLC3A2.jpg]

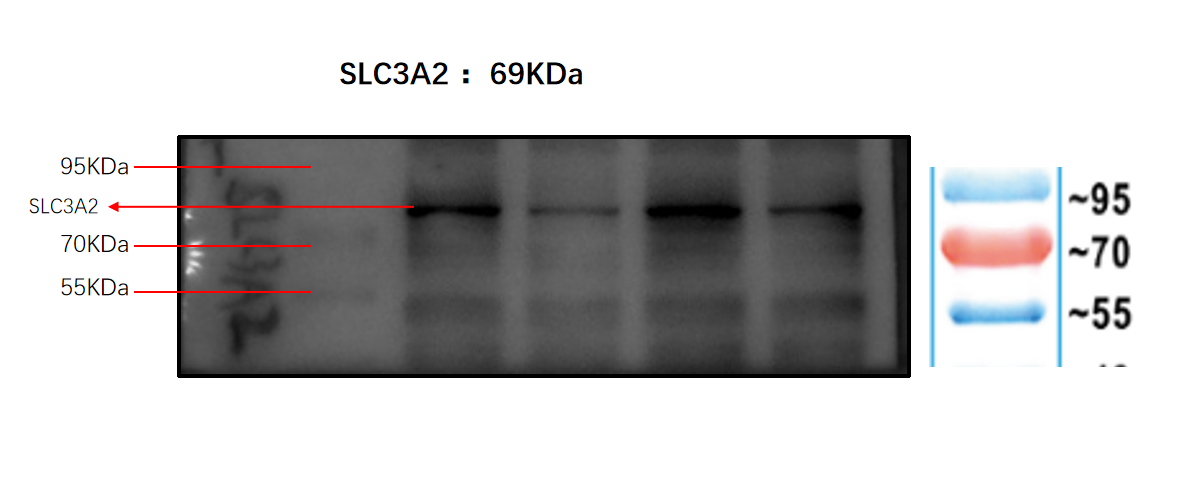

Supplement: Supplementary file 2 — Supplementary Material 2 [file 13062_2026_767_MOESM2_ESM.zip › supplementary file2/Figure 5C_SLC3A2.tif]

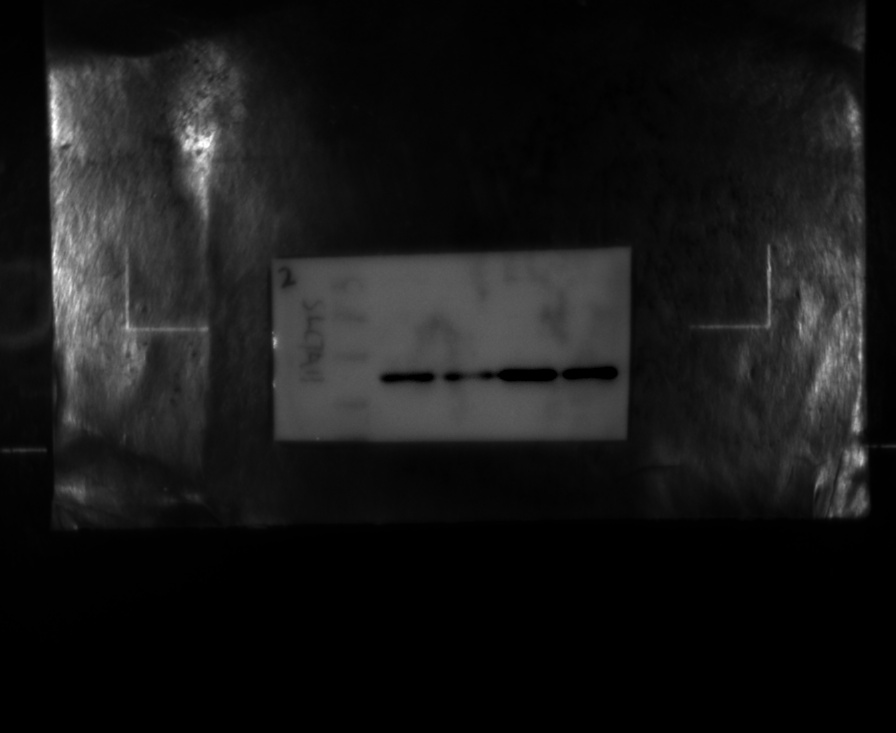

Supplement: Supplementary file 2 — Supplementary Material 2 [file 13062_2026_767_MOESM2_ESM.zip › supplementary file2/Figure 5C_SLC7A11(2).jpg]

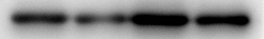

Supplement: Supplementary file 2 — Supplementary Material 2 [file 13062_2026_767_MOESM2_ESM.zip › supplementary file2/Figure 5C_SLC7A11.jpg]

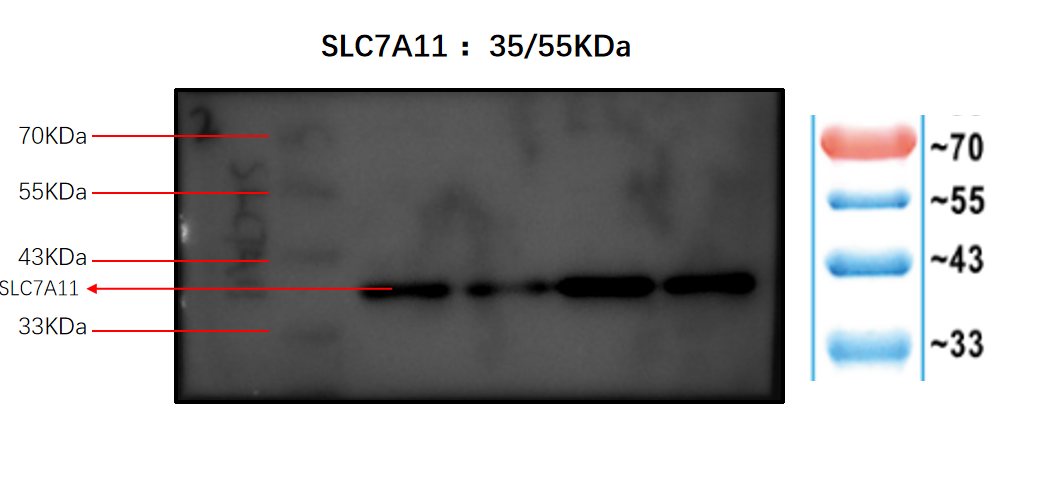

Supplement: Supplementary file 2 — Supplementary Material 2 [file 13062_2026_767_MOESM2_ESM.zip › supplementary file2/Figure 5C_SLC7A11.tif]

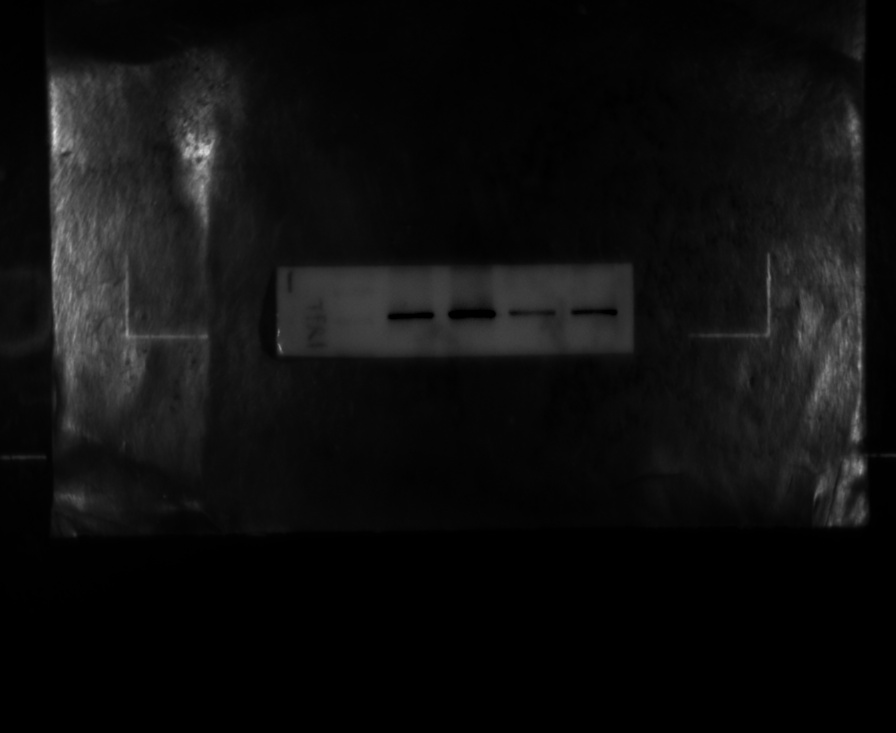

Supplement: Supplementary file 2 — Supplementary Material 2 [file 13062_2026_767_MOESM2_ESM.zip › supplementary file2/Figure 5C_TFR1(2).jpg]

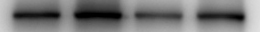

Supplement: Supplementary file 2 — Supplementary Material 2 [file 13062_2026_767_MOESM2_ESM.zip › supplementary file2/Figure 5C_TFR1.jpg]

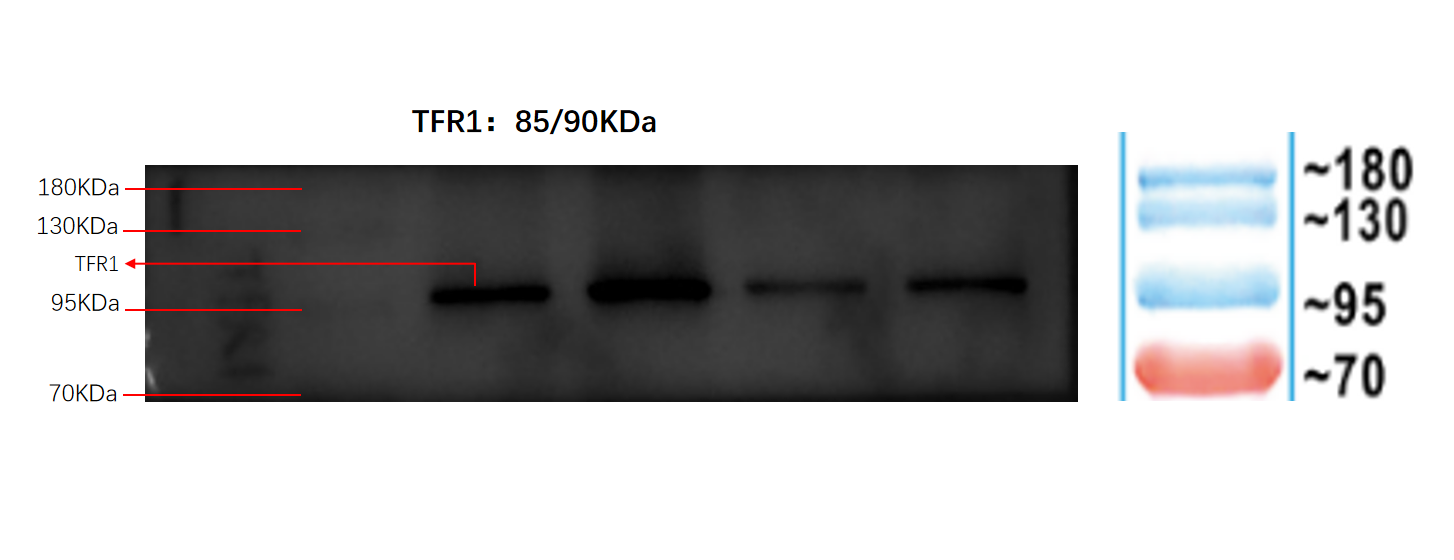

Supplement: Supplementary file 2 — Supplementary Material 2 [file 13062_2026_767_MOESM2_ESM.zip › supplementary file2/Figure 5C_TFR1.tif]

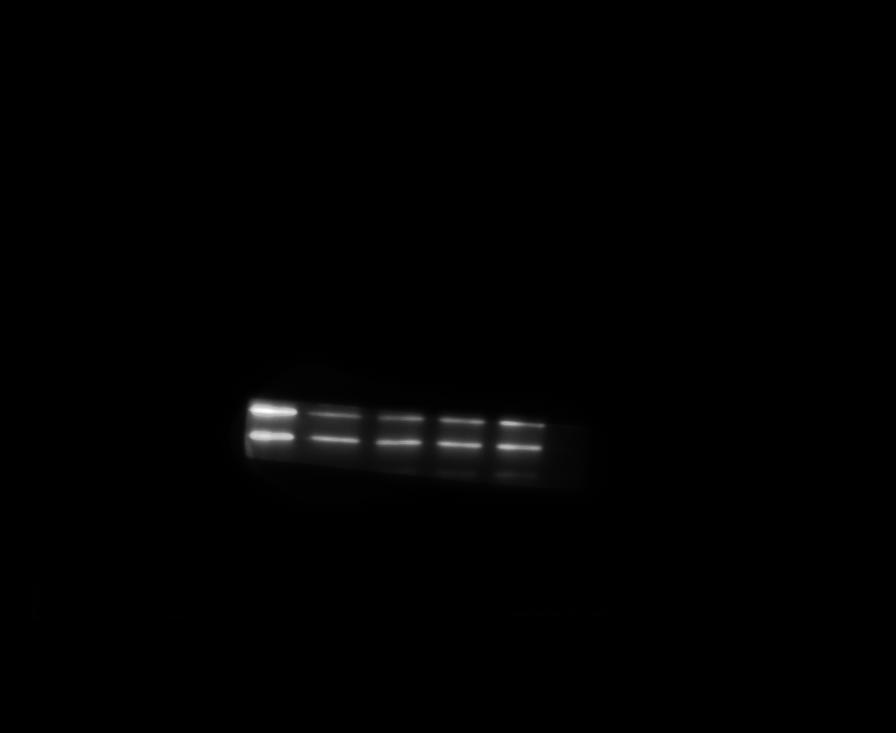

Supplement: Supplementary file 2 — Supplementary Material 2 [file 13062_2026_767_MOESM2_ESM.zip › supplementary file2/Figure 5C_tubulin(2).TIF]

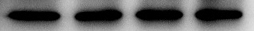

Supplement: Supplementary file 2 — Supplementary Material 2 [file 13062_2026_767_MOESM2_ESM.zip › supplementary file2/Figure 5C_tubulin.jpg]

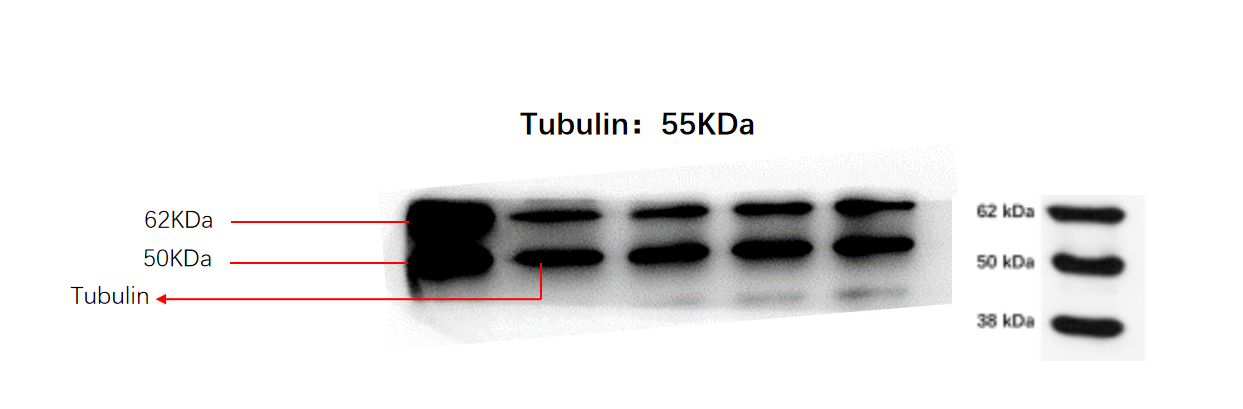

Supplement: Supplementary file 2 — Supplementary Material 2 [file 13062_2026_767_MOESM2_ESM.zip › supplementary file2/Figure 5C_tubulin.tif]

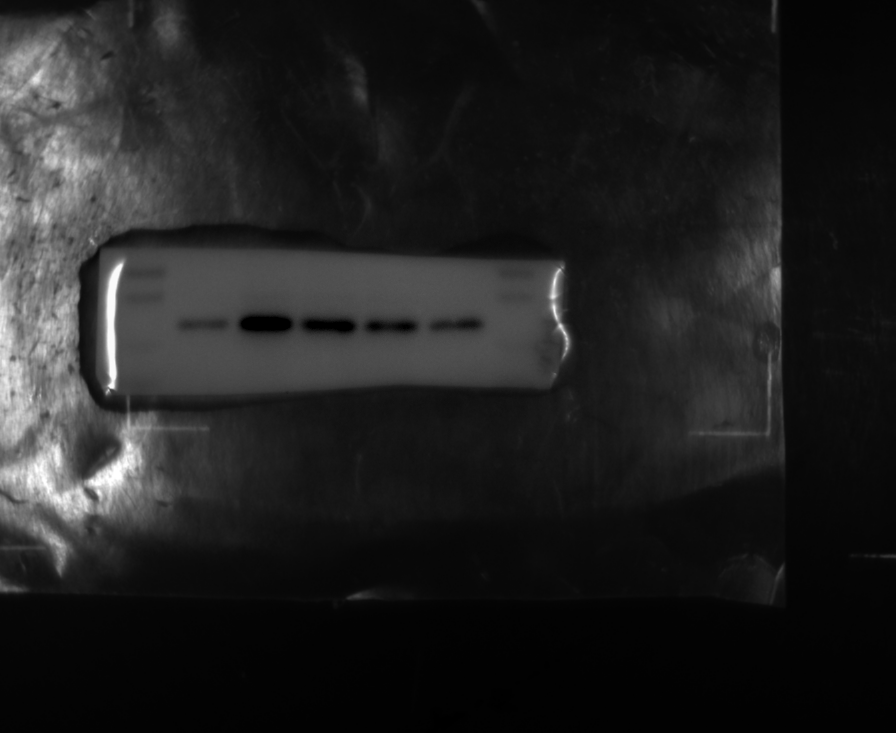

Supplement: Supplementary file 2 — Supplementary Material 2 [file 13062_2026_767_MOESM2_ESM.zip › supplementary file2/Figure 6B_GPX4(2).tif]

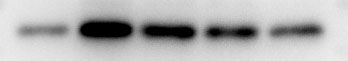

Supplement: Supplementary file 2 — Supplementary Material 2 [file 13062_2026_767_MOESM2_ESM.zip › supplementary file2/Figure 6B_GPX4.jpg]

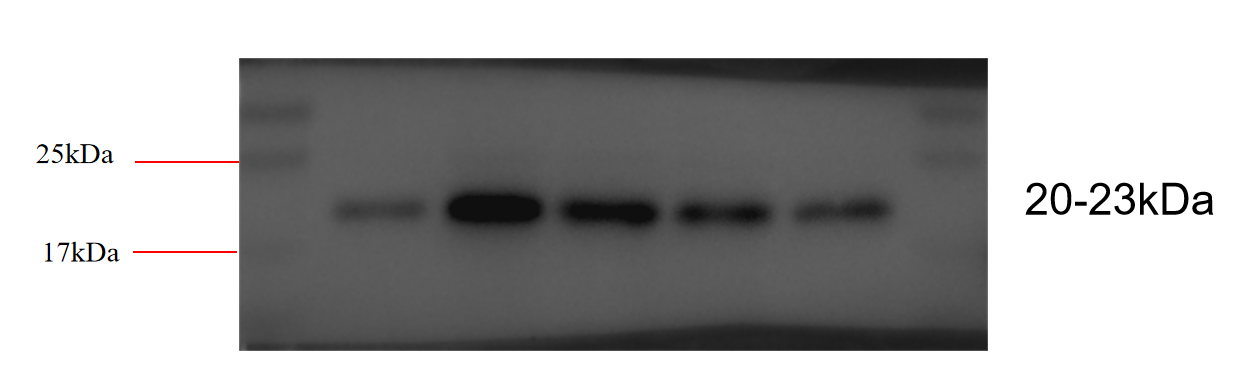

Supplement: Supplementary file 2 — Supplementary Material 2 [file 13062_2026_767_MOESM2_ESM.zip › supplementary file2/Figure 6B_GPX4.tif]

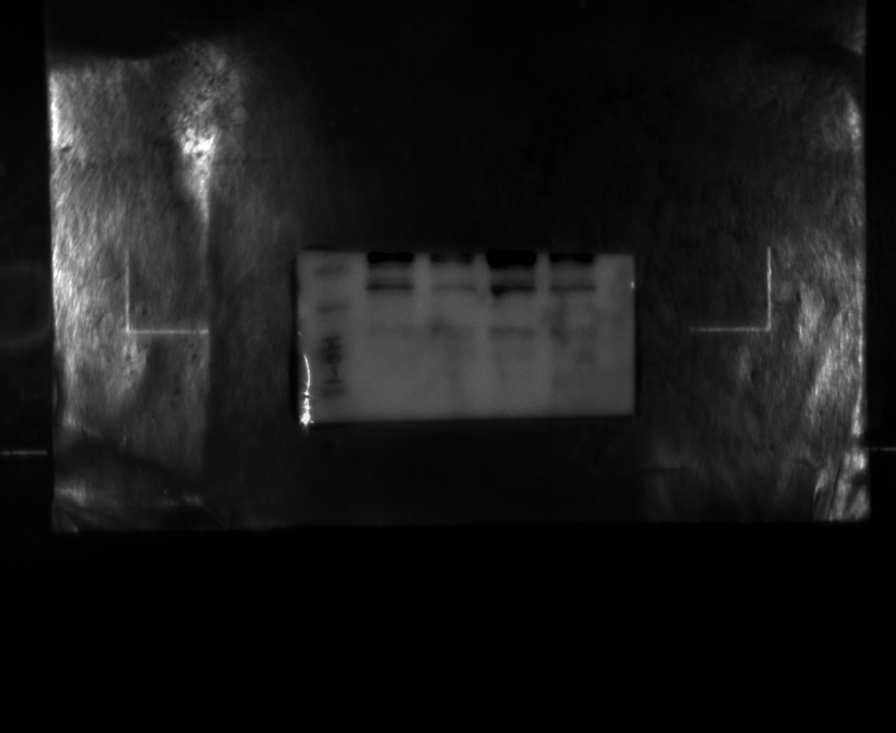

Supplement: Supplementary file 2 — Supplementary Material 2 [file 13062_2026_767_MOESM2_ESM.zip › supplementary file2/Figure 6E_HO-1(2).jpg]

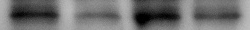

Supplement: Supplementary file 2 — Supplementary Material 2 [file 13062_2026_767_MOESM2_ESM.zip › supplementary file2/Figure 6E_HO-1.jpg]

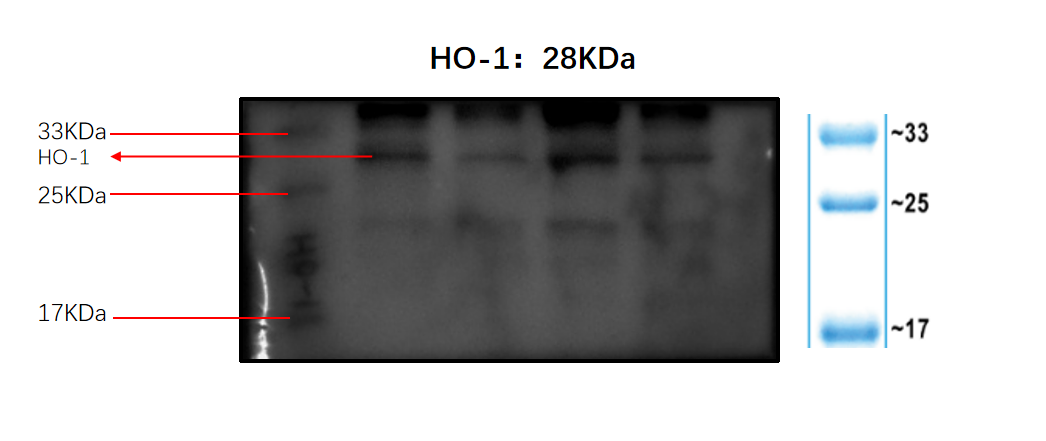

Supplement: Supplementary file 2 — Supplementary Material 2 [file 13062_2026_767_MOESM2_ESM.zip › supplementary file2/Figure 6E_HO-1.tif]

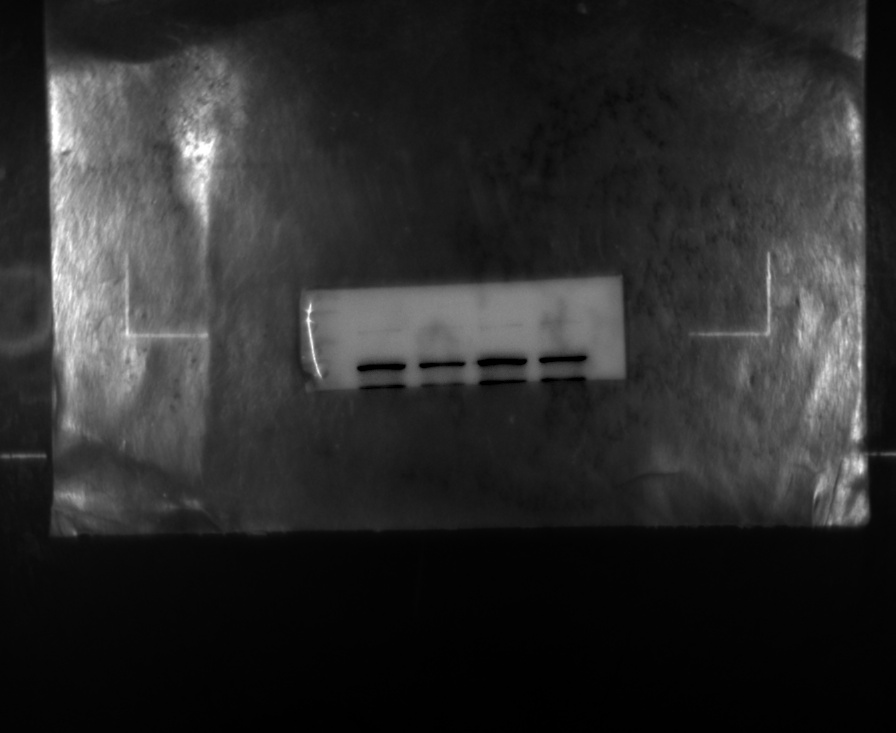

Supplement: Supplementary file 2 — Supplementary Material 2 [file 13062_2026_767_MOESM2_ESM.zip › supplementary file2/Figure 6E_Nrf2(2).jpg]

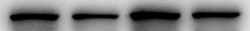

Supplement: Supplementary file 2 — Supplementary Material 2 [file 13062_2026_767_MOESM2_ESM.zip › supplementary file2/Figure 6E_Nrf2.jpg]

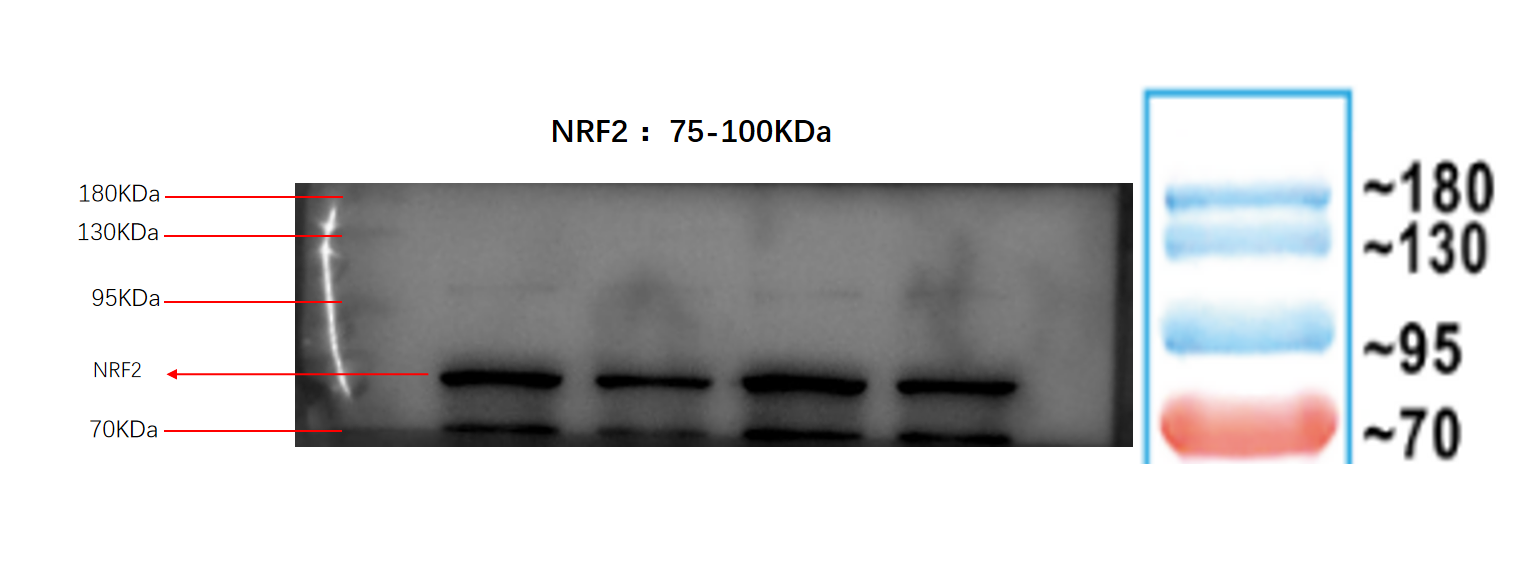

Supplement: Supplementary file 2 — Supplementary Material 2 [file 13062_2026_767_MOESM2_ESM.zip › supplementary file2/Figure 6E_Nrf2.tif]

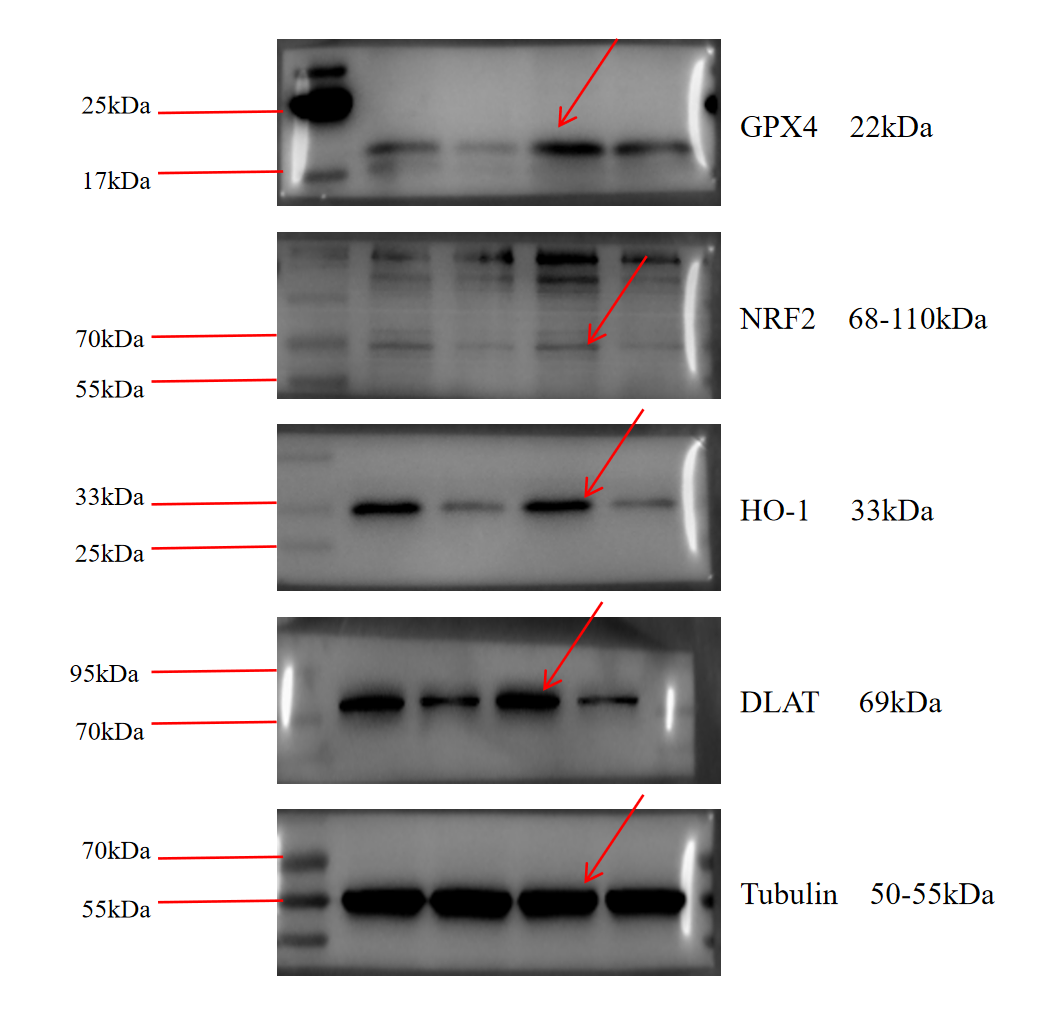

Supplement: Supplementary file 2 — Supplementary Material 2 [file 13062_2026_767_MOESM2_ESM.zip › supplementary file2/Figure 6F.tif]

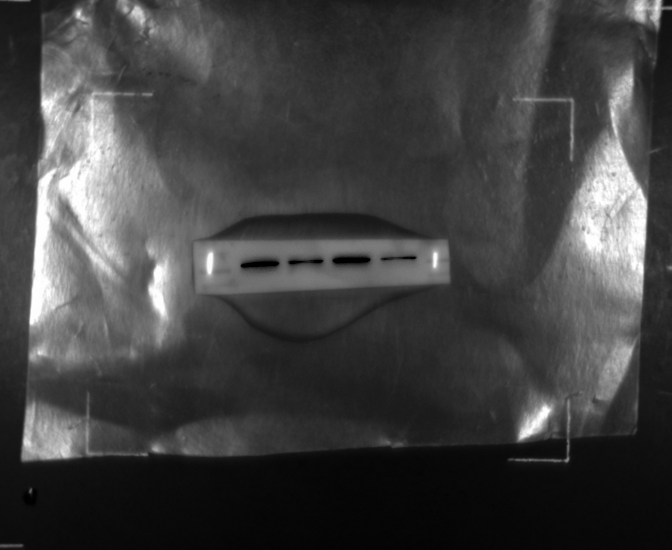

Supplement: Supplementary file 2 — Supplementary Material 2 [file 13062_2026_767_MOESM2_ESM.zip › supplementary file2/Figure 6F_DLAT(2).tif]

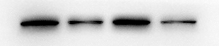

Supplement: Supplementary file 2 — Supplementary Material 2 [file 13062_2026_767_MOESM2_ESM.zip › supplementary file2/Figure 6F_DLAT.tif]

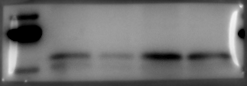

Supplement: Supplementary file 2 — Supplementary Material 2 [file 13062_2026_767_MOESM2_ESM.zip › supplementary file2/Figure 6F_GPX4(2).tif]

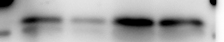

Supplement: Supplementary file 2 — Supplementary Material 2 [file 13062_2026_767_MOESM2_ESM.zip › supplementary file2/Figure 6F_GPX4.tif]

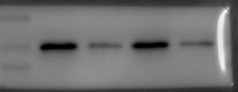

Supplement: Supplementary file 2 — Supplementary Material 2 [file 13062_2026_767_MOESM2_ESM.zip › supplementary file2/Figure 6F_HO-1(2).tif]

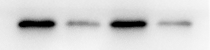

Supplement: Supplementary file 2 — Supplementary Material 2 [file 13062_2026_767_MOESM2_ESM.zip › supplementary file2/Figure 6F_HO-1.tif]

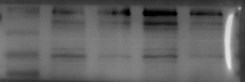

Supplement: Supplementary file 2 — Supplementary Material 2 [file 13062_2026_767_MOESM2_ESM.zip › supplementary file2/Figure 6F_Nrf2(2).tif]

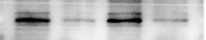

Supplement: Supplementary file 2 — Supplementary Material 2 [file 13062_2026_767_MOESM2_ESM.zip › supplementary file2/Figure 6F_Nrf2.tif]

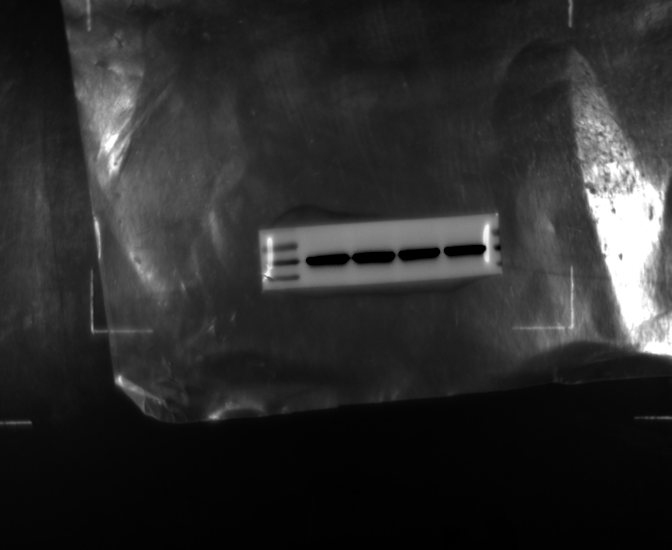

Supplement: Supplementary file 2 — Supplementary Material 2 [file 13062_2026_767_MOESM2_ESM.zip › supplementary file2/Figure 6F_Tubulin(2).tif]

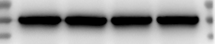

Supplement: Supplementary file 2 — Supplementary Material 2 [file 13062_2026_767_MOESM2_ESM.zip › supplementary file2/Figure 6F_Tubulin.tif]

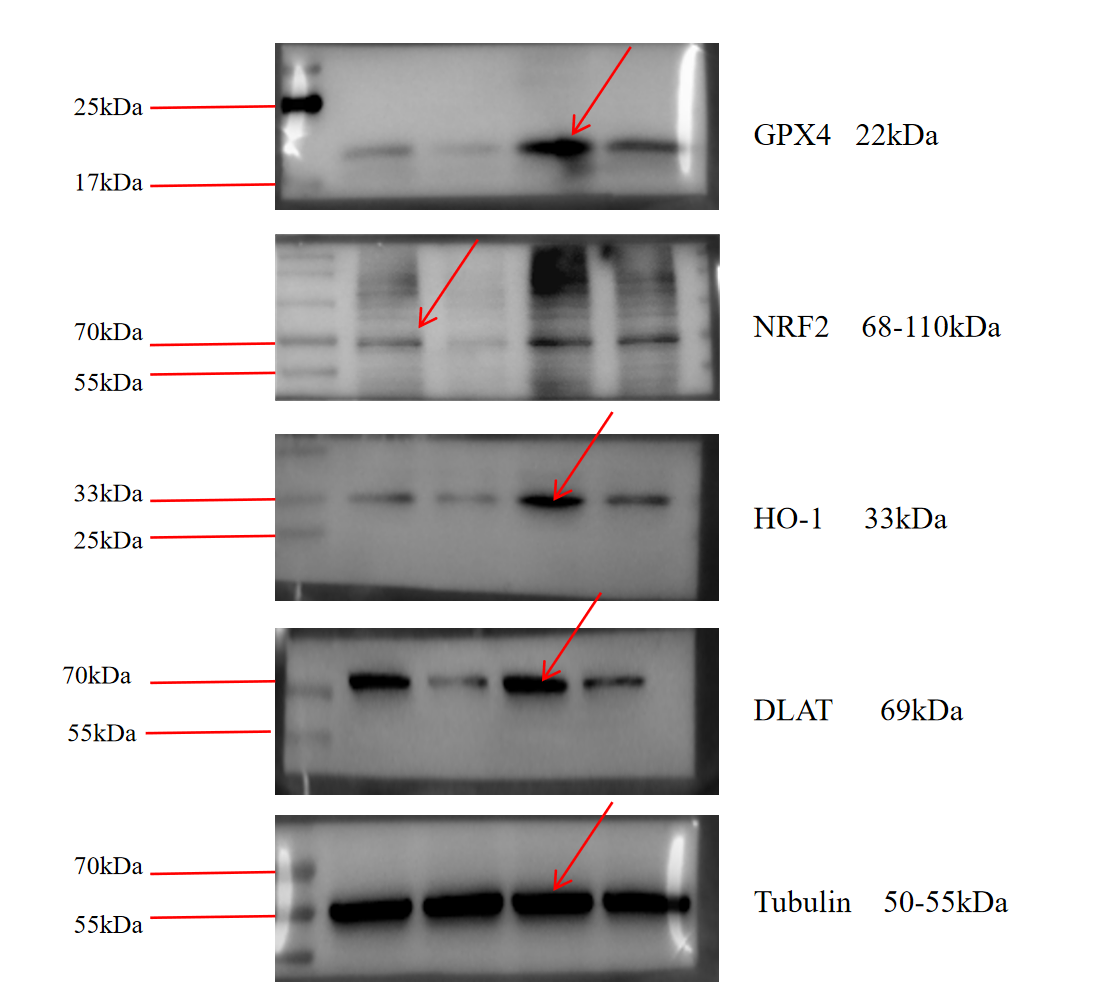

Supplement: Supplementary file 2 — Supplementary Material 2 [file 13062_2026_767_MOESM2_ESM.zip › supplementary file2/Figure 6G.tif]

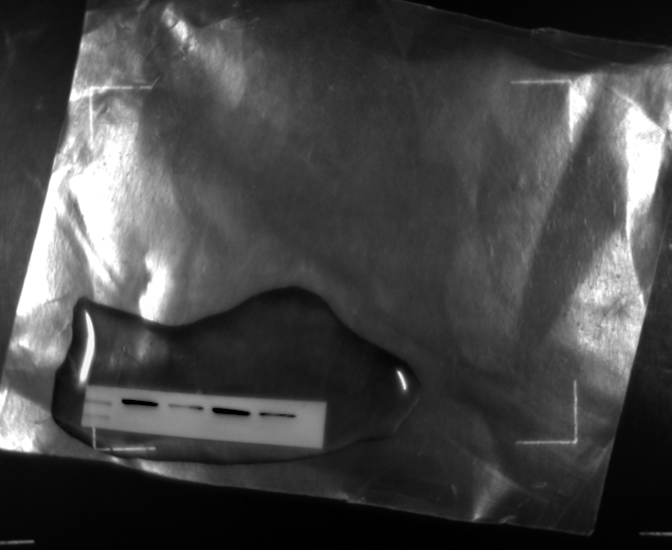

Supplement: Supplementary file 2 — Supplementary Material 2 [file 13062_2026_767_MOESM2_ESM.zip › supplementary file2/Figure 6G_DLAT(2).tif]

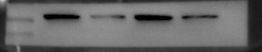

Supplement: Supplementary file 2 — Supplementary Material 2 [file 13062_2026_767_MOESM2_ESM.zip › supplementary file2/Figure 6G_DLAT(3).tif]

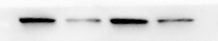

Supplement: Supplementary file 2 — Supplementary Material 2 [file 13062_2026_767_MOESM2_ESM.zip › supplementary file2/Figure 6G_DLAT.tif]

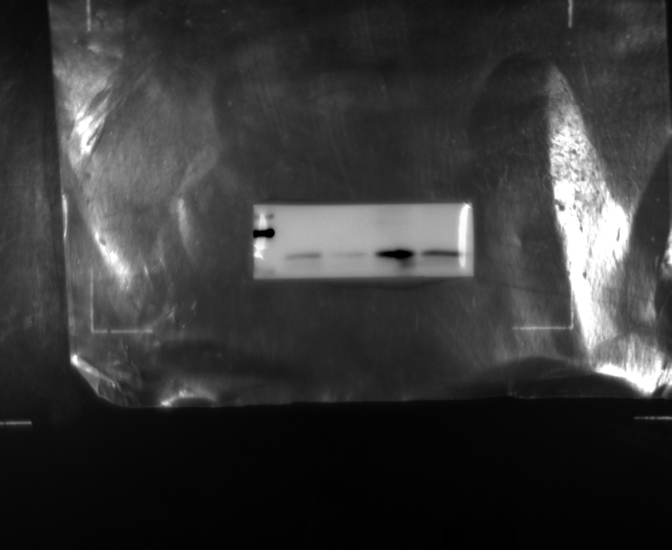

Supplement: Supplementary file 2 — Supplementary Material 2 [file 13062_2026_767_MOESM2_ESM.zip › supplementary file2/Figure 6G_GPX4(2).tif]

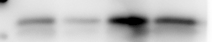

Supplement: Supplementary file 2 — Supplementary Material 2 [file 13062_2026_767_MOESM2_ESM.zip › supplementary file2/Figure 6G_GPX4.tif]

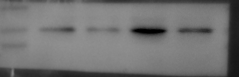

Supplement: Supplementary file 2 — Supplementary Material 2 [file 13062_2026_767_MOESM2_ESM.zip › supplementary file2/Figure 6G_HO-1(2).tif]

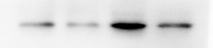

Supplement: Supplementary file 2 — Supplementary Material 2 [file 13062_2026_767_MOESM2_ESM.zip › supplementary file2/Figure 6G_HO-1.tif]

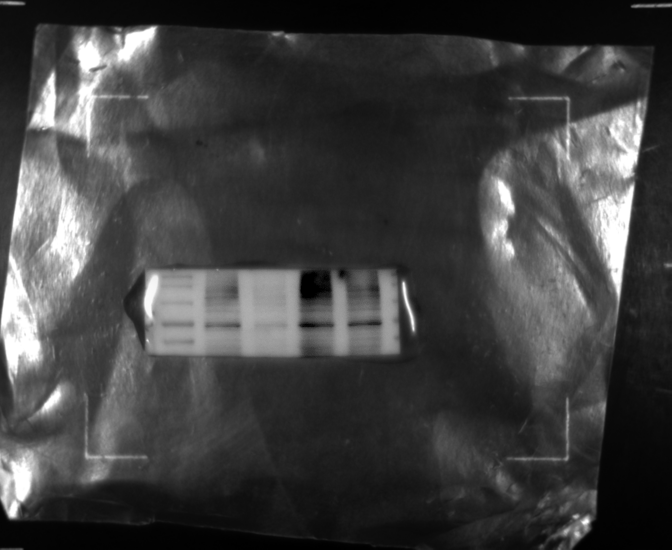

Supplement: Supplementary file 2 — Supplementary Material 2 [file 13062_2026_767_MOESM2_ESM.zip › supplementary file2/Figure 6G_Nrf2(2).tif]

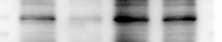

Supplement: Supplementary file 2 — Supplementary Material 2 [file 13062_2026_767_MOESM2_ESM.zip › supplementary file2/Figure 6G_Nrf2.tif]

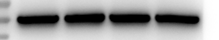

Supplement: Supplementary file 2 — Supplementary Material 2 [file 13062_2026_767_MOESM2_ESM.zip › supplementary file2/Figure 6G_Tubulin(2).tif]

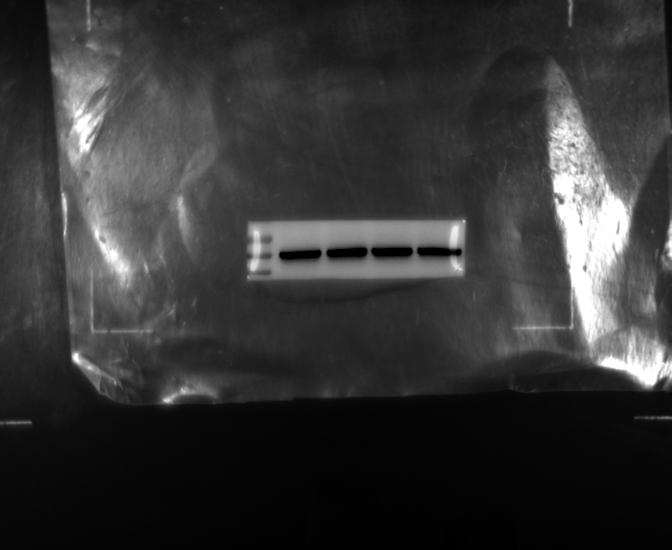

Supplement: Supplementary file 2 — Supplementary Material 2 [file 13062_2026_767_MOESM2_ESM.zip › supplementary file2/Figure 6G_Tubulin.tif]

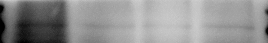

Supplement: Supplementary file 2 — Supplementary Material 2 [file 13062_2026_767_MOESM2_ESM.zip › supplementary file2/Figure 7B_DLAT (2).tif]

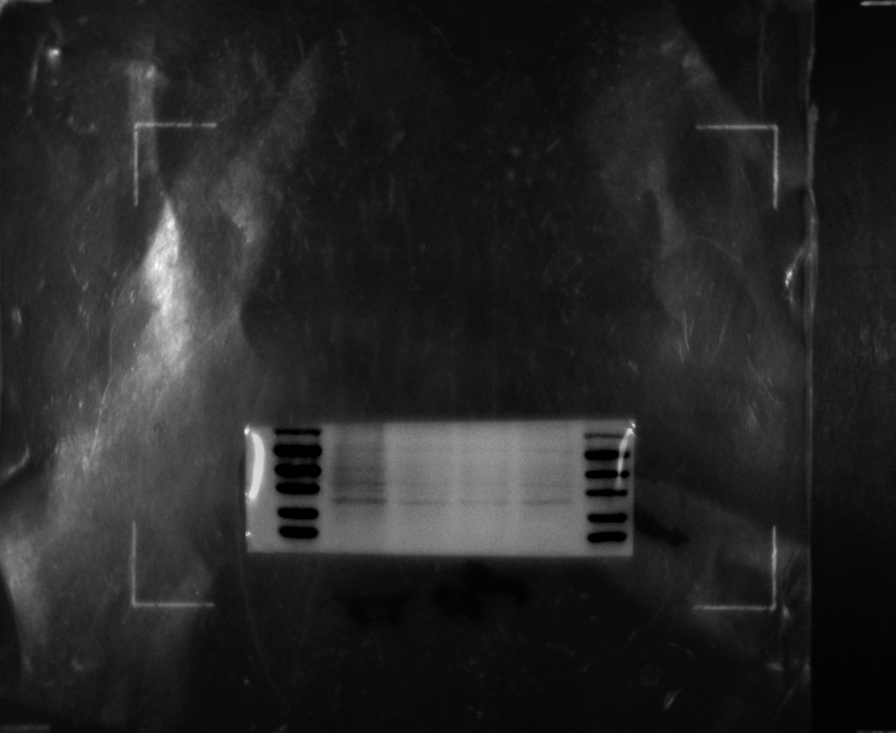

Supplement: Supplementary file 2 — Supplementary Material 2 [file 13062_2026_767_MOESM2_ESM.zip › supplementary file2/Figure 7B_DLAT (3).tif]

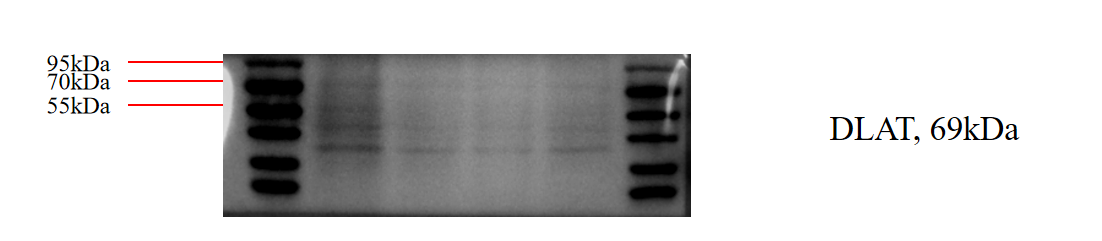

Supplement: Supplementary file 2 — Supplementary Material 2 [file 13062_2026_767_MOESM2_ESM.zip › supplementary file2/Figure 7B_DLAT.tif]

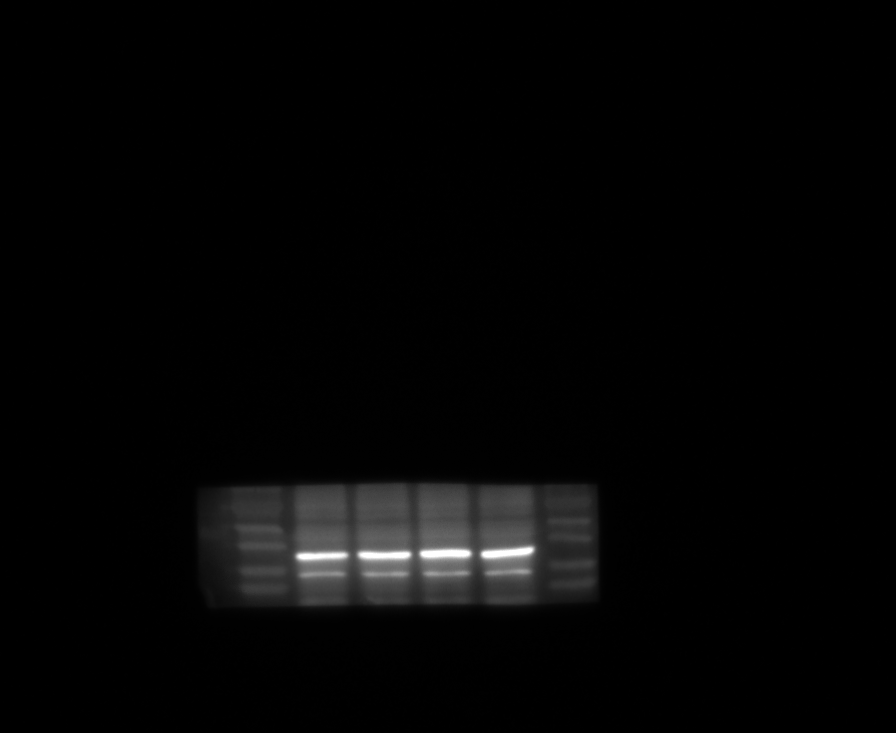

Supplement: Supplementary file 2 — Supplementary Material 2 [file 13062_2026_767_MOESM2_ESM.zip › supplementary file2/Figure 7B_tubulin (2).TIF]

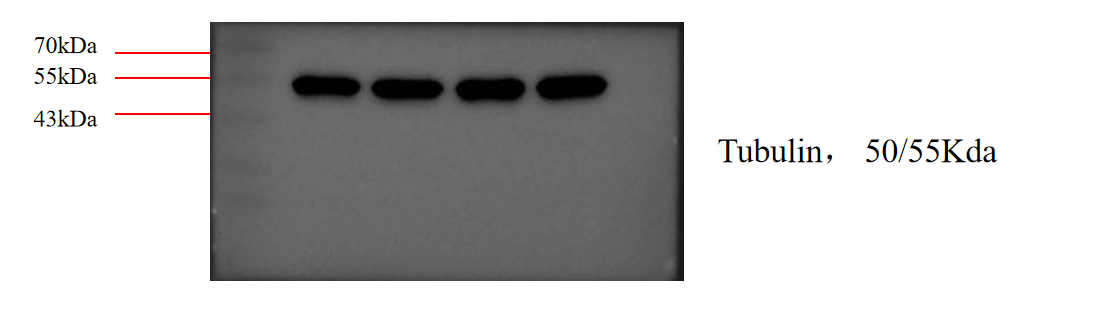

Supplement: Supplementary file 2 — Supplementary Material 2 [file 13062_2026_767_MOESM2_ESM.zip › supplementary file2/Figure 7B_tubulin.tif]

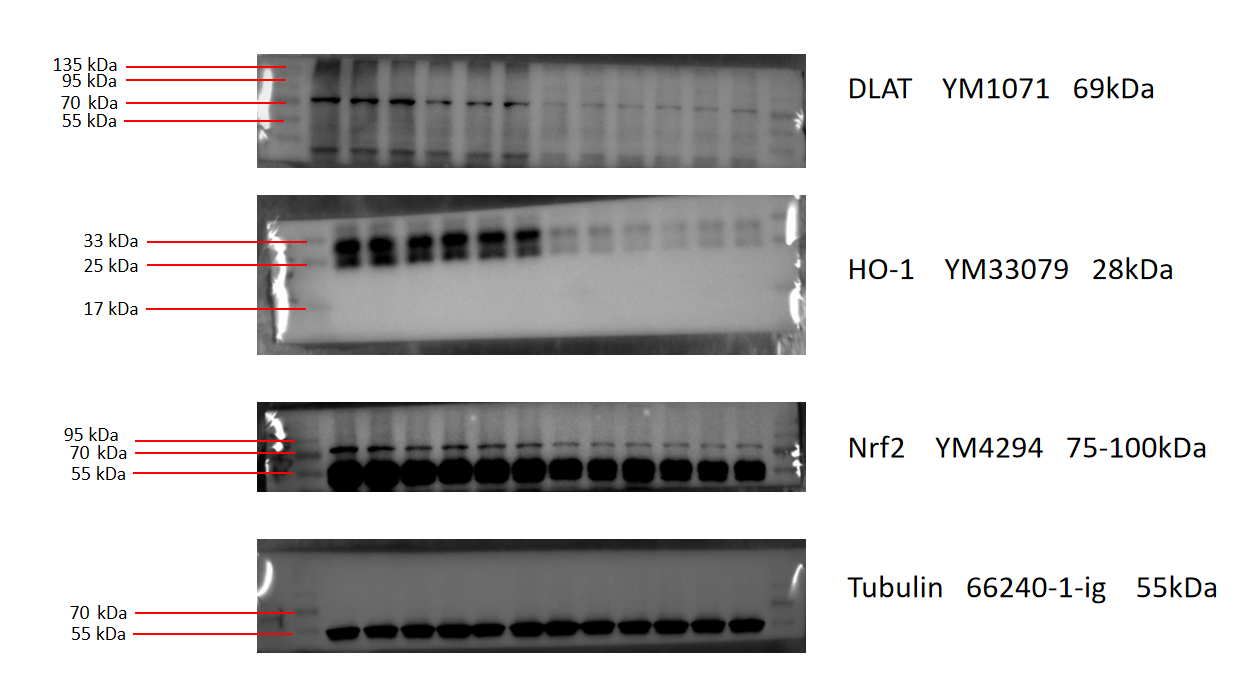

Supplement: Supplementary file 2 — Supplementary Material 2 [file 13062_2026_767_MOESM2_ESM.zip › supplementary file2/Figure 7F.tif]

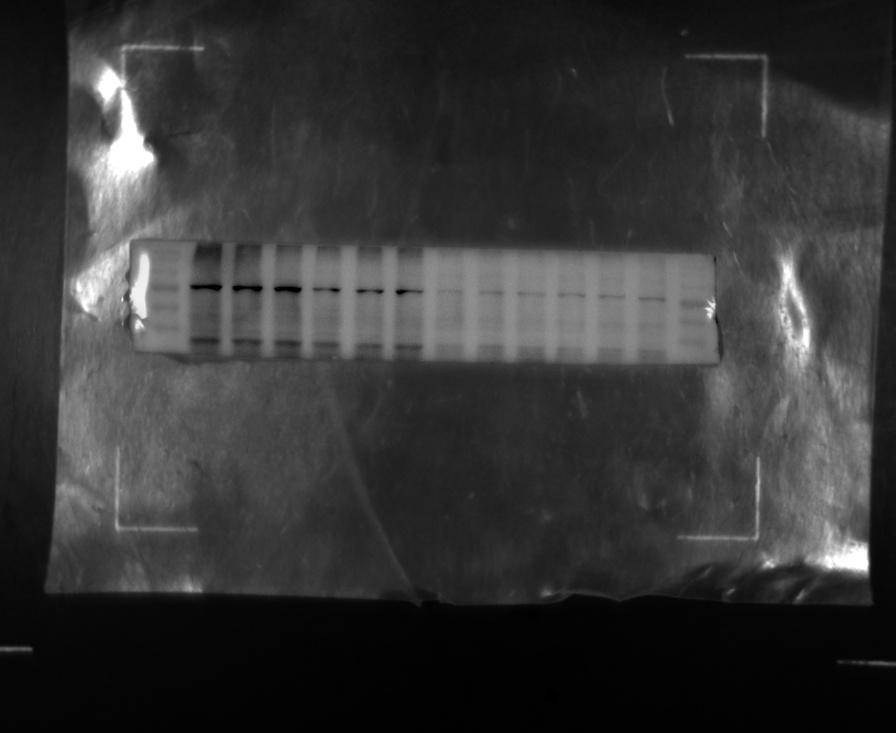

Supplement: Supplementary file 2 — Supplementary Material 2 [file 13062_2026_767_MOESM2_ESM.zip › supplementary file2/Figure 7F_DLAT (2).jpg]

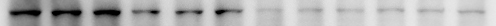

Supplement: Supplementary file 2 — Supplementary Material 2 [file 13062_2026_767_MOESM2_ESM.zip › supplementary file2/Figure 7F_DLAT.jpg]

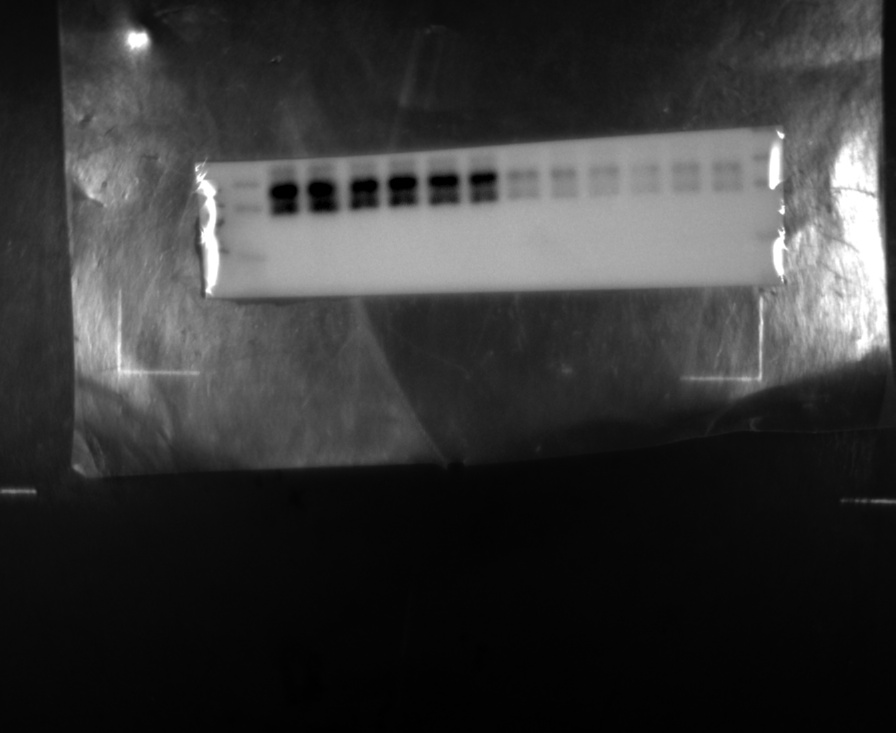

Supplement: Supplementary file 2 — Supplementary Material 2 [file 13062_2026_767_MOESM2_ESM.zip › supplementary file2/Figure 7F_HO-1 (2).jpg]

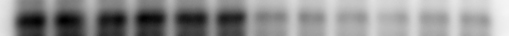

Supplement: Supplementary file 2 — Supplementary Material 2 [file 13062_2026_767_MOESM2_ESM.zip › supplementary file2/Figure 7F_HO-1.jpg]

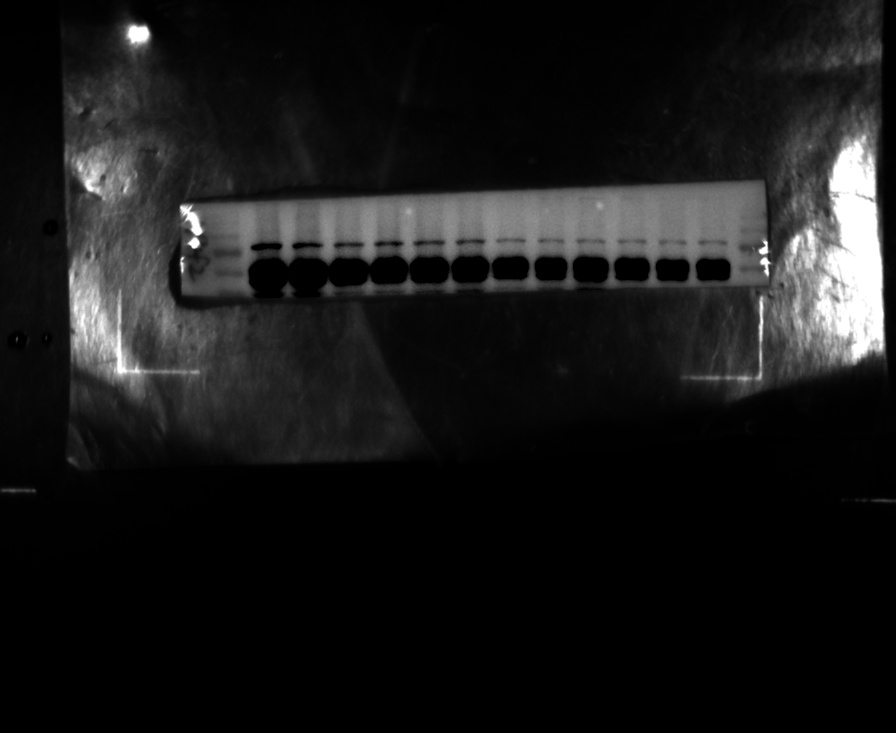

Supplement: Supplementary file 2 — Supplementary Material 2 [file 13062_2026_767_MOESM2_ESM.zip › supplementary file2/Figure 7F_Nrf2 (2).jpg]

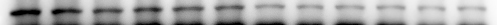

Supplement: Supplementary file 2 — Supplementary Material 2 [file 13062_2026_767_MOESM2_ESM.zip › supplementary file2/Figure 7F_Nrf2.jpg]

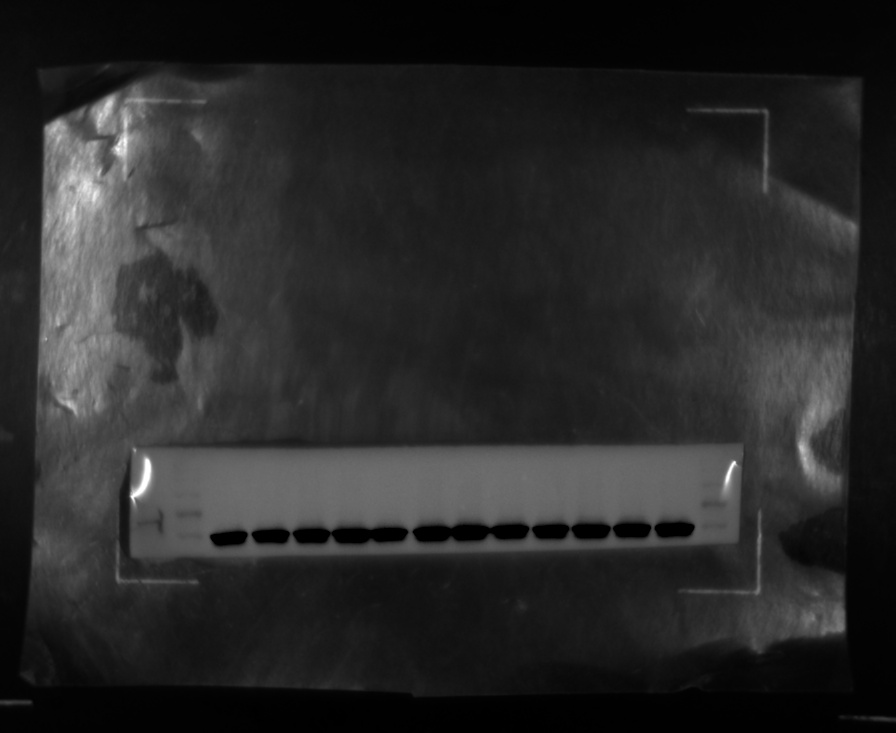

Supplement: Supplementary file 2 — Supplementary Material 2 [file 13062_2026_767_MOESM2_ESM.zip › supplementary file2/Figure 7F_tubulin (2).jpg]

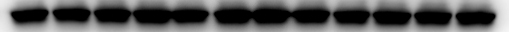

Supplement: Supplementary file 2 — Supplementary Material 2 [file 13062_2026_767_MOESM2_ESM.zip › supplementary file2/Figure 7F_tubulin.jpg]
